# Supplementary material for: Mediation role of perceived social support between recurrence risk perception and health behaviour among patients with stroke in China: a cross-sectional study
Source: BMJ Open. 2024 Feb 13;14(2):e079812. doi: 10.1136/bmjopen-2023-079812 (PMC10868314; doi:10.1136/bmjopen-2023-079812)
Supplement: Supplementary data [file bmjopen-2023-079812supp001.pdf]

Supplemental Table 1 Comparison of recurrence risk perception scores of stroke patients with different characteristics

Factors	Group	*N*	%	SRRPS	Perceived illness risk factors	Perceived behavioral risk factors	Perceived severity
Gender	Male	136	54.4	40.88±9.73	14.68±4.03	8.71±2.38	17.43±4.45
	Female	114	45.6	41.23±7.15	14.66±3.60	8.24±2.30	18.33±3.48
Marriage	Married	220	88	41.46±8.23	14.92±3.71	8.60±2.29	17.95±3.85
	Divorced	2	0.8	47.00±0.00	17.00±1.41	9.50±2.12	20.50±0.71
	Unmarried	28	11.2	37.29±10.91	12.50±4.24	7.71±2.79	16.82±5.40
Age	＜60	73	29.2	44.55±6.44	15.93±2.83	9.59±2.06	19.03±2.93
	≥60	177	70.8	39.59±9.02	14.15±4.07	8.04±2.32	17.84±4.05
Residential area	Rural	126	50.4	39.92±7.91	14.08±3.91	8.10±2.17	17.68±3.79
	Urban	124	49.6	42.17±9.21	15.27±3.68	8.89±2.46	18.01±4.32
Working state	Unemployed	54	21.6	40.11±7.29	13.48±3.30	7.85±2.28	18.78±3.66
	Pensioner	109	43.6	39.37±9.98	14.16±4.22	8.18±2.47	17.03±4.66
	Working	87	34.8	43.70±6.85	16.06±3.22	9.28±2.04	18.29±3.23
Educational level	Primary and below	115	46	38.72±7.99	13.56±3.97	7.82±2.06	17.29±4.06
	Junior high school	65	27.6	40.49±8.61	14.75±3.35	8.22±2.28	17.52±4.28
	High school	44	17.6	42.81±9.35	15.50±3.99	9.11±2.46	18.20±4.18
	College	14	5.6	48.64±2.21	18.00±0.00	10.57±1.60	20.07±2.06
	University and above	12	4.8	50.75±0.45	18.00±0.00	11.75±0.45	21.00±0.00
Stroke type	Hemorrhage stroke	6	2.4	47.50±3.74	17.25±1.04	10.00±2.14	20.25±1.04
	Ischemic stroke	244	97.6	40.82±8.67	14.59±3.86	8.44±2.34	17.76±4.09

Family history	Have	25	10	38.36±10.60	13.32±4.96	8.08±2.33	16.96±4.33
	No	255	90	41.33±8.37	14.82±3.67	8.54±2.35	17.94±4.02
Monthly income (RMB)	≤2999	89	35.6	37.62±7.98	13.08±3.81	7.56±2.00	16.90±4.22
	3000-4999	126	50.4	42.59±8.39	15.52±3.64	8.75±2.27	18.32±3.90
	≥5000	35	14	44.14±8.56	15.69±3.31	9.91±2.56	18.54±3.85
Numbers of stroke	1 time	130	52	41.54±8.19	15.38±3.42	8.78±2.05	17.38±3.99
	2 times	74	29.6	43.15±6.12	15.04±3.18	8.88±2.16	19.23±2.70
	3 times	22	8.8	42.27±8.14	14.27±4.08	8.27±3.01	19.41±3.70
	More than 3 times	24	9.6	30.67±11.07	10.08±4.57	5.92±2.30	14.67±5.73
Primary caregiver	Spouse or children	132	52.8	17.30±4.46	14.30±4.16	8.17±2.42	39.82±9.54
	Babysitter or nursing workers	11	4.4	38.00±11.12	14.73±4.47	14.73±4.47	15.73±5.10
	Own	107	42.8	42.85±6.71	15.12±3.31	8.99±2.22	18.74±3.15
Number of chronic diseases	0	75	30	40.15±8.96	14.07±3.80	8.15±2.15	17.93±3.94
	1~2	150	60	41.29±9.17	14.75±4.04	8.67±2.43	17.83±4.17
	3 and above	25	10	42.20±6.87	16.04±1.86	8.48±2.40	17.68±3.87
Sleep conditions	Very good	92	36.8	40.07±9.91	14.12±4.20	8.35±2.55	17.52±4.60
	Good	69	27.6	42.51±8.25	15.25±3.78	8.81±2.37	18.45±3.47
	Poor	69	27.6	39.65±7.86	14.23±3.61	8.28±2.28	17.14±4.21
	Very poor	20	8	45.20±2.19	16.75±1.55	8.80±1.32	19.65±0.88

Supplemental Table 2 Comparison of perceived social support scores of stroke patients with different characteristics

Factors	Group	*N*	%	PSSS	Family support	Friend support	Other support
Gender	Male	136	54.4	65.50±9.07	22.65±3.89	20.43±4.32	22.42±3.48
	Female	114	45.6	66.29±8.43	23.60±3.11	19.41±5.22	23.28±3.15
Marriage	Married	220	88	66.27±8.55	23.17±3.49	20.15±4.84	22.95±3.15
	Divorced	2	0.8	69.00±12.73	22.50±3.54	23.00±4.24	23.50±4.95
	Unmarried	28	11.2	62.43±9.88	22.39±4.26	18.36±3.96	21.68±4.60
Gender	<60	73	29.2	68.64±8.56	23.90±2.92	21.11±5.21	23.63±2.74
	≥60	177	70.8	64.71±8.63	22.75±3.78	19.50±4.51	22.47±3.52
Residential area	Rural	126	50.4	65.54±8.14	23.23±3.08	19.37±4.90	22.94±3.31
	Urban	124	49.6	66.19±9.40	22.94±4.04	20.58±4.57	22.67±3.40
Working state	Unemployed	54	21.6	64.33±8.70	22.67±3.39	19.57±4.96	22.09±3.54
	Pensioner	109	43.6	65.01±8.81	22.76±3.82	19.71±4.78	22.54±3.34
	Working	87	34.8	67.87±8.51	23.75±3.32	20.54±4.64	23.59±3.12
Educational level	Primary and below	115	46	64.03±7.79	22.57±3.31	18.92±4.90	22.53±3.06
	Junior high school	65	27.6	64.07±8.83	22.32±3.79	19.77±4.17	21.97±3.82
	High school	44	17.6	68.37±8.74	23.80±3.57	20.95±4.73	23.61±3.19
	College	14	5.6	71.36±7.96	25.36±3.03	21.29±3.95	24.71±3.20
	University and above	12	4.8	77.58±4.54	26.83±1.57	25.92±.151	25.83±2.04
Stroke type	Hemorrhagic stroke	6	2.4	73.88±7.38	26.50±1.60	21.63±5.58	25.75±1.16
	Ischemic stroke	244	97.6	65.60±8.71	22.97±3.57	19.91±4.74	22.71±3.36
Family history	Have	25	10	66.00±8.56	23.28±2.57	19.24±5.33	23.49±3.38
	No	255	90	65.84±8.82	23.06±3.68	20.05±4.71	22.73±3.35
Monthly income (RMB)	≤2999	89	35.6	64.73±8.07	22.79±3.48	19.10±4.46	33.84±3.44

	3000-4999	126	50.4	65.67±8.59	23.09±3.46	19.94±5.01	22.65±3.08
	≥5000	35	14	69.40±10.38	23.83±4.22	22.23±3.92	23.29±4.06
Numbers of stroke	1 time	130	52	65.88±9.05	22.90±3.62	20.18±4.84	22.80±3.35
	2 times	74	29.6	67.03±7.36	23.88±2.91	20.18±4.63	22.97±2.93
	3 times	22	8.8	67.27±9.19	23.59±3.65	20.32±4.47	23.36±3.47
	More than 3 times	24	9.6	60.83±9.63	21.17±4.45	17.83±4.82	21.83±4.34
Primary caregiver	Spouse or children	132	52.8	64.95±8.77	23.23±3.77	18.86±5.03	22.86±3.54
	Babysitter or nursing workers	11	4.4	58.73±8.00	19.64±3.80	19.55±1.51	19.55±4.25
	Own	107	42.8	67.71±8.37	23.25±3.15	21.37±4.29	23.08±2.83
Number of chronic diseases	0	75	30	66.16±7.92	23.17±3.38	19.52±5.07	23.47±3.04
	1~2	150	60	65.12±9.16	22.78±3.72	20.05±4.63	22.29±3.51
	3 and above	25	10	69.40±8.24	24.64±2.97	20.84±4.67	23.92±2.69
Sleep conditions	Very good	92	36.8	65.48±9.27	22.83±4.07	19.86±5.14	22.79±3.34
	Good	69	27.6	68.55±8.27	24.11±3.00	20.75±4.73	23.68±2.96
	Poor	69	27.6	63.55±8.66	22.29±3.53	19.22±4.54	22.04±3.81
	Very poor	20	8	66.30±6.16	23.45±2.26	20.35±3.66	22.50±2.40

Supplemental Table 3 Comparison of health behavior scores of stroke patients with different characteristics

Factors	Group	*N*	%	HBS-SP	Exercise	Medication taking	Instructions	Nutrition	Responsibility	Smoking and alcohol
Gender	Male	136	54.4	56.72±9.07	11.71±4.6	12.00±2.73	8.65±2.48	14.37±4.03	3.30±0.83	6.68±1.59
	Female	114	45.6	56.84±9.01	11.97±5.01	11.42±2.55	9.00±2.70	14.61±3.77	3.19±0.58	6.63±1.82
Marriage	Married	220	88	57.12±8.84	12.04±4.79	11.72±2.55	8.94±2.66	14.60±3.91	3.25±0.74	6.57±1.73
	Divorced	2	0.8	59.50±3.54	12.50±0.71	11.50±0.71	10.00±2.83	15.00±1.41	3.50±0.71	7.00±1.41
	Unmarried	28	11.2	53.86±10.33	10.10±4.86	11.82±3.53	7.93±1.68	13.29±3.80	3.32±0.67	7.39±1.13
Age	<60	73	29.2	60.47±9.38	13.88±5.14	12.23±2.58	8.60±2.72	15.88±3.83	3.45±1.09	6.42±1.79
	≥60	177	70.8	55.25±8.44	10.99±4.42	11.54±2.67	8.90±2.52	13.90±3.80	3.17±0.49	6.76±1.65
Residential area	Rural	126	50.4	55.83±7.44	11.84±4.69	11.55±2.63	8.84±2.27	13.93±3.40	3.17±0.72	6.49±1.77
	Urban	124	49.6	57.74±10.34	11.82±4.95	11.94±2.68	8.78±2.86	15.04±4.31	3.33±0.74	6.83±1.59
Working state	Unemployed	54	21.6	55.30±8.14	12.43±4.90	11.37±2.53	8.54±2.34	13.67±3.19	3.00±0.00	6.30±1.89
	Pensioner	109	43.6	55.55±8.75	10.81±4.49	11.55±2.56	8.95±2.74	14.23±4.16	3.18±0.49	6.83±1.52
	Working	87	34.8	59.23±9.46	12.75±4.96	12.21±2.82	8.82±2.54	15.30±3.87	3.49±1.06	6.67±1.75
Educational level	Primary and below	115	46	54.56±7.09	11.47±4.47	11.13±2.53	8.69±2.23	13.47±3.27	3.08±0.35	6.72±1.76
	Junior high school	65	27.6	55.14±8.66	11.46±4.06	11.65±2.78	8.83±2.45	13.75±3.96	3.11±0.31	6.34±1.71
	High school	44	17.6	58.80±9.69	10.61±5.11	12.57±2.73	8.39±3.33	16.64±4.37	3.27±0.62	7.32±1.07
	College	14	5.6	63.64±8.72	15.43±3.82	13.07±2.06	9.86±3.08	16.07±3.00	3.57±1.01	5.64±2.27

	University and above	12	4.8	71.50±7.01	17.58±6.42	13.50±1.62	10.25±2.22	18.33±2.53	5.25±1.60	6.58±1.16
Stroke type	Hemorrhagic stroke	6	2.4	64.75±7.94	13.13±6.49	13.13±2.17	11.75±2.05	16.38±2.92	3.00±0.00	7.38±0.91
	Ischemic stroke	244	97.6	56.51±8.95	11.79±4.76	11.69±2.67	8.71±2.54	14.42±3.93	3.26±0.74	6.63±1.71
Family history	Have	25	10	57.68±9.39	13.00±3.81	11.00±2.02	10.16±2.73	13.76±4.15	3.12±0.33	6.64±1.63
	No	255	90	56.68±9.00	11.70±4.90	11.82±2.71	8.66±2.53	14.56±3.88	3.27±0.76	6.66±1.70
Monthly income (RMB)	≤2999	89	35.6	53.47±6.64	10.85±4.20	11.28±2.69	8.58±2.48	13.12±3.46	3.03±0.18	6.60±1.87
	3000-4999	126	50.4	57.63±8.78	12.09±4.89	11.87±2.47	8.94±2.76	14.85±3.84	3.26±0.67	6.63±1.65
	≥5000	35	14	62.11±11.78	13.40±5.54	12.46±3.07	8.94±2.14	16.60±4.08	3.77±1.33	6.94±1.37
Numbers of stroke	1 time	130	52	57.08±9.51	11.77±4.59	11.77±2.73	8.40±2.71	15.00±4.12	3.34±0.87	6.80±1.65
	2 times	74	29.6	57.69±7.11	13.11±4.78	11.58±2.32	9.23±2.41	14.23±3.02	3.18±0.53	6.36±1.72
	3 times	22	8.8	59.32±9.24	12.64±5.48	12.95±2.50	9.82±2.26	14.64±3.17	3.27±0.77	6.00±2.00
	More than 3 times	24	9.6	50.00±8.90	7.50±2.72	10.96±3.13	8.83±2.32	12.29±5.01	3.00±0.00	7.42±1.10
Primary caregiver	Spouse or children	132	52.8	55.58±8.50	10.40±4.48	11.95±2.78	8.63±2.70	14.67±4.31	3.24±0.80	6.69±1.69
	Babysitter or nursing workers	11	4.4	52.36±10.27	9.64±2.87	9.45±2.34	9.18±2.27	12.91±3.36	3.73±0.79	7.45±1.04
	Own	107	42.8	58.71±9.19	13.82±4.66	11.72±2.44	9.00±2.46	14.41±3.40	3.22±0.61	6.54±1.74
Number of chronic	0	75	30	57.47±7.61	12.75±4.69	12.16±2.38	8.92±2.15	14.12±3.11	3.16±0.55	6.36±1.61

diseases										
	1~2	150	60	56.07±9.44	11.35±4.75	11.51±2.84	8.93±2.50	14.34±4.19	3.21±0.61	6.73±1.75
	3 and above	25	10	58.96±10.18	12.00±5.30	11.88±2.20	7.76±3.82	16.40±3.88	3.76±1.42	7.16±1.46
Sleep conditions	Very good	92	36.8	56.11±9.22	11.86±4.79	11.83±2.94	8.68±2.64	13.79±3.91	3.26±0.66	6.68±1.52
	Good	69	27.6	59.35±9.22	12.68±4.70	11.97±2.39	9.23±2.60	15.38±3.88	3.30±0.67	6.78±1.61
	Poor	69	27.6	55.14±9.16	11.12±4.82	11.38±2.63	8.42±2.45	14.45±4.07	3.26±0.94	6.52±2.01
	Very poor	20	8	56.60±4.51	11.25±5.15	11.80±2.33	9.30±2.60	14.65±2.91	3.00±0.00	6.60±1.64
