# Supplementary material for: Mediation role of perceived social support between recurrence risk perception and health behaviour among patients with stroke in China: a cross-sectional study
Source: BMJ Open. 2024 Feb 13;14(2):e079812. doi: 10.1136/bmjopen-2023-079812 (PMC10868314; doi:10.1136/bmjopen-2023-079812)
Supplement: Author's manuscript [file bmjopen-2023-079812.draft_revisions.pdf]

BMJ Open

BMJ Open is committed to open peer review. As part of this commitment we make the peer review history of every article we publish publicly available.

When an article is published we post the peer reviewers' comments and the authors' responses online. We also post the versions of the paper that were used during peer review. These are the versions that the peer review comments apply to.

The versions of the paper that follow are the versions that were submitted during the peer review process. They are not the versions of record or the final published versions. They should not be cited or distributed as the published version of this manuscript.

BMJ Open is an open access journal and the full, final, typeset and author-corrected version of record of the manuscript is available on our site with no access controls, subscription charges or pay-per-view fees (http://bmjopen.bmj.com).

If you have any questions on BMJ Open's open peer review process please email info.bmjopen@bmj.com

BMJ Open

Relationship between Recurrence Risk Perception and Health Behavior in patients with stroke: the Mediating Role of Perceived Social Support

Journal:	*BMJ Open*
Manuscript ID	bmjopen-2023-079812
Article Type:	Original research
Date Submitted by the Author:	13-Sep-2023

Complete List of Authors:	Wang, Xiaoxuan; Zhengzhou University,<br>Zhang, Zhen-xiang; Zhengzhou University,<br>LIN, Bei-lei; Zhengzhou University<br>Wang, Wenna; Zhengzhou University,<br>MEI, YONG-XIA; Zhengzhou University, School of Nursing and health<br>Zhang, Chunhui; Nursing and Health school, Zhengzhou University<br>Zhang, Qiushi; Zhengzhou University, Department of Fundmental Nursing<br>CHEN, Su-yan; Zhengzhou University

Keywords:	Stroke < NEUROLOGY, Behavior, Chronic Disease

SCHOLARONE™
Manuscripts

I, the Submitting Author has the right to grant and does grant on behalf of all authors of the Work (as defined in the below author licence), an exclusive licence and/or a non-exclusive licence for contributions from authors who are: i) UK Crown employees; ii) where BMJ has agreed a CC-BY licence shall apply, and/or iii) in accordance with the terms applicable for US Federal Government officers or employees acting as part of their official duties; on a worldwide, perpetual, irrevocable, royalty-free basis to BMJ Publishing Group Ltd ("BMJ") its licensees and where the relevant Journal is co-owned by BMJ to the co-owners of the Journal, to publish the Work in this journal and any other BMJ products and to exploit all rights, as set out in our licence.

The Submitting Author accepts and understands that any supply made under these terms is made by BMJ to the Submitting Author unless you are acting as an employee on behalf of your employer or a postgraduate student of an affiliated institution which is paying any applicable article publishing charge ("APC") for Open Access articles. Where the Submitting Author wishes to make the Work available on an Open Access basis (and intends to pay the relevant APC), the terms of reuse of such Open Access shall be governed by a Creative Commons licence – details of these licences and which Creative Commons licence will apply to this Work are set out in our licence referred to above.

Other than as permitted in any relevant BMJ Author's Self Archiving Policies, I confirm this Work has not been accepted for publication elsewhere, is not being considered for publication elsewhere and does not duplicate material already published. I confirm all authors consent to publication of this Work and authorise the granting of this licence.

For peer review only

Relationship between Recurrence Risk Perception and Health Behavior in patients with stroke: the Mediating Role of Perceived Social Support

Abstract

Objectives To examine whether patients with high recurrence risk perception would have healthier behaviors, and to explore whether perceived social support would function as mediators.

Design This was a cross-sectional study.

Setting The study was conducted in a public tertiary hospital in China.

Participants A total of 254 patients with stroke were invited to participate; 250 patients with stroke completed questionnaires validly.

Primary and secondary outcome measures Questionnaires were administered offline to collect data, consisting of 4 parts: general demographics, and scales related to recurrence risk perception, perceived social support and health behavior. A path analysis and correlation analysis were used to analyse the data.

Results Of 250 patients with stroke, 78.4% were below the medium level. The majority (70.8%) were elderly patients. High recurrence risk perception and high perceived social support were significantly associated with better health behavior (all $P<0.001$). Perceived social support mediated the relationship between recurrence risk perception and health behavior, after controlling for age, sex, education, monthly income in the regression models. And perceived social support had a highest mediation effect with a proportion of mediation up to 59.31%.

Conclusions Recurrence risk perception and perceived social support were influential factors in promoting health behavior. Moreover, the impact of recurrence risk perception on health behavior was partially mediated by perceived social support. Therefore, to enhance the sustainability of health behavior, it is crucial to inform patients with stroke about the risk of recurrence, enabling them to increase their confidence in recovery by taking charge of their illness in order to improve their perceived social support.

Strengths and limitations of this study (1)This study significantly expanded the existing research on the intricate connections between recurrence risk perception, perceived social support, and health behavior in patients with stroke. (2) As this study adopts a cross-sectional survey design, it is important to note that causal inferences cannot be made. (3) Given the impact of COVID-19, this survey was conducted solely in one hospital. Therefore, the generalizability and applicability of the findings may be somewhat limited.

Keywords recurrence risk perception, health behavior, perceived social support, mediation effect, nursing, stroke

Introduction

Globally, the risk of stroke recurrence is a considerable but preventable occurrence. A recent systematic review, comprising 37 studies, unveiled the cumulative recurrence rates of stroke at various time interval: 7.7% at three months, 9.5% at six months, 10.4% at one year, 16.1% at two years, and 16.7% at three years following the initial stroke[1]. Despite the implementation of policies and guidelines in various countries aimed at preventing secondary strokes, there has been no significant decrease in the rates of stroke recurrence[2–4].It has been found that long-term physical inactivity, noncompliance with medication, smoking, excessive alcohol consumption, and various other unhealthy habits and behaviors significantly increase the risk of stroke recurrence[5].

Stroke is a type of life-style disease. The adoption of healthy behaviors emerges as a viable solution for addressing the issue of stroke recurrence. Health behavior refers to the conscious and multifaceted actions undertaken by individuals to maintain or enhance their health. It encompasses various aspects such as personal responsibility, physical activity, social interactions, stress management, and self-fulfillment[6]. For stroke

For peer review only - http://bmjopen.bmj.com/site/about/guidelines.xhtml

patients, the recommended health behaviors aimed at preventing recurrence primarily encompass regular physical exercise, a healthy diet, smoking cessation, and moderate alcohol consumption. The prior research indicated a positive correlation between improved health behaviors and better prognosis among stroke patients[7]. The adoption of healthy behaviors has been demonstrated to prevent 80% of stroke recurrence[8]. However, the rate of adopting healthy behaviors is not encouraging. Liu [9] investigated 515 patients with stroke and found that their health behavior were moderate. In another cross-sectional study[6] involving 112 stroke patients, it was found that only 17% of the participants were adhering to medication regimens. This highlighted the importance of identifying the factors that influence the health behavior of stroke patients and investigating strategies to improve their adherence. Previous studies have shown that various factors, including sex[10], age[11], level of knowledge[12,13], self-efficacy[13,14], recurrence risk perception[15], social support[13,16], and other variables, play a significant role in influencing the health behavior of stroke patients.

Recurrence risk perception represents a critical factor influencing the health behavior[15].In general, recurrence risk perception includes an accurate perception of one's recurrence risk and knowledge of disease recurrence. Actually, there is no clear definition for this variable. Lin[17] defined recurrence risk perception as the perception of stroke patients' early warning symptoms, risk factors, possibility, and severity of recurrence based on the literature review. According to a behavior change study, patients with accurate perceptions of risk were more likely to adhere to preventive measures than those with incorrect perceptions [18]. Moreover, stroke patients who accurately estimated or overestimated the risk of stroke recurrence demonstrated improved non-smoking behavior compared to those who underestimated it[19]. According to the health belief model[20], individuals who hold the belief that engaging in risky behaviors carries significant health risks are more likely to adopt healthier behaviors in an effort to reduce or eliminate future health complications. However, it is worth noting that certain studies have also discovered that patients who perceive a high risk of recurrence may experience heightened levels of anxiety[21,22]. Consequently, the precise relationship between risk perception of recurrence and health behavior remains uncertain. Additional research is warranted to gain further insights and elucidate the connection between these two variables.

Social support is another positive significant factor of health behavior[23]. Social support is defined as the help, support, and resource sharing that people that people receive in the areas of physiology, psychology, emotion, information, and specific situations[20]. Besides, social support is an interactive process that involves providing and receiving social support, which means patients must perceive social support to make social support function[24]. Studies[13,16] have indicated that individuals who perceive encouragement, understanding, and assistance from their social circles are more likely to adopt and sustain healthy behaviors such as exercise, proper diet, and adherence to medical treatments. Perceived social support (PSS) on the other hand is the makeup of all of the social resources that individuals believe are accessible to them[25]. According to the Common-Sense Model of Self-Regulation (CSM)[26], when patients face stress and challenges, they first perceive their physical or emotional reactions and take behaviors to maintain or improve their physical and mental health. The risk of stroke recurrence is a stressful event for patients with stroke. Patients' knowledge and perception of their situation can affect their ability to recognize and utilize available social resources[25]. For patients with stroke, their recurrence risk perceptions affect their abilities to perceive social support around them. Therefore, it is reasonable to assume that there is a relationship between perceived social support and recurrence risk perception. However, limited research exists on the impact of recurrence risk perception on perceived social support and its associated psychological effects among stroke patients. According to the CSM, we speculated the effect of recurrence risk perception on health behavior was partly mediated by perceived social support.

So this study aimed to examine the relationship between the recurrence risk perception and health behavior among patients with stroke and explore the role of perceived social support in this relationship. Notably, there is a dearth of research exploring how recurrence risk perception affects perceived social support and its connection to post-stroke health behavior. It is challenging to find studies that explicitly support the

For peer review only - http://bmjopen.bmj.com/site/about/guidelines.xhtml

mediating impact in this context. To address this gap, we employed a mediational model to assess both the direct and indirect associations. The theoretical framework of the study was constructed by integrating the CSM model, and Figure 1 depicts the relationship among recurrence risk perception, perceived social support, and health behavior.

Insert Figure 1 here.

Methods

Participants and procedure

Convenience sampling was used to conduct this cross-sectional survey from June to August, 2021. Inpatient patients with stroke were selected from the department of neurology at a tertiary hospital in Luoyang City, Henan Province, China. The following were the inclusion requirements for the patients with stroke: inclusion criteria: (a) stroke diagnosed by MRI or CT[27]; (b) during acute stage or stable stage; (c) having the ability to communicate with researchers verbally; (d) over 18-year-old. Exclusion criteria: (a) combined with severe cardiac, liver, and renal dysfunction or other malignant tumors; (b) a history of serious mental illness or a family history of serious mental illness. Kendall[28] proposed that the sample size should be at least 5-10 times the independent variables. There are 16 variables in this study (13 general information questionnaires, the recurrence risk perception of stroke scale, the perceived social support scale, and the health behavior scale for patients with stroke). Considering a loss to follow-up rate of 20%, the minimum sample size of the study was 200 patients with stroke; hence, a sample size of 250 met the requirement for testing the hypothesized models.

Data collection

Inpatients with stroke were recruited for in-person interviews by three trained research assistants. Patients were identified using hospital registries and medical records. Eligibility was confirmed by the primary investigator. Prior to their participation, potential participants received an oral description of the study's purpose and were asked to provide informed consent. As a preliminary step, a survey was conducted before the formal investigation. For this pre-testing phase, ten stroke patients were selected, including five with primary school education and five elderly individuals. The research assistants carefully reviewed the questionnaires completed by these ten patients, identifying any questions that were not well understood (such as those requiring reverse scoring). These questions were clarified to ensure participants' comprehension during the formal investigation.

Measures

The demographic questionnaire

The demographic and disease-specific data questionnaire was designed and used to collect the information of age, sex, marriage, residential area, educational level, working state, stroke type, family history, number of strokes, monthly income, primary caregiver, sleep conditions, and number of chronic diseases.

The stroke recurrence risk perception scale（SRRPS）

The stroke recurrence risk perception scale is a tool used to assess stroke patients' recurrence risk perception of their disease. Lin[17], a member of our research group, developed the scale, which includes two sections with 20 items. The first section contains three items that assess recurrence risk perception of patients with stroke. The second section contains 17 items in three domains: perceived severity, perceived behavior-related risk factors, and perceived disease-related risk factors. There are four items in the “perceived illness risk factors” dimension, six items in the “perceived behavioral risk factors” dimension and seven items in the

For peer review only - http://bmjopen.bmj.com/site/about/guidelines.xhtml

"perceived severity" domain. Higher scores indicate greater risk awareness, and the score for this section is calculated independently of the overall score. The scale has been applied to the stroke population in China. The second section of items ranges from 1-3 (disagree, not clear, agree). The Cronbach's alpha of the second section is 0.850 and the Cronbach's alpha for each dimension are 0.875, 0.815, and 0.804. In this study, we used the second section of the scale to examine stroke patients' perception of recurrence risk.

The Perceived social support scale (PSSS)

The perceived social support scale is an instrument to measure the individual's self-perception of multi-dimensional social support. The scale was developed by Zimet et al. in 1987 and revised into a Chinese version by Jiang[29]in 1999. It includes three dimensions of family support, friend support, and other support (relatives, leaders, colleagues, etc.), with 12 entries. A scale of 1 to 7 was used, with scores from 1 to 7 on a scale from "extremely uncompliant" to "extremely compliant". The total score ranges from 12-84 points, with scores of 12 to 36 points being low perceived support level, 37 to 60 points being middle perceived support level, and 61 to 84 points being high perceived support level. The Cronbach's alpha value of PSSS is 0.92.

The Health behavior scale for stroke patients(HBS-SP)

The health behavior scale for stroke patients is a scale to assess the level of health behavior in patients with stroke. The scale was developed by Wan[30] in 2017. It includes 6 dimensions of exercise, medication taking, instructions, responsibility, nutrition, and smoking and alcohol, with 25 entries. It is a 4-point Likert scale (from 1 = never to 4 = routinely performed). But the items of medication and tobacco, and alcohol were reversed. Higher scores indicate better health behaviors. An average score of 2.5 points (between "sometimes" and "often") is considered an intermediate level of health behavior. The Cronbach's alpha value of HBS-SP scale is 0.878.

Statistical analysis

The Statistical Package of Social Science software version 26 (IBM Corp. Released, 2017) was used to analyze the data. Participants' socio demographics characteristics and other related variables were examined by descriptive statistics of frequencies and percentages. Pearson correlation analysis was used to analyze the interrelationships between the three variables. We applied the testing procedure recommended by Wen[31] and the mediation model was analyzed using Model 4 in the PROCESS Marco[32]. For the best test of the mediation effect, we carried out the bootstrapping procedure to measure the indirect effect and 95% confidence interval (CI) were estimated. $P<0.05$ was considered statistically significant. A 95% confidence interval excluding zero was regarded as statistically significant.

Patient and public involvement

Patients or the public were not involved in the design, conduct, reporting or dissemination of our research.

Results

General Demographics

A total of 254 questionnaires were obtained. However, 4 invalid questionnaires were removed because the questionnaires with a completion rate of less than 95%. Finally, the number of effective participants was 250, and the final effective rate of questionnaire collection was 98.4%. Over half (70.8%) of patients were elderly patients (≥60 years). Less than half of the patients in the sample had a primary school degree or less (46.1%). Moreover, 34.8% were on the job and a majority (97.6%) of which were hemorrhagic stroke. In addition, 50.4% were in the middle monthly income bracket per capita and 52% with a first-time stroke. The participants' demographic characteristics are shown in Supplemental Table .

For peer review only - http://bmjopen.bmj.com/site/about/guidelines.xhtml

Descriptive statistics of the SRRPS, PSSS and HBS-SP

Table 1 displayed mean scores, standard deviations and ranges of SRRPS, PSSS and HBS-SP.

Table 1 Scores on the SRRPS, PSSS and HBS-SP

Variables	Range of score	Mean	Stand deviation
SRRPS	17~51	41.04	8.64
PSSS	40~80	65.86	8.78
HBS-SP	28~81	56.78	9.03

Abbreviations:SRRPS, stroke recurrence risk perception scale; PSSS, Perceived social support scale; HBS-SP, Health behavior scale for stroke patients

Correlation Analysis between SRRPS, PSSS and HBS-SP

Pearson's correlation analysis was used to investigate the relationships among recurrence risk perception, health behavior, and perceived social support in patients with stroke. Briefly, recurrence risk perception was positively correlated with health behavior (r=0.679, P < 0.001), perceived social support was positively correlated with health behavior (r=0.778, P<0.001), recurrence risk perception was positively correlated with perceived social support (r=0.639, P<0.001), meaning that recurrence risk perception, health behavior, and perceived social support were positively correlated with each other in patients with stroke.

Mediation Analysis of SRRPS, PSSS and HBS-SP

Following the correlation analysis results, we performed mediation analysis to further examine the association between perceived social support, recurrence risk perception and health behavior in patients with stroke. We controlled for some general demographic factors (like age, sex, monthly income, education) which are considered as important predictors affect health behavior in patients with stroke in China. In model 1, recurrence risk perception had a positive predictive effect on health behavior (β= 0.607, P < 0.001). In model 2, recurrence risk perception had a positive predictive effect on perceived social support (β= 0.604, P < 0.001). In model 3, recurrence risk perception and perceived social support positively predicted health behavior (β= 0.247, β= 0.596, P < 0.001), but the comparison of the effect coefficients showed that the effect of recurrence risk perception on health behavior was diminished from 0.607 to 0.247, which meant that perceived social support partially mediated the effect between recurrence risk perception and health behavior, as shown in Table 2.

Table 2 Mediation results for recurrence risk perception predicting health behavior mediated by perceived social support

Recurrence risk perception → Health behavior				
	SE	β	t	P
Age	0.968	-1.246	-1.287	0.200
Sex	0.863	0.958	1.110	0.268
Education	0.460	1.187	2.579	0.011
Monthly Income	0.742	1.158	1.561	0.120
Recurrence risk perception	0.053	0.607	11.529	<0.001
Recurrence risk perception →Perceived social support				
Age	0.997	0.240	0.240	0.810
Sex	0.890	0.912	1.026	0.306
Education	0.474	1.669	3.522	0.005
Monthly Income	0.764	-1.514	-1.981	0.049

For peer review only - http://bmjopen.bmj.com/site/about/guidelines.xhtml

Recurrence risk perception	0.054	0.604	11.137	<0.001
Recurrence risk perception, Perceived social support → Health behavior				
Age	0.766	-1.389	-1.812	0.071
Sex	0.685	0.415	0.607	0.545
Education	0.373	0.193	0.516	0.606
Monthly Income	0.592	2.059	3.480	0.006
Recurrence risk perception	0.051	0.247	4.833	<0.001
Perceived social support	0.049	0.596	12.110	<0.001

To ensure the accuracy of the test, this study further used the bootstrap method[32] to test the mediating effect of perceived social support between recurrence risk perception and health behavior with 5000 replicate samples. The results were shown in Table 4, and the 95% confidence interval of the mediating effect of perceived social support was [0.26,0.46], which did not contain 0, indicating that the mediating effect of perceived social support in the process of recurrence risk perception affecting health behavior was established, and the effect accounted for 59.31% (Table 3). The mediating model of perceived social support was constructed in Figure 2.

Table 3 Mediating model examination by bootstrap

Recurrence risk perception → Health behavior				
	Effect	SE	LL 95%CI	UL 95%CI
Indirect effect	0.360	0.050	0.263	0.460
Direct effect	0.247	0.512	0.147	0.348

Insert Figure 2 here.

Discussion

The occurrence of stroke is closely influenced by a variety of factors, including behavioral, psychological, and social environmental factors. Among these factors, the rise in behavioral risk factors plays a significant role in the increasing prevalence of chronic diseases[33]. A recent research study conducted in Estonia also has been demonstrated that young patients who have suffered from a stroke exhibited significantly poorer health behavior prior to the event compared to the general population[34]. Indeed, the adoption of healthy behaviors has long been recognized as a crucial factor in preventing stroke recurrence. By actively engaging in positive lifestyle choices, individuals can significantly reduce their risk of experiencing another stroke. These behaviors serve as important protective factors for both primary prevention (preventing the initial stroke) and secondary prevention (preventing recurrent strokes) among stroke patients. Although the promotion of health behavior has deserved continuous attention, the adoption of health behavior in patients with stroke was not optimistic globally. A cross-sectional study carried out in Albania showed that about 32% of the brain patients with stroke manifested unhealthy behavior[35]. Another study conducted in Korea also found that patients with stroke had low health promotion behaviors. Also, some studies have found patients with stroke have moderate healthy behavior in China[9][36], which was certified again in our study. We found that 78.4% of patients with stroke scored less than 2.5 on the health behavior scale, which meant they had moderately low health behavior. This highlighted the urgent need for further research and focus on understanding the barriers that prevent stroke patients from adopting healthy behaviors.

For peer review only - http://bmjopen.bmj.com/site/about/guidelines.xhtml

This study demonstrated that perceived social support was positively correlated with health behavior. This finding was consistent with past researches[37,38]. A cross-sectional study[37]based on data from the Spanish National Health Survey (ENSE2017) with a final sample of 1006 individuals with diabetes mellitus has shown the perceived social support was positively correlated with physical activity. The same results were confirmed in another study. Guo[38] conducted a community-based cross-sectional study among 1697 participants with hypertension in China and found social support had a direct positively effect on medication adherence. Moreover, Bandura's social theory[39] also proposed that factors such as social support were actual aspects of the incidence of behavior. Therefore, perceived social support is an important factor in improving health behavior in patients with stroke. In addition, there was a significant positive correlation between recurrence risk perception and perceived social support. In fact, in the traditional Chinese mindset, "recurrence" and "risk" are often feared[40]. Fear is a negative emotion that is commonly experienced by patients with stroke, leading them to seek escape or isolate themselves from their surroundings[41], which can lead to a decrease in the perceived level of social support for patients with stroke. As a result, our initial hypothesis was contradicted by the findings of our study. In other words, we were surprised by the results of our study. We speculated the reason for this result was that the higher stroke patients' recurrence risk perception, the more control they had over their diseases. This sense of control over the disease could partly increase their self-efficacy[42] to stay in a good mind, promoting a positive mindset, and concurrently enhancing their perception of social support. Calderon[43] discovered that a majority of chronic patients expressed a desire for increased information and greater involvement in the decision-making process. To the best of our knowledge, our study was the first to investigate the association between stroke patients' perceptions of recurrence and their perceived social support in China.

Most importantly, we found that recurrence risk perception appeared to influence health behavior via two pathways, a direct pathway and an indirect pathway mediated by perceived social support. (1) First pathway, recurrence risk perception was correlated with health behavior significantly and positively. During our literature review, we found that there is no unanimous conclusion about this relationship[19,21,22,44–46]. Some studies have found that patients with a higher risk perception of recurrence had more serious concerns about their disease symptoms[41], and even some patients were overly vigilant about disease symptoms[21,22]. As a result, a higher risk perception of recurrence was not conducive for them to adopt healthy behaviors. Conversely, other studies have confirmed that patients who lacked a perception of the risk of recurrence and preventive strategies exhibited a tendency to limit their regular hospital visits or refrain from adopting healthy behaviors aimed at preventing recurrence[19,40]. In other words, recurrence risk perception was an important positive factor of health behavior. Our finding supported the second category of studies. The reasons for this situation can be explained by the health belief model (HBM). According to the HBM, patients' health behaviors are closely linked to their perception of disease risk and patients are more likely to adopt preventive health behavior when they believe there is a possibility of disease recurrence[46]. (2) The second pathway was that recurrence risk perception mediated the effect of perceived social support on health behavior. To effectively implement secondary stroke prevention measures, it is crucial for patients with stroke to have a comprehensive understanding of the risk factors associated with recurrent stroke. Equally important is their awareness of the lifestyle modifications required to minimize the risk of future recurrences[47]. In patients with stroke, the perceived risk of recurrence encompasses more than just concern and fear. It also involves the patients' understanding of early warning signs and risk factors associated with stroke recurrence. So patients with stroke with a higher recurrence risk perception also demonstrated a greater depth of stroke knowledge[48], and these patients showed a higher level of confidence in recovery than patients with a lower perceived risk of recurrence. Therefore, they were more able to perceive information or emotional support from those around them. The presence of these social supports played a significant role in improving their emotional well-being, consequently fostering a greater willingness to embrace healthier behaviors.

Overall, our study confirmed that recurrence risk perception not only directly and positively influenced

For peer review only - http://bmjopen.bmj.com/site/about/guidelines.xhtml

health behaviors of patients with stroke, but also further influenced health behavior by positively affecting perceived social support. This was consistent with the connotation of the CSM model. In other words, our findings provided practical support for the CSM model. As the practical implication is that our finding may aid future research in identifying entry points for health behavior interventions by taking the methods that improve stroke patients' perception of recurrence risk and perceived social support. Therefore, healthcare professionals can educate patients with stroke on recurrence risk communication. Specifically, they are supposed to assist patients in developing an accurate perception of recurrence risk, as well as equipping them with the necessary knowledge and skills to prevent a recurrence. By doing so, healthcare professionals can enhance stroke patients' confidence in their recovery, leading to improved perception of social support and ultimately facilitating the adoption of healthy behaviors.

The study still has a few limitations. Firstly, it employed a cross-sectional design, thereby precluding the establishment of a causal relationship between recurrence risk perception and health behavior. Nonetheless, the study successfully confirmed a significant correlation between them. Secondly, despite our efforts to incorporate multi-center sampling, the impact of COVID-19 necessitated the restriction of our study to a single tertiary hospital in Henan Province. Therefore, the findings may not be generalizable to all patients with stroke. Lastly, it is important to note that the patients with stroke included in this study spanned across all age groups. While this was done to ensure representativeness, it should be acknowledged that variables such as health behaviors may vary among patients with stroke of different age brackets. Therefore, for future research, we recommend the design of a multi-center longitudinal study specifically focusing on young stroke patients. Such a study would help clarify the dynamic trends in recurrence risk perception, perceived social support, and health behaviors over time.

Conclusion

This study broadened the research on the relationships between recurrence risk perception, perceived social support and health behavior in patients with stroke. The findings of this study can serve as a valuable reference for future clinical nursing care and research in this field. We confirmed that perceived social support mediated the relationship between recurrence risk perception and health behavior. Recurrence risk perception and perceived social support should be included in the assessment process of patients with stroke at hospitalization or follow-up in the future. We recommend that healthcare professionals assist patients with stroke in developing an accurate perception of recurrence risk through the use of appropriate recurrence risk communication approaches. This approach can effectively enhance patients' ability to recognize and seek social support, thereby boosting their confidence in recovery and motivating them to adopt healthier behaviors. In addition, social support as a fundamental source of perceived social support should not be ignored. Healthcare professionals should encourage stroke patients' families and friends to provide them with more emotional support.

For peer review only - http://bmjopen.bmj.com/site/about/guidelines.xhtml

Acknowledgements

We would like to acknowledge the tertiary hospital of [redacted] for their cooperation.We greatly appreciate the support of all patients for their voluntary participation in this study.

Contributors

ZZX and LBL designed the study. WXX and WWN contributed to writing and revising. WXX, MYX and ZCH were in charge of data collection and analysis. ZQS and CSY were in charge of study design and essential help. All authors read and approved the final manuscript.

Funding

This work was supported by the National Natural Science Foundation of China Youth Science Foundation Project [redacted]

Competing interests

None declared.

Patient consent for publication

Each participant gave informed written consent and permission to publish before analysis of the study.

Ethics approval

The Research Ethics Committee of [redacted] approved the study.

Provenance and peer review

Not commissioned; externally peer reviewed.

Data availability statement

Data are available upon reasonable request.

For peer review only

For peer review only - http://bmjopen.bmj.com/site/about/guidelines.xhtml

References

1 Lin B, Zhang Z, Mei Y, *et al.* Cumulative risk of stroke recurrence over the last 10 years: a systematic review and meta-analysis. *Neurol Sci* 2021;**42**:61–71. doi:10.1007/s10072-020-04797-5

2 Gladstone DJ, Lindsay MP, Douketis J, *et al.* Canadian Stroke Best Practice Recommendations: Secondary Prevention of Stroke Update 2020. *Can J Neurol Sci* 2022;**49**:315–37. doi:10.1017/cjn.2021.127

3 Kleindorfer DO, Towfighi A, Chaturvedi S, *et al.* 2021 Guideline for the Prevention of Stroke in Patients With Stroke and Transient Ischemic Attack: A Guideline From the American Heart Association/American Stroke Association. *Stroke* 2021;**52**:e364–467. doi:10.1161/STR.0000000000000375

4 Liu L, Chen W, Zhou H, *et al.* Chinese Stroke Association guidelines for clinical management of cerebrovascular disorders: executive summary and 2019 update of clinical management of ischaemic cerebrovascular diseases. *Stroke Vasc Neurol* 2020;**5**:159–76. doi:10.1136/svn-2020-000378

5 Tsao CW, Aday AW, Almarzooq ZI, *et al.* Heart Disease and Stroke Statistics—2022 Update: A Report From the American Heart Association. *Circulation* 2022;**145**:e153–639. doi:10.1161/CIR.0000000000001052

6 Mugwano I, Kaddumukasa M, Mugenyi L, *et al.* Poor drug adherence and lack of awareness of hypertension among hypertensive stroke patients in Kampala, Uganda: a cross sectional study. *BMC Res Notes* 2016;**9**:3. doi:10.1186/s13104-015-1830-4

7 Bawand R, Ghiasian M, Khazaei M, *et al.* Effects of blood pressure unawareness and poor adherence to antihypertensive drugs on outcomes of cerebrovascular accidents among patients with their first-ever stroke. *J Hypertens* 2023;**41**:459–69. doi:10.1097/HJH.0000000000003358

8 Abdullahi SU, Sunusi S, Abba MS, *et al.* Hydroxyurea for secondary stroke prevention in children with sickle cell anemia in Nigeria: a randomized controlled trial. *Blood* 2023;**141**:825–34. doi:10.1182/blood.2022016620

9 Liu Y, Wei M, Guo L, *et al.* Association between illness perception and health behaviour among stroke patients: The mediation effect of coping style. *Journal of Advanced Nursing* 2021;**77**:2307–18. doi:10.1111/jan.14761

10 Wu X, Min L, Cong L, *et al.* Sex differences in health-related quality of life among adult stroke patients in Northeastern China. *J Clin Neurosci* 2014;**21**:957–61. doi:10.1016/j.jocn.2013.08.030

11 Kim G-G, Chae D-H, Park M-S, *et al.* Factors Influencing 1-Year Medication Adherence of Korean Ischemic Stroke Survivors. *Int J Behav Med* 2020;**27**:225–34. doi:10.1007/s12529-020-09854-z

12 Parappilly BP, Field TS, Mortenson WB, *et al.* Determinants Influencing the Prestroke Health Behaviors and Cardiovascular Disease Risk of Stroke Patients: A Cross-Sectional Study. *J Stroke Cerebrovasc Dis* 2019;**28**:1509–18. doi:10.1016/j.jstrokecerebrovasdis.2019.03.015

13 Santos M, Kövari E, Gold G, *et al.* The neuroanatomical model of post-stroke depression: towards a change of focus? *J Neurol Sci* 2009;**283**:158–62. doi:10.1016/j.jns.2009.02.334

For peer review only - http://bmjopen.bmj.com/site/about/guidelines.xhtml

14 Brouwer-Goossensen D, Genugten L van, Lingsma H, *et al.* Determinants of intention to change health-related behavior and actual change in patients with TIA or minor ischemic stroke. *Patient Educ Couns* 2016;**99**:644–50. doi:10.1016/j.pec.2015.10.028

15 Ren H, Guo Y-F, Zhang Z-X, *et al.* Perception of recurrent risk versus objective measured risk of ischemic stroke in first-ever stroke patients from a rural area in China: A cross-sectional study. *Patient Educ Couns* 2023;**107**:107586. doi:10.1016/j.pec.2022.107586

16 Holder C, Krishnamurthi R, Theadom A. Exploring facilitators and barriers to long-term behavior change following health-wellness coaching for stroke prevention: A qualitative study conducted in Auckland, New Zealand. *Brain Behav* 2023;**13**:e2671. doi:10.1002/brb3.2671

17 Lin B, Zhang Z, Guo Y. Development and psychometric test of Recurrence Risk Perception Scale for Patients with Stroke. *Chinese Journal of Nursing* 2022;**56**:1666–71.

18 Kreuter MW, Strecher VJ. Changing inaccurate perceptions of health risk: results from a randomized trial. *Health Psychol* 1995;**14**:56–63. doi:10.1037//0278-6133.14.1.56

19 Li J-J, Tan J-X, Zhu F-Y, *et al.* Comparisons of Stroke Knowledge and Health Behaviors in Patients With Hypertensive Stroke at Different Recurrence Risk Strata: The Comprehensive Reminder System Based on the Health Belief Model Study. *J Cardiovasc Nurs* 2022;**37**:184–91. doi:10.1097/JCN.0000000000000765

20 Champion VL, Skinner CS. The health belief model. In: *Health behavior and health education: Theory, research, and practice, 4th ed.* San Francisco, CA, US: : Jossey-Bass 2008. 45–65.

21 Freeman-Gibb LA, Janz NK, Katapodi MC, *et al.* The relationship between illness representations, risk perception and fear of cancer recurrence in breast cancer survivors. *Psychooncology* 2017;**26**:1270–7. doi:10.1002/pon.4143

22 Lee-Jones C, Humphris G, Dixon R, *et al.* Fear of cancer recurrence--a literature review and proposed cognitive formulation to explain exacerbation of recurrence fears. *Psychooncology* 1997;**6**:95–105. doi:10.1002/(SICI)1099-1611(199706)6:2<95::AID-PON250>3.0.CO;2-B

23 Blanton S, Scheibe DC, Rutledge AH, *et al.* Family-Centered Care During Constraint-Induced Therapy After Chronic Stroke: A Feasibility Study. *Rehabil Nurs* 2019;**44**:349–57. doi:10.1097/rnj.0000000000000197

24 Northcott S, Moss B, Harrison K, *et al.* A systematic review of the impact of stroke on social support and social networks: associated factors and patterns of change. *Clin Rehabil* 2016;**30**:811–31. doi:10.1177/0269215515602136

25 Dahlan R, Ghazal E, Saltaji H, *et al.* Impact of social support on oral health among immigrants and ethnic minorities: A systematic review. *PLoS One* 2019;**14**:e0218678. doi:10.1371/journal.pone.0218678

26 Leventhal H, Phillips LA, Burns E. The Common-Sense Model of Self-Regulation (CSM): a dynamic framework for understanding illness self-management. *J Behav Med* 2016;**39**:935–46. doi:10.1007/s10865-016-9782-2

27 Neurology CS of, Society CS. Diagnostic criteria of cerebrovascular diseases in China (version 2019).

For peer review only - http://bmjopen.bmj.com/site/about/guidelines.xhtml

Chinese Journal of Neurology 2019;**52**:710–5. doi:10.3760/cma.j.issn.1006-7876.2019.09.003

28 Ni P, Chen J, Liu N. Sample size estimation in quantitative nursing research. *Chinese Journal of Nursing* 2010;**45**:378–80.

29 Wang X, Wang X, Ma H. *Manual of the Mental Health Rating Scale*. Beijing: : Chinese Journal of Mental Health 1999.

30 Wan L, Xiong X, Pan J, *et al.* Development and reliability as well as validity test of Health Behavior Scale for stroke patients. *Journal of Nursing Science* 2017;**32**:25–9.

31 Wen Z. Analyses of Mediating Effects: The Development of Methods and Models. *Advances in psychological Science* 2014;**22**:731–45.

32 Henderson AR. The bootstrap: a technique for data-driven statistics. Using computer-intensive analyses to explore experimental data. *Clin Chim Acta* 2005;**359**:1–26. doi:10.1016/j.cccn.2005.04.002

33 O'Donnell MJ, Xavier D, Liu L, *et al.* Risk factors for ischaemic and intracerebral haemorrhagic stroke in 22 countries (the INTERSTROKE study): a case-control study. *Lancet* 2010;**376**:112–23. doi:10.1016/S0140-6736(10)60834-3

34 Saapar M, Vibo R, Schneider S, *et al.* Health behavior of young patients with ischemic stroke in Estonia: A score of five factors. *Brain Behav* 2023;**13**:e2908. doi:10.1002/brb3.2908

35 Lalo R, Zekja I, Kamberi F. Association of Cardiovascular Disease Risk and Health-Related Behaviors in Stroke Patients. *Int J Environ Res Public Health* 2023;**20**:3693. doi:10.3390/ijerph20043693

36 Zhang J, Gong Y, Zhao Y, *et al.* Post-stroke medication adherence and persistence rates: a meta-analysis of observational studies. *J Neurol* 2021;**268**:2090–8. doi:10.1007/s00415-019-09660-y

37 Denche-Zamorano A, Pisà-Canyelles J, Barrios-Fernandez S, *et al.* Is Psychological Distress Associated with Self-Perceived Health, Perceived Social Support and Physical Activity Level in Spanish Adults with Diabetes? *J Pers Med* 2023;**13**:739. doi:10.3390/jpm13050739

38 Guo A, Jin H, Mao J, *et al.* Impact of health literacy and social support on medication adherence in patients with hypertension: a cross-sectional community-based study. *BMC Cardiovasc Disord* 2023;**23**:93. doi:10.1186/s12872-023-03117-x

39 Bandura A. Social cognitive theory: an agentic perspective. *Annu Rev Psychol* 2001;**52**:1–26. doi:10.1146/annurev.psych.52.1.1

40 Lin B, Zhang Z, Guo Y, *et al.* Perceptions of recurrence risk and behavioural changes among first-ever and recurrent stroke survivors: A qualitative analysis. *Health Expect* 2021;**24**:1962–70. doi:10.1111/hex.13335

41 Singh T, Newby DE. Is the fear of disease worse than the disease itself? *Heart* 2021;**107**:91–2. doi:10.1136/heartjnl-2020-318207

42 Al Shafaee MA, Ganguly SS, Al Asmi AR. Perception of stroke and knowledge of potential risk factors among Omani patients at increased risk for stroke. *BMC Neurol* 2006;**6**:38. doi:10.1186/1471-2377-6-38

For peer review only

43 Calderon C, Jiménez-Fonseca P, Ferrando PJ, *et al.* Psychometric properties of the Shared Decision-Making Questionnaire (SDM-Q-9) in oncology practice. *Int J Clin Health Psychol* 2018;**18**:143–51. doi:10.1016/j.ijchp.2017.12.001

44 Gibson TM, Li C, Armstrong GT, *et al.* Perceptions of future health and cancer risk in adult survivors of childhood cancer: A report from the Childhood Cancer Survivor Study. *Cancer* 2018;**124**:3436–44. doi:10.1002/cncr.31397

45 Liu Y, Pérez M, Schootman M, *et al.* Correlates of fear of cancer recurrence in women with ductal carcinoma in situ and early invasive breast cancer. *Breast Cancer Res Treat* 2011;**130**:165–73. doi:10.1007/s10549-011-1551-x

46 Melkamu L, Berhe R, Handebo S. Does Patients' Perception Affect Self-Care Practices? The Perspective of Health Belief Model. *Diabetes Metab Syndr Obes* 2021;**14**:2145–54. doi:10.2147/DMSO.S306752

47 Hiraga A. Perception of recurrent stroke risk among stroke survivors. *Neuroepidemiology* 2011;**37**:88–9. doi:10.1159/000330354

48 Slark J, Bentley P, Majeed A, *et al.* Awareness of stroke symptomatology and cardiovascular risk factors amongst stroke survivors. *J Stroke Cerebrovasc Dis* 2012;**21**:358–62. doi:10.1016/j.jstrokecerebrovasdis.2010.09.010

For peer review only

For peer review only - http://bmjopen.bmj.com/site/about/guidelines.xhtml

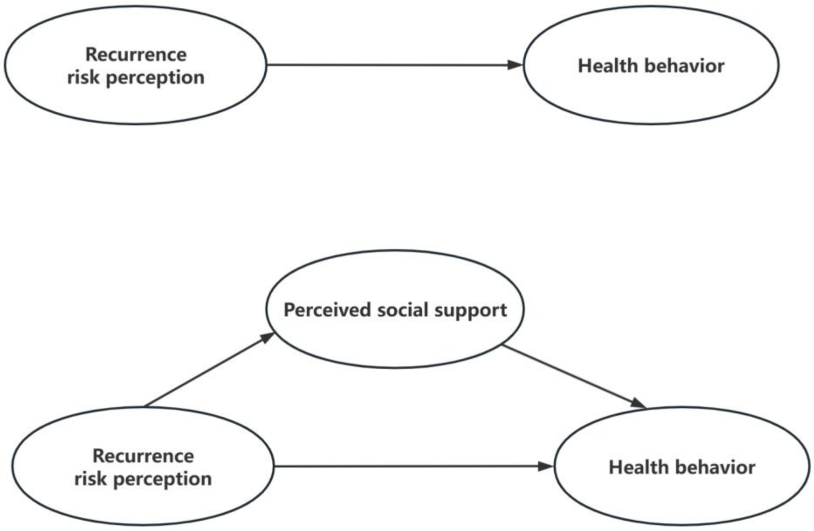

Figure 1 The hypothesis model of recurrence risk perception, health behavior and perceived social support in patients with stroke

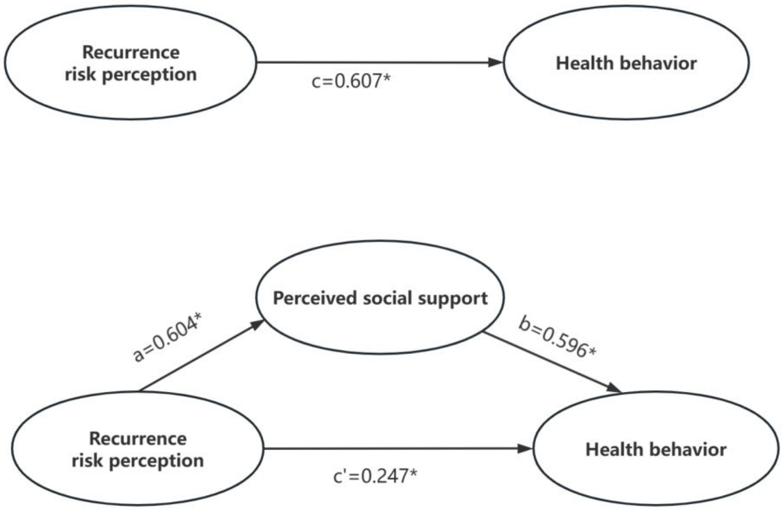

Figure 2 Mediating effects of perceived social support on the relationship between recurrence risk perception and health behavior (*P < 0.001)

Supplemental Table Participants' sociodemographic characteristics

Factors	Group	*N*	%
Sex	Male	136	54.4
	Female	114	45.6
Marriage	Married	220	88
	Divorced	2	0.8
	Unmarried	28	11.2
Age	＜60	73	29.2
	≥60	177	70.8
Residential area	Rural	126	50.4
	Urban	124	49.6
Working state	Unemployed	54	21.6
	Retire/leave	109	43.6
	On-the-job	87	34.8
Educational level	Primary and below	115	46
	Junior high school	65	27.6
	High school	44	17.6
	College	14	5.6
	University and above	12	4.8
Stroke type	Ischemic stroke	6	2.4
	Hemorrhagic stroke	244	97.6
Family history	Have	25	10
	No	255	90
Monthly income (RMB)	≤2999	89	35.6
	3000-4999	126	50.4
	≥5000	35	14
Numbers of stroke	1 time	130	52
	2 times	74	29.6
	3 times	22	8.8
	More than 3 times	24	9.6
Primary caregiver	Spouse or children	132	52.8
	Babysitter or nursing workers	11	4.4
	Own	107	42.8
Number of chronic diseases	0	75	30
	1~2	150	60
	3 and above	25	10
Sleep conditions	Very good	92	36.8

	Good	69	27.6
	Poor	69	27.6
	Very poor	20	8

For peer review only

STROBE 2007 (v4) checklist of items to be included in reports of observational studies in epidemiology*

Checklist for cohort, case-control, and cross-sectional studies (combined)

	Item No	Recommendation	Page
**Title and abstract**	1	(*a*) Indicate the study's design with a commonly used term in the title or the abstract	Page 1
		(*b*) Provide in the abstract an informative and balanced summary of what was done and what was found	Page 1
**Introduction**			
Background/rationale	2	Explain the scientific background and rationale for the investigation being reported	Page 2-4
Objectives	3	State specific objectives, including any prespecified hypotheses	Page 5
**Methods**			
Study design	4	Present key elements of study design early in the paper	Page 5
Setting	5	Describe the setting, locations, and relevant dates, including periods of recruitment, exposure, follow-up, and data collection	Page 5、Page 6
Participants	6	(*a*) Give the eligibility criteria, and the sources and methods of selection of participants	Page 6
Variables	7	Clearly define all outcomes, exposures, predictors, potential confounders, and effect modifiers. Give diagnostic criteria, if applicable	Page 7-8
Data sources/ measurement	8*	For each variable of interest, give sources of data and details of methods of assessment (measurement). Describe comparability of assessment methods if there is more than one group	Page 7-8
Bias	9	Describe any efforts to address potential sources of bias	Page 7、10
Study size	10	Explain how the study size was arrived at	Page 6
Quantitative variables	11	Explain how quantitative variables were handled in the analyses. If applicable, describe which groupings were chosen and why	Page 7
Statistical methods	12	(*a*) Describe all statistical methods, including those used to control for confounding	Page 7
		(*b*) Describe any methods used to examine subgroups and interactions	Not applicable
		(*c*) Explain how missing data were addressed	Page 7
		(*d*) If applicable, describe analytical methods taking account of sampling strategy	Not applicable
		(*e*) Describe any sensitivity analyses	Not applicable
**Results**			

For peer review only

Participants	13*	(a) Report numbers of individuals at each stage of study—eg numbers potentially eligible, examined for eligibility, confirmed eligible, included in the study, completing follow-up, and analysed	Not applicable
		(b) Give reasons for non-participation at each stage	Not applicable
		(c) Consider use of a flow diagram	Not applicable
Descriptive data	14*	(a) Give characteristics of study participants (eg demographic, clinical, social) and information on exposures and potential confounders	Page 7
		(b) Indicate number of participants with missing data for each variable of interest	Page 7
Outcome data	15*	Report numbers of outcome events or summary measures	Page 7
Main results	16	(*a*) Give unadjusted estimates and, if applicable, confounder-adjusted estimates and their precision (eg, 95% confidence interval). Make clear which confounders were adjusted for and why they were included	Page 8
		(*b*) Report category boundaries when continuous variables were categorized	Page 6
		(*c*) If relevant, consider translating estimates of relative risk into absolute risk for a meaningful time period	Not applicable
Other analyses	17	Report other analyses done—eg analyses of subgroups and interactions, and sensitivity analyses	Not applicable

For peer review only

**Discussion**			
Key results	18	Summarise key results with reference to study objectives	Page 9
Limitations	19	Discuss limitations of the study, taking into account sources of potential bias or imprecision. Discuss both direction and magnitude of any potential bias	Page 11
Interpretation	20	Give a cautious overall interpretation of results considering objectives, limitations, multiplicity of analyses, results from similar studies, and other relevant evidence	Page 9-10
Generalisability	21	Discuss the generalisability (external validity) of the study results	Page 11
**Other information**			
Funding	22	Give the source of funding and the role of the funders for the present study and, if applicable, for the original study on which the present article is based	Page 12

*Give information separately for exposed and unexposed groups.

For peer review only

Mediation role of perceived social support between recurrence risk perception and health behavior among patients with stroke in China: a cross-sectional study

Journal:	*BMJ Open*
Manuscript ID	bmjopen-2023-079812.R1
Article Type:	Original research
Date Submitted by the Author:	10-Nov-2023
Complete List of Authors:	Wang, Xiaoxuan; Zhengzhou University,<br>Zhang, Zhen-xiang; Zhengzhou University,<br>LIN, Bei-lei; Zhengzhou University<br>Jiang, Hu; Zhengzhou University, ;<br>Wang, Wenna; Zhengzhou University,<br>MEI, YONG-XIA; Zhengzhou University, School of Nursing and health<br>Zhang, Chunhui; Nursing and Health school, Zhengzhou University<br>Zhang, Qiushi; Zhengzhou University, Department of Fundmental Nursing<br>CHEN, Su-yan; Zhengzhou University
**Primary Subject Heading**:	Nursing
Secondary Subject Heading:	Nursing
Keywords:	Stroke < NEUROLOGY, Behavior, Chronic Disease

I, the Submitting Author has the right to grant and does grant on behalf of all authors of the Work (as defined in the below author licence), an exclusive licence and/or a non-exclusive licence for contributions from authors who are: i) UK Crown employees; ii) where BMJ has agreed a CC-BY licence shall apply, and/or iii) in accordance with the terms applicable for US Federal Government officers or employees acting as part of their official duties; on a worldwide, perpetual, irrevocable, royalty-free basis to BMJ Publishing Group Ltd ("BMJ") its licensees and where the relevant Journal is co-owned by BMJ to the co-owners of the Journal, to publish the Work in this journal and any other BMJ products and to exploit all rights, as set out in our licence.

The Submitting Author accepts and understands that any supply made under these terms is made by BMJ to the Submitting Author unless you are acting as an employee on behalf of your employer or a postgraduate student of an affiliated institution which is paying any applicable article publishing charge ("APC") for Open Access articles. Where the Submitting Author wishes to make the Work available on an Open Access basis (and intends to pay the relevant APC), the terms of reuse of such Open Access shall be governed by a Creative Commons licence – details of these licences and which Creative Commons licence will apply to this Work are set out in our licence referred to above.

Other than as permitted in any relevant BMJ Author's Self Archiving Policies, I confirm this Work has not been accepted for publication elsewhere, is not being considered for publication elsewhere and does not duplicate material already published. I confirm all authors consent to publication of this Work and authorise the granting of this licence.

For peer review only

Mediation role of perceived social support between recurrence risk perception and health behavior among patients with stroke in China: a cross-sectional study

Abstract

Objectives To examine whether stroke patients with high recurrence risk perception would have healthier behavior, and to explore whether perceived social support would function as a mediator.

Design A cross-sectional study.

Setting The study was conducted in a public tertiary hospital in China.

Participants A total of 254 patients with stroke were invited to participate, and 250 patients with stroke completed questionnaires validly.

Primary and secondary outcome measures Questionnaires were administered offline to collect data, consisting of 4 parts: general demographics, and scales related to recurrence risk perception, perceived social support and health behavior. A path analysis and correlation analysis were used to analyse the data.

Results Out of 250 patients with stroke, 78.4% were below the medium level. The majority (70.8%) of these patients were elderly. High recurrence risk perception and high perceived social support were significantly associated with better health behavior (all P<0.001). Perceived social support mediated the relationship between recurrence risk perception and health behavior, after controlling for age, gender, education, monthly income in the regression models (95% CI=[0.263,0.460]). It was also confirmed that perceived social support had a highest mediation effect with a proportion of mediation up to 59.31%.

Conclusions Recurrence risk perception and perceived social support were influential factors in promoting health behavior. Moreover, the impact of recurrence risk perception on health behavior was partially mediated by perceived social support. Therefore, to enhance the sustainability of health behavior, it is crucial to inform patients with stroke about the risk of recurrence. Patients with more perception of recurrence risk can improve their recovery confidence and thus perceive more social support.

Strengths and limitations of this study

- A path analysis is used to analyse the associations among perceived social support, recurrence risk perception and health behavior.
- This study is a cross-sectional survey and therefore cannot make causal inferences.
- Survey data from only one region may limit the generalizability of our findings.

Keywords recurrence risk perception, health behavior, perceived social support, mediation effect, nursing, stroke

Introduction

Globally, stroke recurrence is a significant but preventable risk. A study which contained 500,000 Chinese adults showed that the cumulative recurrence rate for stroke patients was 17% at 1 year, 41% at 5 years, and 53% at 9 years [1]. Despite the implementation of policies and guidelines in various countries aimed at preventing secondary strokes, there has been no significant decrease in the rates of stroke recurrence [2–4]. The occurrence of stroke is closely influenced by a variety of factors, including behavioral, psychological, and social environmental factors. Among these factors, behavioral risk factors plays a significant role in the increasing prevalence of chronic diseases [5].

Adopting healthy lifestyle habits can significantly reduce the risk of stroke recurrence. Specifically, engaging in regular physical activity, following a balanced diet, quitting smoking, and consuming alcohol in

For peer review only - http://bmjopen.bmj.com/site/about/guidelines.xhtml

moderation have been shown to collectively prevent approximately 80% of stroke recurrences [6]. Despite the proven benefits of healthy behavior adoption, the actual rate of implementation remains discouragingly low. Liu et al [7] investigated 515 patients with stroke and found that health behavior level of patients were moderate. In another cross-sectional study[8] involving 112 stroke patients, it was found that only 17% of the participants were adhering to medication regimens. This highlighted the importance of identifying the factors that influence the health behavior of stroke patients and investigating strategies to improve their adherence. Previous studies have shown that various factors, including gender [9], age [10], level of knowledge [11,12], self-efficacy [12,13], recurrence risk perception [14] and social support [12,15] play a significant role in influencing the health behavior of stroke patients.

Recurrence risk perception represents a critical factor influencing the health behavior [14]. Recurrence risk perception refers to the perception of early warning features of recurrence, severity, behavior-related risk factors, and disease-related risk factors [16]. The study concluded that stroke patients' perception of recurrence risk was an important predictor for patients to adopt healthy behaviors to control and reduce the recurrence of stroke [17]. However, the precise relationship between risk perception of recurrence and health behavior actually remains uncertain. According to the health belief model [18], individuals who hold the belief that engaging in risky behaviors carries significant health risks are more likely to adopt healthier behaviors. In other words, recurrence risk perception affects health behavior positively. On the contrary, a cross-sectional comparative study conducted in China showed that patients with stroke who perceived higher risk of recurrence were more likely to adopt healthy behavior [14]. Iversen et al [19] investigated 435 patients with stroke and confirmed that higher seriousness perception of stroke was associated with more help-seeking behavior and shorter prehospital delay. There is also another argument that recurrence risk perception is detrimental to health behavior. Freeman-Gibb et al [20] recruited 117 patients and found patients who perceive a high risk of recurrence always experience higher levels of anxiety and fear. Excessive anxiety and fear were actually detrimental to the patient's adoption of health behavior [21]. Consequently, in order to understand how stroke risk perception affects the likelihood of people adopting healthy behaviors, more research is needed.

Social support is another significant factor of health behavior [22]. It is an interactive process that involves providing and receiving social support, which means social support must be perceived by patients so that it can be effective [23]. Studies [12,15] have indicated that individuals who perceive encouragement, understanding, and assistance from their social circles are more likely to adopt and sustain healthy behaviors such as exercise, proper diet, and adherence to medical treatments.

Perceived social support (PSS) refers to an individual's belief in the availability of various social resources, including emotional support, tangible aid, and informational assistance [24]. The Common Sense Model of self-regulation (CSM) is a theoretical framework which is widely used in the study of illness perception [25]. It was designed to describe dynamic interactions among the variables controlling health behavior in response to current or future health threats [26]. The CSM comprises six main components [29]: (1) situational stimuli, (2) cognitive illness representations, (3) emotional illness representations, (4) coping strategies, (5) illness and emotional outcomes, and (6) coping appraisal. Previous studies have already applied the CSM model to predict health outcomes in diabetes [27], explore the experience of breathlessness [28] and predict healthy eating among individuals at risk of metabolic syndrome [29]. For patients with stroke, the surrounding situational stimuli (including endogenous or social factors) cause patients with stroke to develop cognitive and emotional illness representation (recurrence risk perception). Cognitive and emotional illness representation are also continually updated [29] in response to memories of new information (e.g., perceived social support), which affects coping strategies (the choice of adopting health behavior). Therefore, it is reasonable to choose the CSM model as the theoretical framework for our study.

According to the CSM, we speculated the effect of recurrence risk perception on health behavior was partly mediated by perceived social support. Nevertheless, there is a significant gap in the knowledge regarding the impact of an individual's perception of stroke recurrence risk on their sense of social support

For peer review only - http://bmjopen.bmj.com/site/about/guidelines.xhtml

and subsequent emotional consequence [30]. In fact, in traditional Chinese philosophy, the concepts of 'recurrence' and 'risk' are often met with fear and apprehension. Stroke patients may experience avoidance or isolation behavior as a result of their fear, subsequently resulting in a decline in their perceived social support networks [31], ultimately reducing the perceived level of social support for stroke patients. Therefore, we hypothesized that recurrence risk perception affects the perceived social support directly and negatively. Moreover, patients with higher recurrence risk perception may be prevented from adopting healthy behavior due to the negative emotions. It proposes the following hypothesis (Figure 1). This study aimed to examine the relationship between the recurrence risk perception and health behavior among patients with stroke and explore the role of perceived social support in this relationship.

Hypothesis 1 – Recurrence risk perception affects health behavior directly and negatively.
Hypothesis 2 – Recurrence risk perception affects the perceived social support directly and negatively.
Hypothesis 3 – Perceived social support affects health behavior directly and positively.
Hypothesis 4– Recurrence risk perception indirectly affects health behavior via Perceived social support

Insert Figure 1 here.

Methods

Participants and procedure

Convenience sampling was used to conduct this cross-sectional survey from June to August, 2021. Inpatient patients with stroke were selected from the department of neurology at a tertiary hospital in Luoyang City, Henan Province, China. The following were the inclusion criteria: (a) patients were diagnosed with stroke by MRI or CT [32]; (b) during recovery stage (2 weeks to 6 months after onset [33]); (c) having the ability to communicate with researchers verbally; (d) over 18-year-old. Exclusion criteria: (a) combined with severe cardiac, liver, and renal dysfunction or other malignant tumors; (b) a history of serious mental illness or a family history of serious mental illness. Kendall [34] proposed that the sample size should be at least 5-10 times the independent variables. There were 16 variables in this study (13 general information questions, the recurrence risk perception of stroke scale, the perceived social support scale, and the health behavior scale for patients with stroke). Considering 20% of invalid questionnaires, the minimum sample size of the study was 200 patients with stroke; hence, a sample size of 250 met the requirement for testing the hypothesized models.

Data collection

To ensure the validity and reliability of our study, we first conducted a pre-test with a small sample of stroke patients. We selected five patients with primary school education and five elderly individuals to participate in this pre-test phase. Our research assistants carefully reviewed the questionnaires completed by these participants and identified any questions that were unclear or required further explanation. We then made necessary revisions to ensure that all participants would understand the questions accurately during the formal investigation.

Measures

The demographic questionnaire

The demographic and disease-specific data questionnaire was designed and used to collect the information of age, gender, marriage, residential area, educational level, working state, stroke type, family history, number of strokes, monthly income, primary caregiver, sleep conditions, and number of chronic diseases.

The stroke recurrence risk perception scale（SRRPS）

For peer review only - http://bmjopen.bmj.com/site/about/guidelines.xhtml

The stroke recurrence risk perception scale is a tool used to assess stroke patients' recurrence risk perception of their disease. Our research group [16] developed the scale in 2022, which includes two sections with 20 items. The first section contains three items that assess recurrence risk perception of patients with stroke. The second section contains 17 items in three domains: perceived illness risk factors, perceived behavioral risk factors, and perceived severity. There are four items in the “perceived illness risk factors” dimension, six items in the “perceived behavioral risk factors” dimension and seven items in the “perceived severity” domain. Higher scores indicate greater risk awareness, and the score for this section is calculated independently of the overall score. The second section of items ranges from 1-3 (disagree, not clear, agree). The Cronbach's alpha of the second section is 0.850 and the Cronbach's alpha for each dimension are 0.875, 0.815, and 0.804. This scale was applied by Tang in 236 patients with first-episode ischemic stroke patients in China and the Cronbach's alpha for the second part was 0.890 [35]. The validity of this scale has also been verified by validation factor analysis in another study [36]. In this study, we used the second section of the scale to examine stroke patients' perception of recurrence risk.

The perceived social support scale (PSSS)

The perceived social support scale is an instrument to measure the individual's self-perception of multi-dimensional social support. The scale was developed by Zimet in 1987 and revised into a Chinese version by Jiang [37]in 1999. It includes three dimensions of family support, friend support, and other support (relatives, leaders, colleagues, etc.), with 12 items. Each dimension has 4 items. It is a 7-point Likert scale (from 1 = extremely uncompliant to 7 =extremely compliant). The total score ranges from 12-84 points, with scores of 12 to 36 points being low perceived support level, 37 to 60 points being middle perceived support level, and 61 to 84 points being high perceived support level. The Cronbach's alpha value of PSSS is 0.92.

The health behavior scale for stroke patients (HBS-SP)

The health behavior scale for stroke patients is a scale to assess the level of health behavior in patients with stroke. The scale was developed by Wan et al [38] in 2017. It includes 6 dimensions of exercise (6 items), medication taking (4 items), instructions (4 items), responsibility (3 items), nutrition (6 items), and smoking and alcohol (2 items), with 25 entries. It is a 4-point Likert scale (from 1 = never to 4 = routinely performed). But the items of medication and tobacco, and alcohol were reversed. Higher scores indicate better health behavior. An average score of 2.5 points (between “sometimes” and “often”) is considered an intermediate level of health behavior. The Cronbach's alpha value of HBS-SP scale is 0.878.

Statistical analysis

The Statistical Package of Social Science software version 26 (IBM Corp. Released, 2017) was used to analyze the data. Participants’ social demographics characteristics and other related variables were examined by descriptive statistics of frequencies and percentages. Pearson correlation analysis was used to analyze the interrelationships between the three variables. We applied the testing procedure recommended by Wen [39] and the mediation model was analyzed using Model 4 in the PROCESS Marco [40]. For the best test of the mediation effect, we carried out the bootstrapping procedure to measure the indirect effect and 95% confidence interval (*CI*) were estimated. $P<0.05$ was considered statistically significant. A 95% confidence interval excluding zero was regarded as statistically significant.

Patient and public involvement

Patients or the public were not involved in the design, conduct, reporting or dissemination of our research.

For peer review only - http://bmjopen.bmj.com/site/about/guidelines.xhtml

Results

General Demographics

A total of 254 questionnaires were obtained. However, 4 invalid questionnaires were removed because the questionnaires with a completion rate of less than 95%. Finally, the number of effective participants was 250, and the final effective rate of questionnaire collection was 98.4%. Over half (70.8%) of patients were elderly patients (≥60 years). Less than half of the patients in the sample had a primary school degree or less (46.1%). Moreover, 34.8% were working and a majority (97.6%) of patients with stroke were ischemic stroke. In addition, 50.4% of stroke patients were low and middle income group. 52% of them were first-episode patients. Comparison of the scales' scores of stroke patients with different characteristics are shown in Supplemental Table.

Descriptive statistics of the SRRPS, PSSS and HBS-SP

Table 1 displayed mean scores, standard deviations and ranges of SRRPS, PSSS and HBS-SP. The mean and SD of stroke recurrence risk perception scale were 41.04±8.64 scores. The mean and SD of perceived social support scale were 65.86±8.78 scores. The mean and SD of perceived social support scale were 56.78±9.03 scores. Therefore, among the patients with stroke we recruited, recurrence risk perception and perceived social support were at high levels. Health behavior was at a moderately low level.

Table 1 Scores on the SRRPS, PSSS and HBS-SP

Variables	Items	Range of score	Mean	Stand deviation
SRRPS	17	17~51	41.04	8.64
perceived illness risk factors	4	6~18	19.96	4.76
perceived behavioral risk factors	6	4~12	22.80	3.35
perceived severity	7	7~21	17.84	4.05
PSSS	12	40~80	65.86	8.78
family support	4	13~28	23.08	3.58
friend support	4	7~28	19.97	4.77
other support	4	12~28	22.80	3.35
HBS-SP	25	28~81	56.78	9.03
exercise	6	5~24	11.83	4.81
medication taking	4	5~19	11.74	2.66
instructions	4	4~17	8.81	2.58
responsibility	3	3~8	3.25	3.91
nutrition	6	6~24	14.48	1.69
smoking and alcohol	2	2~8	6.66	0.73

Abbreviations: SRRPS, stroke recurrence risk perception scale; PSSS, Perceived social support scale; HBS-SP, Health behavior scale for stroke patients

Correlation Analysis between SRRPS, PSSS and HBS-SP

Pearson's correlation analysis was used to investigate the relationships among recurrence risk perception, health behavior, and perceived social support in patients with stroke. Briefly, recurrence risk perception was positively correlated with health behavior (r=0.679, $P<0.001$), perceived social support was positively correlated with health behavior (r=0.778, $P<0.001$), recurrence risk perception was positively correlated with perceived social support (r=0.639, $P<0.001$), meaning that recurrence risk perception, health behavior, and perceived social support were positively correlated with each other in patients with stroke.

For peer review only - http://bmjopen.bmj.com/site/about/guidelines.xhtml

Mediation Analysis of SRRPS, PSSS and HBS-SP

Building on our initial findings from the correlation analysis, we next performed mediation analysis to explore the potential association underlying the relationship between an individual's perceived social support, their perception of stroke recurrence risk, and their adoption of healthy behaviors among stroke patients. We controlled for some general demographic factors (like age, gender, monthly income, education) which are considered as important predictors affect health behavior in patients with stroke in China. In model 1, recurrence risk perception had a positive predictive effect on health behavior (β= 0.607, $P < 0.001$). In model 2, recurrence risk perception had a positive predictive effect on perceived social support (β= 0.604, $P < 0.001$). In model 3, recurrence risk perception and perceived social support positively predicted health behavior (β= 0.247, β= 0.596, $P < 0.001$), but the comparison of the effect coefficients showed that the effect of recurrence risk perception on health behavior was diminished from 0.607 to 0.247, which meant that perceived social support partially mediated the effect between recurrence risk perception and health behavior, as shown in Table 2.

Table 2 Mediation results for recurrence risk perception predicting health behavior mediated by perceived social support

Recurrence risk perception → Health behavior				
	SE	β	t	P
Age	0.968	-1.246	-1.287	0.200
Gender	0.863	0.958	1.110	0.268
Education	0.460	1.187	2.579	0.011
Monthly Income	0.742	1.158	1.561	0.120
Recurrence risk perception	0.053	0.607	11.529	<0.001
Recurrence risk perception →Perceived social support				
Gender	0.890	0.912	1.026	0.306
Education	0.474	1.669	3.522	0.005
Monthly Income	0.764	-1.514	-1.981	0.049
Recurrence risk perception	0.054	0.604	11.137	<0.001
Recurrence risk perception, Perceived social support → Health behavior				
Age	0.766	-1.389	-1.812	0.071
Gender	0.685	0.415	0.607	0.545
Education	0.373	0.193	0.516	0.606
Monthly Income	0.592	2.059	3.480	0.006
Recurrence risk perception	0.051	0.247	4.833	<0.001
Perceived social support	0.049	0.596	12.110	<0.001

To ensure the accuracy of the test, this study further used the bootstrap method [40] to test the mediating effect of perceived social support between recurrence risk perception and health behavior with 5000 replicate samples. The results are shown in Table 4, and the 95% confidence interval of the mediating effect of perceived social support was [0.26,0.46], which did not contain zero, indicating that the mediating effect of perceived social support in the process of recurrence risk perception affecting health behavior was established, and the effect accounted for 59.31% (Table 3). The mediating model of perceived social support is constructed in Figure 2.

For peer review only - http://bmjopen.bmj.com/site/about/guidelines.xhtml

Table 3 Mediating model examination by bootstrap

Recurrence risk perception → Health behavior				
	Effect	SE	LL 95%*CI*	UL 95%*CI*
Indirect effect	0.360	0.050	0.263	0.460
Direct effect	0.247	0.512	0.147	0.348

Abbreviations: SE, standard error; LL 95% CI, Lower Limit of 95% confidence interval; UL 95%CI, Upper Limit of 95% confidence interval

Insert Figure 2 here.

Discussion

We found that 78.4% of patients with stroke scored less than 2.5 on the health behavior scale, which meant they had moderately low health behavior. This finding was consistent with previous studies. Li et al [41] investigated 462 patients with stroke in three tertiary hospitals in Liaoning Province, China, and found that patients with stroke had moderate health behavior. The same results were reaffirmed in another study [42]. More than half of the 231 patients with stroke had moderately low health behavior. This highlighted the urgent need for further research and focus on the influencing factors of healthy behavior among stroke patients.

This study demonstrated that perceived social support was positively correlated with health behavior. This finding was consistent with previous studies [43,44]. A cross-sectional study [43] based on data from the Spanish National Health Survey (ENSE2017) with a final sample of 1006 individuals with diabetes mellitus has shown the perceived social support was positively correlated with physical activity. The same results were confirmed in another study. Guo et al [44] conducted a community-based cross-sectional study among 1697 participants with hypertension in China and found social support had a direct positively effect on medication adherence. Moreover, Bandura's social theory [45] also proposed that factors such as social support were actual aspects of the incidence of behavior. Brouwer-Goossensen has found social support to be a positive factor in health behavior change in interviews with 18 stroke patients [46]. Our study provided further evidence of the relationship between perceived social support and health behavior. Therefore, perceived social support is an important factor in improving health behavior in patients with stroke. In addition, there was a significant positive correlation between recurrence risk perception and perceived social support. As a result, our initial hypothesis was contradicted by the findings of our study. In other words, recurrence risk perception affects the perceived social support directly and positively actually. We speculated the reason for this result was that the higher stroke patients' recurrence risk perception, the more control they had over their diseases. This sense of control over the disease could partly increase their self-efficacy [47] to stay in a good mind, promoting a positive mindset, and concurrently enhancing their perception of social support. Calderon et al [48] discovered that a majority of chronic patients expressed a desire for increased information and greater involvement in the decision-making process. To the best of our knowledge, our study was the first study to investigate the association between stroke patients' perceptions of recurrence and their perceived social support in China.

Most importantly, we found that recurrence risk perception appeared to influence health behavior via two pathways, a direct pathway and an indirect pathway mediated by perceived social support. (1) First pathway, recurrence risk perception was correlated with health behavior significantly and positively. This was contrary to our research hypothesis. It may be due to the fact that stroke patients did not view the risk of recurrence as a frightening event. They gradually increased their confidence in their own recovery as they acquired more knowledge about the risk instead. In this way, they were better able to cope with the disease. For example,

For peer review only - http://bmjopen.bmj.com/site/about/guidelines.xhtml

they began to adopt healthy behaviors to prevent recurrence. In fact, this findings were consistent with the health belief model (HBM) [18]. Besides, our previous research found patients with stroke who lacked a perception of the risk of recurrence and preventive strategies exhibited a tendency to limit their regular hospital visits or refrain from adopting healthy behaviors aimed at preventing recurrence [40]. In other words, recurrence risk perception was an important positive factor of health behavior. (2) The second pathway was that recurrence risk perception mediated the effect of perceived social support on health behavior. To effectively implement secondary stroke prevention measures, it is crucial for patients with stroke to have a comprehensive understanding of the risk factors associated with recurrent stroke. Their understanding of the lifestyle changes needed to reduce the chance of future recurrences is equally crucial [49]. In patients with stroke, the perceived risk of recurrence involves not only just concern and fear, but also the understanding of early warning signs and risk factors associated with stroke recurrence. So patients with stroke with a higher recurrence risk perception also demonstrated a greater depth of stroke knowledge [50], and these patients showed a higher level of confidence in recovery than patients with a lower perceived risk of recurrence. Therefore, they perceive information or emotional support from those around them better. The presence of these social supports played a significant role in improving their emotional well-being, consequently fostering a greater willingness to embrace healthier behaviors.

Overall, our study found that recurrence risk perception not only directly and positively influenced health behaviors of patients with stroke, but also further influenced health behavior by positively affecting perceived social support. This was consistent with the connotation of the CSM model [25]. In other words, our findings provided practical support for the CSM model. As the practical implication is that our finding may aid future research in identifying entry points for health behavior interventions by taking the methods that improve stroke patients' perception of recurrence risk and perceived social support. For example, health care providers could consider providing patients with recurrence risk communication. Specifically, healthcare professionals can help patients to have an accurate understanding of the risk of recurrence. First, a comparison between the subjective and objective risk perception scores for stroke recurrence is necessary for healthcare professionals. If the stroke recurrence risk perception and objective risk perception scores are inconsistent, then risk communication should be initiated with stroke patients [51]. Common risk communication tools mainly include graphical [52] and numerical forms [53]. To enable patients correctly grasp their rates of recurrence, appropriate risk communication strategies should be chosen according to their various level of education and preferences. In addition, healthcare professionals can provide stroke patients with the necessary knowledge and skills to prevent recurrence, such as the aura of stroke recurrence, risk factors of recurrence, and prognosis of recurrence. By doing this, stroke patients' perceptions of social support can be enhanced, which will ultimately help them adopt healthy behaviors. It can also boost their confidence in their ability to recover.

The study still has some limitations. Firstly, it was employed a cross-sectional design, thereby precluding the establishment of a causal relationship between recurrence risk perception and health behavior. Nonetheless, the study successfully confirmed a significant correlation between recurrence risk perception and health behavior. Secondly, despite our efforts to incorporate multi-center sampling, the impact of COVID-19 necessitated the restriction of our study to a single tertiary hospital in Henan Province. Therefore, the findings may not be generalizable to all patients with stroke. Besides, only six hemorrhagic patients with stroke were included in our study, so it was difficult for us to explore differences in stroke type in patients' perceived risk of recurrence, perceived social support and health behavior. Lastly, our study included a relatively elderly population of patients with stroke, which may have some impact on the generalizability of the findings. Therefore, we recommend the design of a multi-center longitudinal study specifically focusing on young stroke patients in future research. Such a study design would help clarify the dynamic trends in recurrence risk perception, perceived social support, and health behavior over time.

Conclusion

For peer review only - http://bmjopen.bmj.com/site/about/guidelines.xhtml

In conclusion, this study found that perceived social support mediated the relationship between recurrence risk perception and health behavior. Our findings expand upon previous research on the interplay between recurrence risk perception, perceived social support, and health behavior in patients with stroke. The results of this study can serve as a useful guide for future clinical nursing practice and research in this area. Considering the importance of recurrence risk perception and perceived social support, it is recommended that these factors be incorporated into the assessment process for patients with stroke, both during hospitalization and follow-up care. We suggest that healthcare professionals should assist patients with stroke in developing an accurate perception of recurrence risk with appropriate recurrence risk communication approaches. This method can significantly improve patients' capacity to identify and access social support, ultimately enhancing their confidence in recovery and motivation to engage in healthy behaviors. Moreover, it is crucial to recognize the significance of social support from family and friends as a primary source of perceived social support. Healthcare professionals should encourage and educate patients' loved ones to offer emotional support, which can positively impact patients' recovery and well-being.

Acknowledgements

We would like to acknowledge the tertiary hospital of Henan province for their cooperation. We greatly appreciate the support of all patients for their voluntary participation in this study.

Contributors

ZZX and LBL designed the study. WXX, JH and WWN contributed to writing and revising. WXX, MYX and ZCH were in charge of data collection and analysis. ZQS and CSY were in charge of study design and essential help. All authors read and approved the final manuscript.

Funding

This work was supported by the National Natural Science Foundation of China Youth Science Foundation Project (72104221).

Competing interests

None declared.

Patient consent for publication

Each participant gave informed written consent and permission to publish before analysis of the study.

Ethics approval

The Research Ethics Committee of Zhengzhou University (ZZURIB2020-08) approved the study.

Provenance and peer review

Not commissioned; externally peer reviewed.

Data availability statement

Data are available upon reasonable request.

For peer review only

For peer review only - http://bmjopen.bmj.com/site/about/guidelines.xhtml

References

1 Chen Y, Wright N, Guo Y, *et al.* Mortality and recurrent vascular events after first incident stroke: a 9-year community-based study of 0·5 million Chinese adults. *Lancet Glob Health*. 2020;8:e580–90.

2 Gladstone DJ, Lindsay MP, Douketis J, *et al.* Canadian Stroke Best Practice Recommendations: Secondary Prevention of Stroke Update 2020. *Can J Neurol Sci*. 2022;49:315–37.

3 Kleindorfer DO, Towfighi A, Chaturvedi S, *et al.* 2021 Guideline for the Prevention of Stroke in Patients With Stroke and Transient Ischemic Attack: A Guideline From the American Heart Association/American Stroke Association. *Stroke*. 2021;52:e364–467.

4 Liu L, Chen W, Zhou H, *et al.* Chinese Stroke Association guidelines for clinical management of cerebrovascular disorders: executive summary and 2019 update of clinical management of ischaemic cerebrovascular diseases. *Stroke Vasc Neurol*. 2020;5:159–76.

5 O'Donnell MJ, Xavier D, Liu L, *et al.* Risk factors for ischaemic and intracerebral haemorrhagic stroke in 22 countries (the INTERSTROKE study): a case-control study. *Lancet*. 2010;376:112–23.

6 Abdullahi SU, Sunusi S, Abba MS, *et al.* Hydroxyurea for secondary stroke prevention in children with sickle cell anemia in Nigeria: a randomized controlled trial. *Blood*. 2023;141:825–34.

7 Liu Y, Wei M, Guo L, *et al.* Association between illness perception and health behaviour among stroke patients: The mediation effect of coping style. *Journal of Advanced Nursing*. 2021;77:2307–18.

8 Mugwano I, Kaddumukasa M, Mugenyi L, *et al.* Poor drug adherence and lack of awareness of hypertension among hypertensive stroke patients in Kampala, Uganda: a cross sectional study. *BMC Res Notes*. 2016;9:3.

9 Wu X, Min L, Cong L, *et al.* Sex differences in health-related quality of life among adult stroke patients in Northeastern China. *J Clin Neurosci*. 2014;21:957–61.

10 Kim G-G, Chae D-H, Park M-S, *et al.* Factors Influencing 1-Year Medication Adherence of Korean Ischemic Stroke Survivors. *Int J Behav Med*. 2020;27:225–34.

11 Parappilly BP, Field TS, Mortenson WB, *et al.* Determinants Influencing the Prestroke Health Behaviors and Cardiovascular Disease Risk of Stroke Patients: A Cross-Sectional Study. *J Stroke Cerebrovasc Dis*. 2019;28:1509–18.

12 Santos M, Kövari E, Gold G, *et al.* The neuroanatomical model of post-stroke depression: towards a change of focus? *J Neurol Sci*. 2009;283:158–62.

13 Brouwer-Goossensen D, Genugten L van, Lingsma H, *et al.* Determinants of intention to change health-related behavior and actual change in patients with TIA or minor ischemic stroke. *Patient Educ Couns*. 2016;99:644–50.

14 Ren H, Guo Y-F, Zhang Z-X, *et al.* Perception of recurrent risk versus objective measured risk of ischemic stroke in first-ever stroke patients from a rural area in China: A cross-sectional study. *Patient Educ Couns*. 2023;107:107586.

15 Holder C, Krishnamurthi R, Theadom A. Exploring facilitators and barriers to long-term behavior change following health-wellness coaching for stroke prevention: A qualitative study conducted in Auckland, New Zealand. *Brain Behav*. 2023;13:e2671.

16 Lin B, Zhang Z, Guo Y. Development and psychometric test of Recurrence Risk Perception Scale for Patients with Stroke. *Chinese Journal of Nursing*. 2022;56:1666–71.

17 Thakkar J, Heeley EL, Chalmers J, *et al.* Inaccurate risk perceptions contribute to treatment gaps in secondary prevention of cardiovascular disease. *Intern Med J*. 2016;46:339–46.

18 Champion VL, Skinner CS. The health belief model. *Health behavior and health education: Theory, research, and practice, 4th ed*. San Francisco, CA, US: Jossey-Bass 2008:45–65.

19 Iversen AB, Blauenfeldt RA, Johnsen SP, *et al.* Understanding the seriousness of a stroke is essential for appropriate help-seeking and early arrival at a stroke centre: A cross-sectional study of stroke patients and their bystanders. *Eur Stroke J*. 2020;5:351–61.

20 Freeman-Gibb LA, Janz NK, Katapodi MC, *et al.* The relationship between illness representations, risk perception and fear of cancer recurrence in breast cancer survivors. *Psychooncology*. 2017;26:1270–7. doi: 10.1002/pon.4143

21 Lee-Jones C, Humphris G, Dixon R, *et al.* Fear of cancer recurrence--a literature review and proposed cognitive

For peer review only - http://bmjopen.bmj.com/site/about/guidelines.xhtml

formulation to explain exacerbation of recurrence fears. *Psychooncology*. 1997;6:95–105. doi: 10.1002/(SICI)1099-1611(199706)6:2<95::AID-PON250>3.0.CO;2-B

22 Blanton S, Scheibe DC, Rutledge AH, *et al.* Family-Centered Care During Constraint-Induced Therapy After Chronic Stroke: A Feasibility Study. *Rehabil Nurs*. 2019;44:349–57.

23 Northcott S, Moss B, Harrison K, *et al.* A systematic review of the impact of stroke on social support and social networks: associated factors and patterns of change. *Clin Rehabil*. 2016;30:811–31.

24 Dahlan R, Ghazal E, Saltaji H, *et al.* Impact of social support on oral health among immigrants and ethnic minorities: A systematic review. *PLoS One*. 2019;14:e0218678.

25 Leventhal H, Phillips LA, Burns E. The Common-Sense Model of Self-Regulation (CSM): a dynamic framework for understanding illness self-management. *J Behav Med*. 2016;39:935–46. doi: 10.1007/s10865-016-9782-2

26 Leventhal H, Diefenbach M, Leventhal E. Illness cognition: Using common sense to understand treatment adherence and affect cognition interactions. *Cognitive Therapy and Research*. 1992;16:143–63.

27 Fall E, Chakroun-Baggioni N, Böhme P, *et al.* Common sense model of self-regulation for understanding adherence and quality of life in type 2 diabetes with structural equation modeling. *Patient Educ Couns*. 2021;104:171–8. doi: 10.1016/j.pec.2020.06.023

28 Johnston KN, Burgess R, Kochovska S, *et al.* Exploring the Experience of Breathlessness with the Common-Sense Model of Self-Regulation (CSM). *Healthcare (Basel)*. 2023;11:1686.

29 Hagger MS, Orbell S. The common sense model of illness self-regulation: a conceptual review and proposed extended model. *Health Psychol Rev*. 2022;16:347–77.

30 Adamson M, Cotoc C, Choi K, *et al.* Cancer Communication Outside of the Physician-Patient Relationship: The Experience of Communicating and Understanding the Meaning of Prognosis. *Am J Hosp Palliat Care*. 2019;36:711–7.

31 Singh T, Newby DE. Is the fear of disease worse than the disease itself? *Heart*. 2021;107:91–2.

32 Neurology CS of, Society CS. Diagnostic criteria of cerebrovascular diseases in China (version 2019). *Chinese Journal of Neurology*. 2019;52:710–5. doi: 10.3760/cma.j.issn.1006-7876.2019.09.003

33 Gao C, Wu C, Zhao J, *et al.* Guidelines for the Diagnosis and Treatment of Cerebral Infarction in China (2017). *Chinese Journal of Integrated Traditional and Western Medicine*. 2018;38:136–44.

34 Ni P, Chen J, Liu N. Sample size estimation in quantitative nursing research. *Chinese Journal of Nursing*. 2010;45:378–80.

35 Tang B, Lv Y, Wang X, *et al.* Analysis of status and influencing factors of recurrence risk perception in first-episode ischemic stroke patients. *Chinese Journal of Nursing*. 2023;58:289–95.

36 Gao F, An Q, Liu D, *et al.* Recurrence risk perception and its correlates in stroke patients. *Journal of Nursing(China)*. 2023;38:30–3.

37 Wang X, Wang X, Ma H. *Manual of the Mental Health Rating Scale*. Beijing: Chinese Journal of Mental Health 1999.

38 Wan L, Xiong X, Pan J, *et al.* Development and reliability as well as validity test of Health Behavior Scale for stroke patients. *Journal of Nursing Science*. 2017;32:25–9.

39 Wen Z. Analyses of Mediating Effects: The Development of Methods and Models. *Advances in psychological Science*. 2014;22:731–45.

40 Henderson AR. The bootstrap: a technique for data-driven statistics. Using computer-intensive analyses to explore experimental data. *Clin Chim Acta*. 2005;359:1–26.

41 Li S, He H, Li H, *et al.* Chain Mediating Effects of Executive Intention of Rehabilitation Exercise Behaviors and Mental Health Literacy between Perceived Social Support and Health Behaviors among Stroke Inpatients. *Military Nursing*. 2023;40:34–8.

42 Liu M, Liang F, Yin Y, *et al.* Latent Profile Analysis of Health Literacy and Comparison of Health Behavior Differences in Patients with Stroke. *Military Nursing*. 2023;40:52-55+70.

43 Denche-Zamorano A, Pisà-Canyelles J, Barrios-Fernandez S, *et al.* Is Psychological Distress Associated with Self-Perceived Health, Perceived Social Support and Physical Activity Level in Spanish Adults with Diabetes? *J Pers Med*. 2023;13:739.

For peer review only

For peer review only - http://bmjopen.bmj.com/site/about/guidelines.xhtml

44 Guo A, Jin H, Mao J, *et al.* Impact of health literacy and social support on medication adherence in patients with hypertension: a cross-sectional community-based study. *BMC Cardiovasc Disord*. 2023;23:93. doi: 10.1186/s12872-023-03117-x

45 Bandura A. Social cognitive theory: an agentic perspective. *Annu Rev Psychol*. 2001;52:1–26.

46 Brouwer-Goossensen D, den Hertog HM, Mastenbroek-de Jong MA, *et al.* Patient perspectives on health-related behavior change after transient ischemic attack or ischemic stroke. *Brain Behav*. 2021;11:e01993. doi: 10.1002/brb3.1993

47 Al Shafaee MA, Ganguly SS, Al Asmi AR. Perception of stroke and knowledge of potential risk factors among Omani patients at increased risk for stroke. *BMC Neurol*. 2006;6:38.

48 Calderon C, Jiménez-Fonseca P, Ferrando PJ, *et al.* Psychometric properties of the Shared Decision-Making Questionnaire (SDM-Q-9) in oncology practice. *Int J Clin Health Psychol*. 2018;18:143–51.

49 Hiraga A. Perception of recurrent stroke risk among stroke survivors. *Neuroepidemiology*. 2011;37:88–9.

50 Slark J, Bentley P, Majeed A, *et al.* Awareness of stroke symptomatology and cardiovascular risk factors amongst stroke survivors. *J Stroke Cerebrovasc Dis*. 2012;21:358–62.

51 Chen DW, Reyes-Gastelum D, Wallner LP, *et al.* Disparities in risk perception of thyroid cancer recurrence and death. *Cancer*. 2020;126:1512–21. doi: 10.1002/cncr.32670

52 Goodyear-Smith F, Arroll B, Chan L, *et al.* Patients Prefer Pictures to Numbers to Express Cardiovascular Benefit From Treatment. *The Annals of Family Medicine*. 2008;6:213–7.

53 Fair AKI, Murray PG, Thomas A, *et al.* Using hypothetical data to assess the effect of numerical format and context on the perception of coronary heart disease risk. *American Journal of Health Promotion*. 2008;22:291–6.

Figure 1 The hypothesis model of recurrence risk perception, health behavior and perceived social support in patients with stroke

Figure 2 Mediating effects of perceived social support on the relationship between recurrence risk perception and health behavior (**P* < 0.001)

For peer review only

For peer review only - http://bmjopen.bmj.com/site/about/guidelines.xhtml

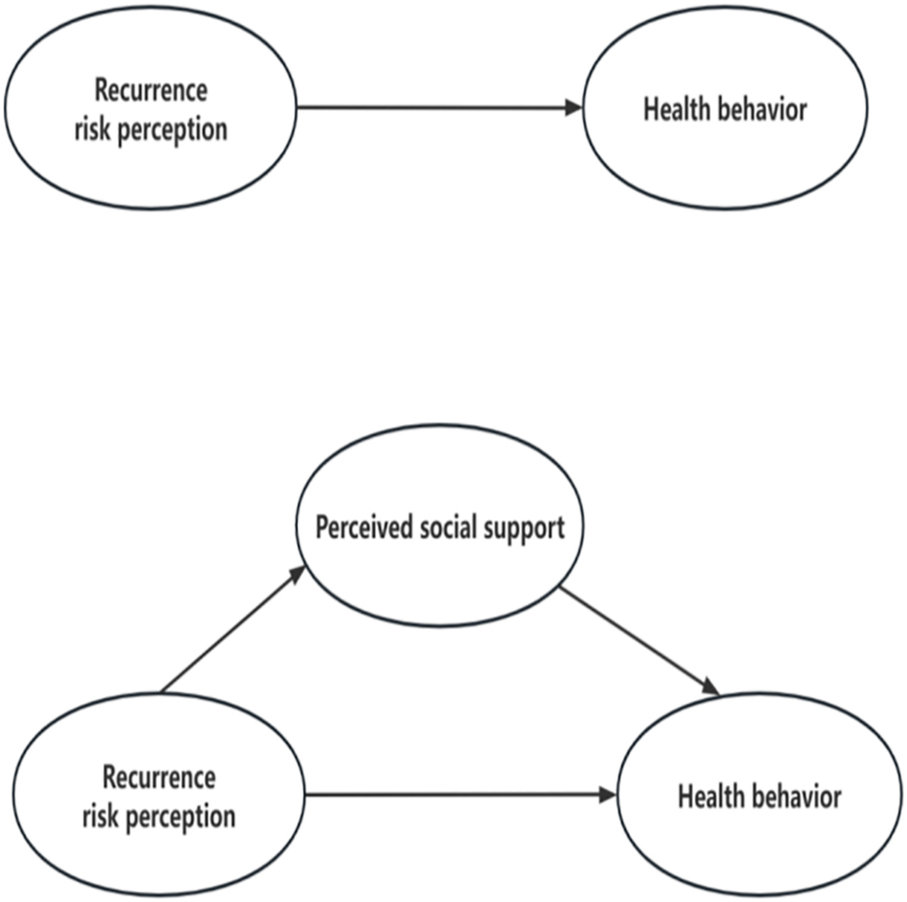

90x90mm (300 x 300 DPI)

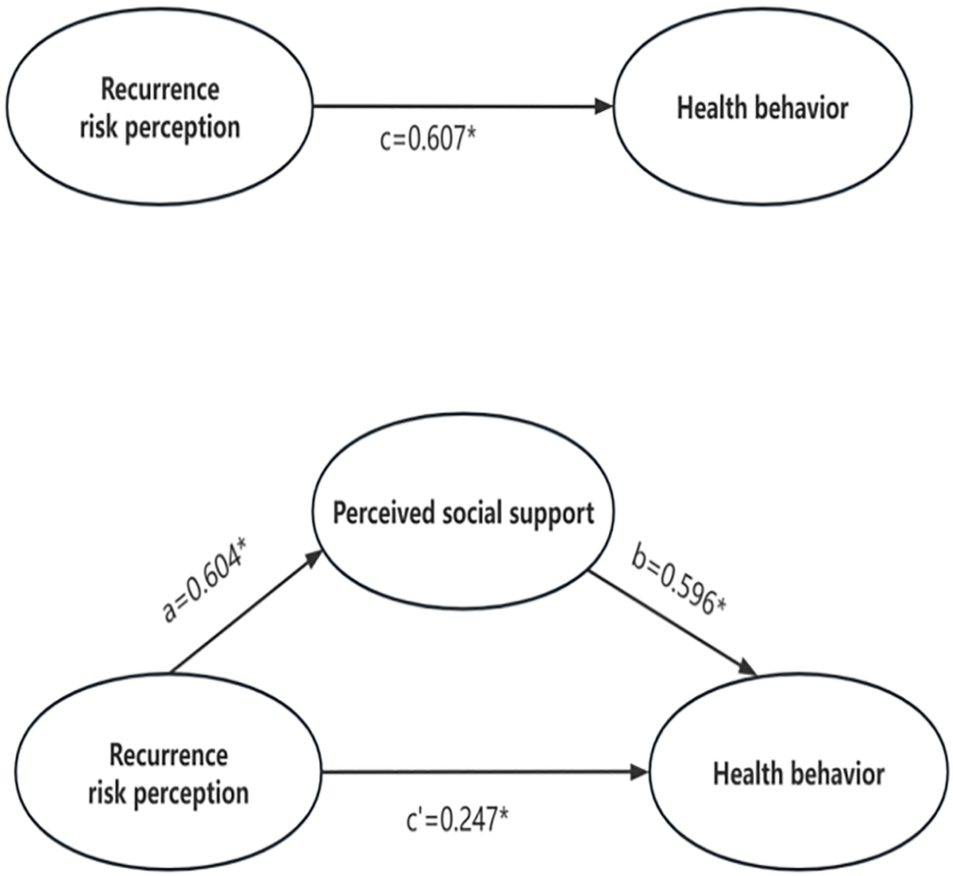

90x90mm (300 x 300 DPI)

Supplemental Table 1 Comparison of recurrence risk perception scores of stroke patients with different characteristics

Factors	Group	*N*	%	SRRPS	Perceived illness risk factors	Perceived behavioral risk factors	Perceived severity
Gender	Male	136	54.4	40.88±9.73	14.68±4.03	8.71±2.38	17.43±4.45
	Female	114	45.6	41.23±7.15	14.66±3.60	8.24±2.30	18.33±3.48
Marriage	Married	220	88	41.46±8.23	14.92±3.71	8.60±2.29	17.95±3.85
	Divorced	2	0.8	47.00±0.00	17.00±1.41	9.50±2.12	20.50±0.71
	Unmarried	28	11.2	37.29±10.91	12.50±4.24	7.71±2.79	16.82±5.40
Age	<60	73	29.2	44.55±6.44	15.93±2.83	9.59±2.06	19.03±2.93
	≥60	177	70.8	39.59±9.02	14.15±4.07	8.04±2.32	17.84±4.05
Residential area	Rural	126	50.4	39.92±7.91	14.08±3.91	8.10±2.17	17.68±3.79
	Urban	124	49.6	42.17±9.21	15.27±3.68	8.89±2.46	18.01±4.32
Working state	Unemployed	54	21.6	40.11±7.29	13.48±3.30	7.85±2.28	18.78±3.66
	Pensioner	109	43.6	39.37±9.98	14.16±4.22	8.18±2.47	17.03±4.66
	Working	87	34.8	43.70±6.85	16.06±3.22	9.28±2.04	18.29±3.23
Educational level	Primary and below	115	46	38.72±7.99	13.56±3.97	7.82±2.06	17.29±4.06
	Junior high school	65	27.6	40.49±8.61	14.75±3.35	8.22±2.28	17.52±4.28
	High school	44	17.6	42.81±9.35	15.50±3.99	9.11±2.46	18.20±4.18
	College	14	5.6	48.64±2.21	18.00±0.00	10.57±1.60	20.07±2.06
	University and above	12	4.8	50.75±0.45	18.00±0.00	11.75±0.45	21.00±0.00
Stroke type	Hemorrhagic stroke	6	2.4	47.50±3.74	17.25±1.04	10.00±2.14	20.25±1.04
	Ischemic stroke	244	97.6	40.82±8.67	14.59±3.86	8.44±2.34	17.76±4.09

Family history	Have	25	10	38.36±10.60	13.32±4.96	8.08±2.33	16.96±4.33
	No	255	90	41.33±8.37	14.82±3.67	8.54±2.35	17.94±4.02
Monthly income (RMB)	≤2999	89	35.6	37.62±7.98	13.08±3.81	7.56±2.00	16.90±4.22
	3000-4999	126	50.4	42.59±8.39	15.52±3.64	8.75±2.27	18.32±3.90
	≥5000	35	14	44.14±8.56	15.69±3.31	9.91±2.56	18.54±3.85
Numbers of stroke	1 time	130	52	41.54±8.19	15.38±3.42	8.78±2.05	17.38±3.99
	2 times	74	29.6	43.15±6.12	15.04±3.18	8.88±2.16	19.23±2.70
	3 times	22	8.8	42.27±8.14	14.27±4.08	8.27±3.01	19.41±3.70
	More than 3 times	24	9.6	30.67±11.07	10.08±4.57	5.92±2.30	14.67±5.73
Primary caregiver	Spouse or children	132	52.8	17.30±4.46	14.30±4.16	8.17±2.42	39.82±9.54
	Babysitter or nursing workers	11	4.4	38.00±11.12	14.73±4.47	14.73±4.47	15.73±5.10
	Own	107	42.8	42.85±6.71	15.12±3.31	8.99±2.22	18.74±3.15
Number of chronic diseases	0	75	30	40.15±8.96	14.07±3.80	8.15±2.15	17.93±3.94
	1~2	150	60	41.29±9.17	14.75±4.04	8.67±2.43	17.83±4.17
	3 and above	25	10	42.20±6.87	16.04±1.86	8.48±2.40	17.68±3.87
Sleep conditions	Very good	92	36.8	40.07±9.91	14.12±4.20	8.35±2.55	17.52±4.60
	Good	69	27.6	42.51±8.25	15.25±3.78	8.81±2.37	18.45±3.47
	Poor	69	27.6	39.65±7.86	14.23±3.61	8.28±2.28	17.14±4.21
	Very poor	20	8	45.20±2.19	16.75±1.55	8.80±1.32	19.65±0.88

Supplemental Table 2 Comparison of perceived social support scores of stroke patients with different characteristics

Factors	Group	*N*	%	PSSS	Family support	Friend support	Other support
Gender	Male	136	54.4	65.50±9.07	22.65±3.89	20.43±4.32	22.42±3.48
	Female	114	45.6	66.29±8.43	23.60±3.11	19.41±5.22	23.28±3.15
Marriage	Married	220	88	66.27±8.55	23.17±3.49	20.15±4.84	22.95±3.15
	Divorced	2	0.8	69.00±12.73	22.50±3.54	23.00±4.24	23.50±4.95
	Unmarried	28	11.2	62.43±9.88	22.39±4.26	18.36±3.96	21.68±4.60
Gender	＜60	73	29.2	68.64±8.56	23.90±2.92	21.11±5.21	23.63±2.74
	≥60	177	70.8	64.71±8.63	22.75±3.78	19.50±4.51	22.47±3.52
Residential area	Rural	126	50.4	65.54±8.14	23.23±3.08	19.37±4.90	22.94±3.31
	Urban	124	49.6	66.19±9.40	22.94±4.04	20.58±4.57	22.67±3.40
Working state	Unemployed	54	21.6	64.33±8.70	22.67±3.39	19.57±4.96	22.09±3.54
	Pensioner	109	43.6	65.01±8.81	22.76±3.82	19.71±4.78	22.54±3.34
	Working	87	34.8	67.87±8.51	23.75±3.32	20.54±4.64	23.59±3.12
Educational level	Primary and below	115	46	64.03±7.79	22.57±3.31	18.92±4.90	22.53±3.06
	Junior high school	65	27.6	64.07±8.83	22.32±3.79	19.77±4.17	21.97±3.82
	High school	44	17.6	68.37±8.74	23.80±3.57	20.95±4.73	23.61±3.19
	College	14	5.6	71.36±7.96	25.36±3.03	21.29±3.95	24.71±3.20
	University and above	12	4.8	77.58±4.54	26.83±1.57	25.92±.151	25.83±2.04
Stroke type	Hemorrhagic stroke	6	2.4	73.88±7.38	26.50±1.60	21.63±5.58	25.75±1.16
	Ischemic stroke	244	97.6	65.60±8.71	22.97±3.57	19.91±4.74	22.71±3.36
Family history	Have	25	10	66.00±8.56	23.28±2.57	19.24±5.33	23.49±3.38
	No	255	90	65.84±8.82	23.06±3.68	20.05±4.71	22.73±3.35
Monthly income (RMB)	≤2999	89	35.6	64.73±8.07	22.79±3.48	19.10±4.46	33.84±3.44

	3000-4999	126	50.4	65.67±8.59	23.09±3.46	19.94±5.01	22.65±3.08
	≥5000	35	14	69.40±10.38	23.83±4.22	22.23±3.92	23.29±4.06
Numbers of stroke	1 time	130	52	65.88±9.05	22.90±3.62	20.18±4.84	22.80±3.35
	2 times	74	29.6	67.03±7.36	23.88±2.91	20.18±4.63	22.97±2.93
	3 times	22	8.8	67.27±9.19	23.59±3.65	20.32±4.47	23.36±3.47
	More than 3 times	24	9.6	60.83±9.63	21.17±4.45	17.83±4.82	21.83±4.34
Primary caregiver	Spouse or children	132	52.8	64.95±8.77	23.23±3.77	18.86±5.03	22.86±3.54
	Babysitter or nursing workers	11	4.4	58.73±8.00	19.64±3.80	19.55±1.51	19.55±4.25
	Own	107	42.8	67.71±8.37	23.25±3.15	21.37±4.29	23.08±2.83
Number of chronic diseases	0	75	30	66.16±7.92	23.17±3.38	19.52±5.07	23.47±3.04
	1~2	150	60	65.12±9.16	22.78±3.72	20.05±4.63	22.29±3.51
	3 and above	25	10	69.40±8.24	24.64±2.97	20.84±4.67	23.92±2.69
Sleep conditions	Very good	92	36.8	65.48±9.27	22.83±4.07	19.86±5.14	22.79±3.34
	Good	69	27.6	68.55±8.27	24.11±3.00	20.75±4.73	23.68±2.96
	Poor	69	27.6	63.55±8.66	22.29±3.53	19.22±4.54	22.04±3.81
	Very poor	20	8	66.30±6.16	23.45±2.26	20.35±3.66	22.50±2.40

For peer review only - http://bmjopen.bmj.com/site/about/guidelines.xhtml

Supplemental Table 3 Comparison of health behavior scores of stroke patients with different characteristics

Factors	Group	*N*	%	HBS-SP	Exercise	Medication taking	Instructions	Nutrition	Responsibility	Smoking and alcohol
Gender	Male	136	54.4	56.72±9.07	11.71±4.6	12.00±2.73	8.65±2.48	14.37±4.03	3.30±0.83	6.68±1.59
	Female	114	45.6	56.84±9.01	11.97±5.01	11.42±2.55	9.00±2.70	14.61±3.77	3.19±0.58	6.63±1.82
Marriage	Married	220	88	57.12±8.84	12.04±4.79	11.72±2.55	8.94±2.66	14.60±3.91	3.25±0.74	6.57±1.73
	Divorced	2	0.8	59.50±3.54	12.50±0.71	11.50±0.71	10.00±2.83	15.00±1.41	3.50±0.71	7.00±1.41
	Unmarried	28	11.2	53.86±10.33	10.10±4.86	11.82±3.53	7.93±1.68	13.29±3.80	3.32±0.67	7.39±1.13
Age	＜60	73	29.2	60.47±9.38	13.88±5.14	12.23±2.58	8.60±2.72	15.88±3.83	3.45±1.09	6.42±1.79
	≥60	177	70.8	55.25±8.44	10.99±4.42	11.54±2.67	8.90±2.52	13.90±3.80	3.17±0.49	6.76±1.65
Residential area	Rural	126	50.4	55.83±7.44	11.84±4.69	11.55±2.63	8.84±2.27	13.93±3.40	3.17±0.72	6.49±1.77
	Urban	124	49.6	57.74±10.34	11.82±4.95	11.94±2.68	8.78±2.86	15.04±4.31	3.33±0.74	6.83±1.59
Working state	Unemployed	54	21.6	55.30±8.14	12.43±4.90	11.37±2.53	8.54±2.34	13.67±3.19	3.00±0.00	6.30±1.89
	Pensioner	109	43.6	55.55±8.75	10.81±4.49	11.55±2.56	8.95±2.74	14.23±4.16	3.18±0.49	6.83±1.52
	Working	87	34.8	59.23±9.46	12.75±4.96	12.21±2.82	8.82±2.54	15.30±3.87	3.49±1.06	6.67±1.75
Educational level	Primary and below	115	46	54.56±7.09	11.47±4.47	11.13±2.53	8.69±2.23	13.47±3.27	3.08±0.35	6.72±1.76
	Junior high school	65	27.6	55.14±8.66	11.46±4.06	11.65±2.78	8.83±2.45	13.75±3.96	3.11±0.31	6.34±1.71
	High school	44	17.6	58.80±9.69	10.61±5.11	12.57±2.73	8.39±3.33	16.64±4.37	3.27±0.62	7.32±1.07
	College	14	5.6	63.64±8.72	15.43±3.82	13.07±2.06	9.86±3.08	16.07±3.00	3.57±1.01	5.64±2.27

	University and above	12	4.8	71.50±7.01	17.58±6.42	13.50±1.62	10.25±2.22	18.33±2.53	5.25±1.60	6.58±1.16
Stroke type	Hemorrhagic stroke	6	2.4	64.75±7.94	13.13±6.49	13.13±2.17	11.75±2.05	16.38±2.92	3.00±0.00	7.38±0.91
	Ischemic stroke	244	97.6	56.51±8.95	11.79±4.76	11.69±2.67	8.71±2.54	14.42±3.93	3.26±0.74	6.63±1.71
Family history	Have	25	10	57.68±9.39	13.00±3.81	11.00±2.02	10.16±2.73	13.76±4.15	3.12±0.33	6.64±1.63
	No	255	90	56.68±9.00	11.70±4.90	11.82±2.71	8.66±2.53	14.56±3.88	3.27±0.76	6.66±1.70
Monthly income (RMB)	≤2999	89	35.6	53.47±6.64	10.85±4.20	11.28±2.69	8.58±2.48	13.12±3.46	3.03±0.18	6.60±1.87
	3000-4999	126	50.4	57.63±8.78	12.09±4.89	11.87±2.47	8.94±2.76	14.85±3.84	3.26±0.67	6.63±1.65
	≥5000	35	14	62.11±11.78	13.40±5.54	12.46±3.07	8.94±2.14	16.60±4.08	3.77±1.33	6.94±1.37
Numbers of stroke	1 time	130	52	57.08±9.51	11.77±4.59	11.77±2.73	8.40±2.71	15.00±4.12	3.34±0.87	6.80±1.65
	2 times	74	29.6	57.69±7.11	13.11±4.78	11.58±2.32	9.23±2.41	14.23±3.02	3.18±0.53	6.36±1.72
	3 times	22	8.8	59.32±9.24	12.64±5.48	12.95±2.50	9.82±2.26	14.64±3.17	3.27±0.77	6.00±2.00
	More than 3 times	24	9.6	50.00±8.90	7.50±2.72	10.96±3.13	8.83±2.32	12.29±5.01	3.00±0.00	7.42±1.10
Primary caregiver	Spouse or children	132	52.8	55.58±8.50	10.40±4.48	11.95±2.78	8.63±2.70	14.67±4.31	3.24±0.80	6.69±1.69
	Babysitter or nursing workers	11	4.4	52.36±10.27	9.64±2.87	9.45±2.34	9.18±2.27	12.91±3.36	3.73±0.79	7.45±1.04
	Own	107	42.8	58.71±9.19	13.82±4.66	11.72±2.44	9.00±2.46	14.41±3.40	3.22±0.61	6.54±1.74
Number of chronic	0	75	30	57.47±7.61	12.75±4.69	12.16±2.38	8.92±2.15	14.12±3.11	3.16±0.55	6.36±1.61

diseases										
	1~2	150	60	56.07±9.44	11.35±4.75	11.51±2.84	8.93±2.50	14.34±4.19	3.21±0.61	6.73±1.75
	3 and above	25	10	58.96±10.18	12.00±5.30	11.88±2.20	7.76±3.82	16.40±3.88	3.76±1.42	7.16±1.46
Sleep conditions	Very good	92	36.8	56.11±9.22	11.86±4.79	11.83±2.94	8.68±2.64	13.79±3.91	3.26±0.66	6.68±1.52
	Good	69	27.6	59.35±9.22	12.68±4.70	11.97±2.39	9.23±2.60	15.38±3.88	3.30±0.67	6.78±1.61
	Poor	69	27.6	55.14±9.16	11.12±4.82	11.38±2.63	8.42±2.45	14.45±4.07	3.26±0.94	6.52±2.01
	Very poor	20	8	56.60±4.51	11.25±5.15	11.80±2.33	9.30±2.60	14.65±2.91	3.00±0.00	6.60±1.64

STROBE 2007 (v4) checklist of items to be included in reports of observational studies in epidemiology*

Checklist for cohort, case-control, and cross-sectional studies (combined)

	Item No	Recommendation	Page
**Title and abstract**	1	(*a*) Indicate the study's design with a commonly used term in the title or the abstract	Page 1
		(*b*) Provide in the abstract an informative and balanced summary of what was done and what was found	Page 1
**Introduction**			
Background/rationale	2	Explain the scientific background and rationale for the investigation being reported	Page 1-3
Objectives	3	State specific objectives, including any prespecified hypotheses	Page 3
**Methods**			
Study design	4	Present key elements of study design early in the paper	Page 3
Setting	5	Describe the setting, locations, and relevant dates, including periods of recruitment, exposure, follow-up, and data collection	Page 3
Participants	6	(*a*) Give the eligibility criteria, and the sources and methods of selection of participants	Page 3
Variables	7	Clearly define all outcomes, exposures, predictors, potential confounders, and effect modifiers. Give diagnostic criteria, if applicable	Page 6
Data sources/ measurement	8*	For each variable of interest, give sources of data and details of methods of assessment (measurement). Describe comparability of assessment methods if there is more than one group	Page 4
Bias	9	Describe any efforts to address potential sources of bias	Page 8-9
Study size	10	Explain how the study size was arrived at	Page 3
Quantitative variables	11	Explain how quantitative variables were handled in the analyses. If applicable, describe which groupings were chosen and why	Page 4
Statistical methods	12	(*a*) Describe all statistical methods, including those used to control for confounding	Page 4
		(*b*) Describe any methods used to examine subgroups and interactions	Not applicable
		(*c*) Explain how missing data were addressed	Page 3
		(*d*) If applicable, describe analytical methods taking account of sampling strategy	Not applicable
		(*e*) Describe any sensitivity analyses	Not applicable
**Results**			

For peer review only

Participants	13*	(a) Report numbers of individuals at each stage of study—eg numbers potentially eligible, examined for eligibility, confirmed eligible, included in the study, completing follow-up, and analysed	Not applicable
		(b) Give reasons for non-participation at each stage	Not applicable
		(c) Consider use of a flow diagram	Not applicable
Descriptive data	14*	(a) Give characteristics of study participants (eg demographic, clinical, social) and information on exposures and potential confounders	Page 5
		(b) Indicate number of participants with missing data for each variable of interest	Page 5
Outcome data	15*	Report numbers of outcome events or summary measures	Page 5
Main results	16	(*a*) Give unadjusted estimates and, if applicable, confounder-adjusted estimates and their precision (eg, 95% confidence interval). Make clear which confounders were adjusted for and why they were included	Page 6
		(*b*) Report category boundaries when continuous variables were categorized	Page 5
		(*c*) If relevant, consider translating estimates of relative risk into absolute risk for a meaningful time period	Not applicable
Other analyses	17	Report other analyses done—eg analyses of subgroups and interactions, and sensitivity analyses	Not applicable

For peer review only

**Discussion**			
Key results	18	Summarise key results with reference to study objectives	Page 7
Limitations	19	Discuss limitations of the study, taking into account sources of potential bias or imprecision. Discuss both direction and magnitude of any potential bias	Page 8
Interpretation	20	Give a cautious overall interpretation of results considering objectives, limitations, multiplicity of analyses, results from similar studies, and other relevant evidence	Page 8
Generalisability	21	Discuss the generalisability (external validity) of the study results	Page 8
**Other information**			
Funding	22	Give the source of funding and the role of the funders for the present study and, if applicable, for the original study on which the present article is based	Page 9

*Give information separately for exposed and unexposed groups.

For peer review only

BMJ Open

Mediation role of perceived social support between recurrence risk perception and health behavior among patients with stroke in China: a cross-sectional study

Journal:	*BMJ Open*
Manuscript ID	bmjopen-2023-079812.R2
Article Type:	Original research
Date Submitted by the Author:	05-Dec-2023
Complete List of Authors:	Wang, Xiaoxuan; Zhengzhou University,<br>Zhang, Zhen-xiang; Zhengzhou University,<br>LIN, Bei-lei; Zhengzhou University<br>Jiang, Hu; Zhengzhou University, ;<br>Wang, Wenna; Zhengzhou University,<br>MEI, YONG-XIA; Zhengzhou University, School of Nursing and health<br>Zhang, Chunhui; Nursing and Health school, Zhengzhou University<br>Zhang, Qiushi; Zhengzhou University, Department of Fundmental Nursing<br>CHEN, Su-yan; Zhengzhou University
**Primary Subject Heading**:	Nursing
Secondary Subject Heading:	Nursing
Keywords:	Stroke < NEUROLOGY, Behavior, Chronic Disease

SCHOLARONE™
Manuscripts

I, the Submitting Author has the right to grant and does grant on behalf of all authors of the Work (as defined in the below author licence), an exclusive licence and/or a non-exclusive licence for contributions from authors who are: i) UK Crown employees; ii) where BMJ has agreed a CC-BY licence shall apply, and/or iii) in accordance with the terms applicable for US Federal Government officers or employees acting as part of their official duties; on a worldwide, perpetual, irrevocable, royalty-free basis to BMJ Publishing Group Ltd ("BMJ") its licensees and where the relevant Journal is co-owned by BMJ to the co-owners of the Journal, to publish the Work in this journal and any other BMJ products and to exploit all rights, as set out in our licence.

The Submitting Author accepts and understands that any supply made under these terms is made by BMJ to the Submitting Author unless you are acting as an employee on behalf of your employer or a postgraduate student of an affiliated institution which is paying any applicable article publishing charge ("APC") for Open Access articles. Where the Submitting Author wishes to make the Work available on an Open Access basis (and intends to pay the relevant APC), the terms of reuse of such Open Access shall be governed by a Creative Commons licence – details of these licences and which Creative Commons licence will apply to this Work are set out in our licence referred to above.

Other than as permitted in any relevant BMJ Author's Self Archiving Policies, I confirm this Work has not been accepted for publication elsewhere, is not being considered for publication elsewhere and does not duplicate material already published. I confirm all authors consent to publication of this Work and authorise the granting of this licence.

For peer review only

Mediation role of perceived social support between recurrence risk perception and health behavior among patients with stroke in China: a cross-sectional study

Abstract

Objectives To examine whether stroke patients with high recurrence risk perception would have healthier behavior, and to explore whether perceived social support would function as a mediator.

Design A cross-sectional study.

Setting The study was conducted in a public tertiary hospital in China.

Participants A total of 254 patients with stroke were invited to participate, and 250 patients with stroke completed questionnaires validly.

Primary and secondary outcome measures Questionnaires were administered offline to collect data, consisting of 4 parts: general demographics, and scales related to recurrence risk perception, perceived social support and health behavior. A path analysis and correlation analysis were used to analyse the data.

Results Out of 250 patients with stroke, 78.4% were below the medium level. The majority (70.8%) of these patients were elderly. High recurrence risk perception and high perceived social support were significantly associated with better health behavior (all $P<0.001$). Perceived social support mediated the relationship between recurrence risk perception and health behavior, after controlling for age, gender, education, monthly income in the regression model (95% $CI=[0.263,0.460]$) and the effect value was 0.360. It was also confirmed that perceived social support had a highest mediation effect with a proportion of mediation up to 59.31%.

Conclusions Recurrence risk perception and perceived social support were influential factors in promoting health behavior. Moreover, the impact of recurrence risk perception on health behavior was partially mediated by perceived social support. Therefore, to enhance the sustainability of health behavior, it is crucial to inform patients with stroke about the risk of recurrence. Patients with more perception of recurrence risk can improve their recovery confidence and thus perceive more social support.

Strengths and limitations of this study

- A path analysis is used to analyse the associations among perceived social support, recurrence risk perception and health behavior.
- This study is a cross-sectional survey and therefore cannot make causal inferences.
- Survey data from only one region may limit the generalizability of our findings.

Keywords recurrence risk perception, health behavior, perceived social support, mediation effect, nursing, stroke

Introduction

Globally, stroke recurrence is a significant but preventable risk. A study which contained 500,000 Chinese adults showed that the cumulative recurrence rate for stroke patients was 17% at 1 year, 41% at 5 years, and 53% at 9 years [1]. Despite the implementation of policies and guidelines in various countries aimed at preventing secondary strokes, there has been no significant decrease in the rates of stroke recurrence [2–4]. The occurrence of stroke is closely influenced by a variety of factors, including behavioral, psychological, and social environmental factors. Among these factors, behavioral risk factors plays a significant role in the increasing prevalence of chronic diseases [5].

Adopting healthy lifestyle habits can significantly reduce the risk of stroke recurrence. Specifically, engaging in regular physical activity, following a balanced diet, quitting smoking, and consuming alcohol in

For peer review only - http://bmjopen.bmj.com/site/about/guidelines.xhtml

moderation have been shown to collectively prevent approximately 80% of stroke recurrences [6]. Despite the proven benefits of healthy behavior adoption, the actual rate of implementation remains discouragingly low. Liu et al [7] investigated 515 patients with stroke and found that health behavior level of patients were moderate. In another cross-sectional study[8] involving 112 stroke patients, it was found that only 17% of the participants were adhering to medication regimens. This highlighted the importance of identifying the factors that influence the health behavior of stroke patients and investigating strategies to improve their adherence. Previous studies have shown that various factors, including gender [9], age [10], level of knowledge [11,12], self-efficacy [12,13], recurrence risk perception [14] and social support [12,15] play a significant role in influencing the health behavior of stroke patients.

Recurrence risk perception represents a critical factor influencing the health behavior [14]. Recurrence risk perception refers to the perception of early warning features of recurrence, severity, behavior-related risk factors, and disease-related risk factors [16]. The study concluded that stroke patients' perception of recurrence risk was an important predictor for patients to adopt healthy behaviors to control and reduce the recurrence of stroke [17]. However, the precise relationship between risk perception of recurrence and health behavior actually remains uncertain. According to the health belief model [18], individuals who hold the belief that engaging in risky behaviors carries significant health risks are more likely to adopt healthier behaviors. In other words, recurrence risk perception affects health behavior positively. On the contrary, a cross-sectional comparative study conducted in China showed that patients with stroke who perceived higher risk of recurrence were more likely to adopt healthy behavior [14]. Iversen et al [19] investigated 435 patients with stroke and confirmed that higher seriousness perception of stroke was associated with more help-seeking behavior and shorter prehospital delay. There is also another argument that recurrence risk perception is detrimental to health behavior. Freeman-Gibb et al [20] recruited 117 patients and found patients who perceive a high risk of recurrence always experience higher levels of anxiety and fear. Excessive anxiety and fear were actually detrimental to the patient's adoption of health behavior [21]. Consequently, in order to understand how stroke risk perception affects the likelihood of people adopting healthy behaviors, more research is needed.

Social support is another significant impact factor of health behavior [22]. Social support includes both subjective and objective aspects, and the subjective aspect refers to perception of social support. It is known that social support is an interactive process that involves providing and receiving social support, which means social support must be perceived by patients so that it can be effective [23]. Besides, a systematic review [24] discovered that the associations between perceived social support and health-related quality of life appear to be more frequently significant than the relationships between specific categories or sources of social support and health-related quality of life. Meanwhile, studies [12,15] have indicated that individuals who perceive encouragement, understanding, and assistance from their social circles are more likely to adopt and sustain healthy behaviors such as exercise, proper diet, and adherence to medical treatments.

Perceived social support (PSS) refers to an individual's belief in the availability of various social resources, including emotional support, tangible aid, and informational assistance [25]. The Common Sense Model of self-regulation (CSM) is a theoretical framework which is widely used in the study of illness perception [26]. It was designed to describe dynamic interactions among the variables controlling health behavior in response to current or future health threats [27]. The CSM comprises six main components [28]: (1) situational stimuli, (2) cognitive illness representations, (3) emotional illness representations, (4) coping strategies, (5) illness and emotional outcomes, and (6) coping appraisal. Previous studies have already applied the CSM model to predict health outcomes in diabetes [29], explore the experience of breathlessness [30] and predict healthy eating among individuals at risk of metabolic syndrome [29]. For patients with stroke, the surrounding situational stimuli (including endogenous or social factors) cause patients with stroke to develop cognitive and emotional illness representation (recurrence risk perception). Cognitive and emotional illness representation are also continually updated [28] in response to memories of new information (e.g., perceived social support), which affects coping strategies (the choice of adopting health behavior). Therefore, it is

For peer review only - http://bmjopen.bmj.com/site/about/guidelines.xhtml

reasonable to choose the CSM model as the theoretical framework for our study.

According to the CSM, we speculated the effect of recurrence risk perception on health behavior was partly mediated by perceived social support. Nevertheless, there is a significant gap in the knowledge regarding the impact of an individual's perception of stroke recurrence risk on their sense of social support and subsequent emotional consequence [31]. In fact, in traditional Chinese philosophy, the concepts of 'recurrence' and 'risk' are often met with fear and apprehension. Stroke patients may experience avoidance or isolation behavior as a result of their fear, subsequently resulting in a decline in their perceived social support networks [32], ultimately reducing the perceived level of social support for stroke patients. Therefore, we hypothesized that recurrence risk perception affects the perceived social support directly and negatively. Moreover, patients with higher recurrence risk perception may be prevented from adopting healthy behavior due to the negative emotions. It proposes the following hypothesis (Figure 1). This study aimed to examine the relationship between the recurrence risk perception and health behavior among patients with stroke and explore the role of perceived social support in this relationship.

Hypothesis 1 – Recurrence risk perception affects health behavior directly and negatively.
Hypothesis 2 – Recurrence risk perception affects the perceived social support directly and negatively.
Hypothesis 3 – Perceived social support affects health behavior directly and positively.
Hypothesis 4– Recurrence risk perception indirectly affects health behavior via Perceived social support

Insert Figure 1 here.

Methods

Participants and procedure

Convenience sampling was used to conduct this cross-sectional survey from June to August, 2021. Inpatient patients with stroke were selected from the department of neurology at a tertiary hospital in Luoyang City, Henan Province, China. The following were the inclusion criteria: (a) patients were diagnosed with stroke by MRI or CT [33]; (b) during recovery stage (2 weeks to 6 months after onset [34]); (c) having the ability to communicate with researchers verbally; (d) over 18-year-old. Exclusion criteria: (a) combined with severe cardiac, liver, and renal dysfunction or other malignant tumors; (b) a history of serious mental illness or a family history of serious mental illness. Kendall [35] proposed that the sample size should be at least 5-10 times the independent variables. There were 16 variables in this study (13 general information questions, the recurrence risk perception of stroke scale, the perceived social support scale, and the health behavior scale for patients with stroke). Considering 20% of invalid questionnaires, the minimum sample size of the study was 200 patients with stroke; hence, a sample size of 250 met the requirement for testing the hypothesized models.

Data collection

To ensure the validity and reliability of our study, we first conducted a pre-test with a small sample of stroke patients. We selected five patients with primary school education and five elderly individuals to participate in this pre-test phase. Our research assistants carefully reviewed the questionnaires completed by these participants and identified any questions that were unclear or required further explanation. We then made necessary revisions to ensure that all participants would understand the questions accurately during the formal investigation.

Measures

The demographic questionnaire

The demographic and disease-specific data questionnaire was designed and used to collect the information of

For peer review only - http://bmjopen.bmj.com/site/about/guidelines.xhtml

age, gender, marriage, residential area, educational level, working state, stroke type, family history, number of strokes, monthly income, primary caregiver, sleep conditions, and number of chronic diseases.

The stroke recurrence risk perception scale（SRRPS）

The stroke recurrence risk perception scale is a tool used to assess stroke patients' recurrence risk perception of their disease. Our research group [16] developed the scale in 2022, which includes two sections with 20 items. The first section contains three items that assess recurrence risk perception of patients with stroke. The second section contains 17 items in three domains: perceived illness risk factors, perceived behavioral risk factors, and perceived severity. There are four items in the “perceived illness risk factors” dimension which includes patients' perception of illness risk factors such as hyperlipidemia, hyperglycemia, hypertension, and atrial fibrillation of stroke recurrence. There are six items in the “perceived behavioral risk factors” dimension, which refers to patients' perception of behavioral risk factors such as vegetables, fruits, and salt intake, smoking and alcohol consumption, and exercise to prevent stroke recurrence. There are seven items in the “perceived severity” dimension, which means patients' perceptions of harm from recurrence on daily activities, cognition, mood, level of impairment, and number of recurrences. Higher scores indicate greater risk awareness, and the score for this section is calculated independently of the overall score. The second section of items ranges from 1-3 (disagree, not clear, agree). The Cronbach's alpha of the second section is 0.850 and the Cronbach's alpha for each dimension are 0.875, 0.815, and 0.804. This scale was applied by Tang in 236 patients with first-episode ischemic stroke patients in China and the Cronbach's alpha for the second part was 0.890 [36]. The validity of this scale has also been verified by validation factor analysis in another study [37]. In this study, we used the second section of the scale to examine stroke patients' perception of recurrence risk.

The perceived social support scale (PSSS)

The perceived social support scale is an instrument to measure the individual's self-perception of multi-dimensional social support. The scale was developed by Zimet in 1987 and revised into a Chinese version by Jiang [38]in 1999. It includes three dimensions of family support, friend support, and other support (relatives, leaders, colleagues, etc.), with 12 items. Each dimension has 4 items. The elements in the various dimensions indicate whether the individual requested assistance from others (families, friends, relatives, etc.) and whether others were able to assist in a timely manner. It is a 7-point Likert scale (from 1 = extremely uncompliant to 7 =extremely compliant). The total score of PSSS ranges from 12-84 points, scores ranging from 12 to 36 points indicate low perceived support, 37 to 60 points indicate intermediate perceived support, and 61 to 84 points indicate high perceived support. The Cronbach's alpha value of PSSS is 0.92. This scale has been applied to stroke patients in other studies [39,40].

The health behavior scale for stroke patients (HBS-SP)

The health behavior scale for stroke patients is a scale to assess the level of health behavior in patients with stroke. The scale was developed by Wan et al [41] in 2017. It includes 6 dimensions and 25 items. The exercise dimension contains six items, it refers to duration, frequency, type, intensity, plans, and motivation of exercise. There are 4 items in the medication taking dimension, which means knowledge of taking medication and compliance. The instructions dimension (4 items) mainly includes adherence to physician's orders for moderate intake of salt, sugar, oil, etc. The responsibility dimension contains three items, which includes paying attention to ingredient lists on food package labels and monitoring heart rate during exercise. The nutrition dimension includes six items, which means eating appropriate amounts of meat, eggs, milk, grains, fruits, soy products, etc. There are 2 items in the smoking and alcohol dimension, which inplies appropriate levels of smoking and alcohol consumption. It is a 4-point Likert scale (from 1 = never to 4 = routinely performed). But the items of medication and tobacco, and alcohol were reversed. Higher scores indicate better

For peer review only - http://bmjopen.bmj.com/site/about/guidelines.xhtml

health behavior. An average score of 2.5 points (between "sometimes" and "often") is considered an intermediate level of health behavior. The Cronbach's alpha value of HBS-SP scale is 0.878.

Statistical analysis

The Statistical Package of Social Science software version 26 (IBM Corp. Released, 2017) was used to analyze the data. Participants' social demographics characteristics and other related variables were examined by descriptive statistics of frequencies and percentages. Pearson correlation analysis was used to analyze the interrelationships between the three variables. We applied the testing procedure recommended by Wen [42] and the mediation model was analyzed using Model 4 in the PROCESS Marco [43]. For the best test of the mediation effect, we carried out the bootstrapping procedure to measure the indirect effect and 95% confidence interval (*CI*) were estimated. $P<0.05$ was considered statistically significant. A 95% confidence interval excluding zero was regarded as statistically significant.

Patient and public involvement

Patients or the public were not involved in the design, conduct, reporting or dissemination of our research.

Results

General Demographics

A total of 254 questionnaires were obtained. However, 4 invalid questionnaires were removed because the questionnaires with a completion rate of less than 95%. Finally, the number of effective participants was 250, and the final effective rate of questionnaire collection was 98.4%. Over half (70.8%) of patients were elderly patients (≥60 years). Less than half of the patients in the sample had a primary school degree or less (46.1%). Moreover, 34.8% were working and a majority (97.6%) of patients with stroke were ischemic stroke. In addition, 50.4% of stroke patients were low and middle income group. 52% of them were first-episode patients. Comparison of the scales' scores of stroke patients with different characteristics are shown in Supplemental Table.

Descriptive statistics of the SRRPS, PSSS and HBS-SP

Table 1 displayed mean scores, standard deviations and ranges of SRRPS, PSSS and HBS-SP. The mean and SD of stroke recurrence risk perception scale were 41.04±8.64 scores. The mean and SD of perceived social support scale were 65.86±8.78 scores. The mean and SD of perceived social support scale were 56.78±9.03 scores. Therefore, among the patients with stroke we recruited, recurrence risk perception and perceived social support were at high levels. Health behavior was at a moderately low level.

Table 1 Scores on the SRRPS, PSSS and HBS-SP

Variables	Items	Range of score	Mean	Stand deviation
SRRPS	17	17~51	41.04	8.64
perceived illness risk factors	4	6~18	19.96	4.76
perceived behavioral risk factors	6	4~12	22.80	3.35
perceived severity	7	7~21	17.84	4.05
PSSS	12	40~80	65.86	8.78
family support	4	13~28	23.08	3.58
friend support	4	7~28	19.97	4.77
other support	4	12~28	22.80	3.35
HBS-SP	25	28~81	56.78	9.03

For peer review only - http://bmjopen.bmj.com/site/about/guidelines.xhtml

exercise	6	5~24	11.83	4.81
medication taking	4	5~19	11.74	2.66
instructions	4	4~17	8.81	2.58
responsibility	3	3~8	3.25	3.91
nutrition	6	6~24	14.48	1.69
smoking and alcohol	2	2~8	6.66	0.73

Abbreviations: SRRPS, stroke recurrence risk perception scale; PSSS, Perceived social support scale; HBS-SP, Health behavior scale for stroke patients

Correlation Analysis between SRRPS, PSSS and HBS-SP

Pearson's correlation analysis was used to investigate the relationships among recurrence risk perception, health behavior, and perceived social support in patients with stroke. Briefly, recurrence risk perception was positively correlated with health behavior (r=0.679, $P<0.001$), perceived social support was positively correlated with health behavior (r=0.778, $P<0.001$), recurrence risk perception was positively correlated with perceived social support (r=0.639, $P<0.001$), meaning that recurrence risk perception, health behavior, and perceived social support were positively correlated with each other in patients with stroke.

Mediation Analysis of SRRPS, PSSS and HBS-SP

Building on our initial findings from the correlation analysis, we next performed mediation analysis to explore the potential association underlying the relationship between an individual's perceived social support, their perception of stroke recurrence risk, and their adoption of healthy behaviors among stroke patients. We controlled for some general demographic factors (like age, gender, monthly income, education) which are considered as important predictors affect health behavior in patients with stroke in China. In model 1, recurrence risk perception had a positive predictive effect on health behavior (β= 0.607, $P<0.001$). In model 2, recurrence risk perception had a positive predictive effect on perceived social support (β= 0.604, $P<0.001$). In model 3, recurrence risk perception and perceived social support positively predicted health behavior (β= 0.247, β= 0.596, $P<0.001$), but the comparison of the effect coefficients showed that the effect of recurrence risk perception on health behavior was diminished from 0.607 to 0.247, which meant that perceived social support partially mediated the effect between recurrence risk perception and health behavior, as shown in Table 2.

Table 2 Mediation results for recurrence risk perception predicting health behavior mediated by perceived social support

Recurrence risk perception → Health behavior				
	SE	β	t	P
Age	0.968	-1.246	-1.287	0.200
Gender	0.863	0.958	1.110	0.268
Education	0.460	1.187	2.579	0.011
Monthly Income	0.742	1.158	1.561	0.120
Recurrence risk perception	0.053	0.607	11.529	<0.001
Recurrence risk perception →Perceived social support				
Gender	0.890	0.912	1.026	0.306
Education	0.474	1.669	3.522	0.005
Monthly Income	0.764	-1.514	-1.981	0.049

For peer review only - http://bmjopen.bmj.com/site/about/guidelines.xhtml

Recurrence risk perception	0.054	0.604	11.137	<0.001
Recurrence risk perception, Perceived social support → Health behavior				
Age	0.766	-1.389	-1.812	0.071
Gender	0.685	0.415	0.607	0.545
Education	0.373	0.193	0.516	0.606
Monthly Income	0.592	2.059	3.480	0.006
Recurrence risk perception	0.051	0.247	4.833	<0.001
Perceived social support	0.049	0.596	12.110	<0.001

To ensure the accuracy of the test, this study further used the bootstrap method [43] to test the mediating effect of perceived social support between recurrence risk perception and health behavior with 5000 replicate samples. The results are shown in Table 3, and the 95% confidence interval of the mediating effect of perceived social support was [0.26,0.46], which did not contain zero, indicating that the mediating effect of perceived social support in the process of recurrence risk perception affecting health behavior was established, and the effect accounted for 59.31% (Table 3). The mediating model of perceived social support is constructed in Figure 2.

Table 3 Mediating model examination by bootstrap

Recurrence risk perception → Health behavior				
	Effect	SE	LL 95%*CI*	UL 95%*CI*
Indirect effect	0.360	0.050	0.263	0.460
Direct effect	0.247	0.512	0.147	0.348

Abbreviations: SE, standard error; LL 95% CI, Lower Limit of 95% confidence interval; UL 95%CI, Upper Limit of 95% confidence interval

Insert Figure 2 here.

Discussion

We found that 78.4% of patients with stroke scored less than 2.5 on the health behavior scale, which meant they had moderately low health behavior. This finding was consistent with previous studies. Li et al [44] investigated 462 patients with stroke in three tertiary hospitals in Liaoning Province, China, and found that patients with stroke had moderate health behavior. The same results were reaffirmed in another study [45]. More than half of the 231 patients with stroke had moderately low health behavior. This highlighted the urgent need for further research and focus on the influencing factors of healthy behavior among stroke patients.

This study demonstrated that perceived social support was positively correlated with health behavior. This finding was consistent with our hypothesis 3 and previous studies [46,47]. A cross-sectional study [48] enrolled 133 hypertensive stroke patients at 6 months after discharge in China has shown the perceptions of chronic illness resources which receive from health care team, family and friends, and community were positively correlated with health behavior. Tan [49] conducted a cross-sectional study among 350 stroke participants in China and found perceived social support had a direct positively effect on rehabilitation motivation. Moreover, Bandura's social theory [50] also proposed that factors such as social support were actual aspects of the incidence of behavior. Brouwer-Goossensen has found social support to be a positive

For peer review only - http://bmjopen.bmj.com/site/about/guidelines.xhtml

factor in health behavior change in interviews with 18 stroke patients [51]. Our study provided further evidence of the relationship between perceived social support and health behavior. Therefore, perceived social support is an important factor in improving health behavior in patients with stroke. In addition, there was a significant positive correlation between recurrence risk perception and perceived social support. As a result, our initial hypothesis 2 was contradicted by the findings of our study. In other words, recurrence risk perception affects the perceived social support directly and positively actually. We speculated the reason for this result was that the higher stroke patients' recurrence risk perception, the more control they had over their diseases. This sense of control over the disease could partly increase their self-efficacy [52] to stay in a good mind, promoting a positive mindset, and concurrently enhancing their perception of social support. Calderon et al [53] discovered that a majority of chronic patients expressed a desire for increased information and greater involvement in the decision-making process. To the best of our knowledge, our study was the first study to investigate the association between stroke patients' perceptions of recurrence and their perceived social support in China.

Most importantly, we found that recurrence risk perception appeared to influence health behavior via two pathways, a direct pathway and an indirect pathway mediated by perceived social support. (1) First pathway, recurrence risk perception was correlated with health behavior significantly and positively. This was contrary to our research hypothesis 1. It may be due to the fact that stroke patients did not view the risk of recurrence as a frightening event. They gradually increased their confidence in their own recovery as they acquired more knowledge about the risk instead. In this way, they were better able to cope with the disease. For example, they began to adopt healthy behaviors to prevent recurrence. In fact, this findings were consistent with the health belief model (HBM) [18]. Besides, our previous research found patients with stroke who lacked a perception of the risk of recurrence and preventive strategies exhibited a tendency to limit their regular hospital visits or refrain from adopting healthy behaviors aimed at preventing recurrence [40]. In other words, recurrence risk perception was an important positive factor of health behavior. (2) The second pathway was that recurrence risk perception mediated the effect of perceived social support on health behavior. This was also contrary to our research hypothesis 4. To effectively implement secondary stroke prevention measures, it is crucial for patients with stroke to have a comprehensive understanding of the risk factors associated with recurrent stroke. Their understanding of the lifestyle changes needed to reduce the chance of future recurrences is equally crucial [54]. In patients with stroke, the perceived risk of recurrence involves not only just concern and fear, but also the understanding of early warning signs and risk factors associated with stroke recurrence. So patients with stroke with a higher recurrence risk perception also demonstrated a greater depth of stroke knowledge [55], and these patients showed a higher level of confidence in recovery than patients with a lower perceived risk of recurrence. Therefore, they perceive information or emotional support from those around them better. The presence of these social supports played a significant role in improving their emotional well-being, consequently fostering a greater willingness to embrace healthier behaviors.

Overall, our study found that recurrence risk perception not only directly and positively influenced health behaviors of patients with stroke, but also further influenced health behavior by positively affecting perceived social support. This was consistent with the connotation of the CSM model [26]. In other words, our findings provided practical support for the CSM model. As the practical implication is that our finding may aid future research in identifying entry points for health behavior interventions by taking the methods that improve stroke patients' perception of recurrence risk and perceived social support. For example, health care providers could consider providing patients with recurrence risk communication. Specifically, healthcare professionals can help patients to have an accurate understanding of the risk of recurrence. First, a comparison between the subjective and objective risk perception scores for stroke recurrence is necessary for healthcare professionals. If the stroke recurrence risk perception and objective risk perception scores are inconsistent, then risk communication should be initiated with stroke patients [56]. Common risk communication tools mainly include graphical [57] and numerical forms [58]. To enable patients correctly grasp their rates of recurrence,

For peer review only - http://bmjopen.bmj.com/site/about/guidelines.xhtml

appropriate risk communication strategies should be chosen according to their various level of education and preferences. In addition, healthcare professionals can provide stroke patients with the necessary knowledge and skills to prevent recurrence, such as the aura of stroke recurrence, risk factors of recurrence, and prognosis of recurrence. By doing this, stroke patients' perceptions of social support can be enhanced, which will ultimately help them adopt healthy behaviors. It can also boost their confidence in their ability to recover.

The study still has some limitations. Firstly, it employed a cross-sectional design, thereby precluding the establishment of a causal relationship between recurrence risk perception and health behavior. Nonetheless, the study successfully confirmed a significant correlation between recurrence risk perception and health behavior. Secondly, despite our efforts to incorporate multi-center sampling, the impact of COVID-19 necessitated the restriction of our study to a single tertiary hospital in Henan Province. Therefore, the findings may not be generalizable to all patients with stroke. Besides, only six hemorrhagic patients with stroke were included in our study, so it was difficult for us to explore differences in stroke type in patients' perceived risk of recurrence, perceived social support and health behavior. Lastly, our study included a relatively elderly population of patients with stroke, which may have some impact on the generalizability of the findings. Therefore, we recommend the design of a multi-center longitudinal study specifically focusing on young stroke patients in future research. Such a study design would help clarify the dynamic trends in recurrence risk perception, perceived social support, and health behavior over time.

Conclusion

In conclusion, this study found that perceived social support mediated the relationship between recurrence risk perception and health behavior. Our findings expand upon previous research on the interplay between recurrence risk perception, perceived social support, and health behavior in patients with stroke. The results of this study can serve as a useful guide for future clinical nursing practice and research in this area. Considering the importance of recurrence risk perception and perceived social support, it is recommended that these factors be incorporated into the assessment process for patients with stroke, both during hospitalization and follow-up care. We suggest that healthcare professionals should assist patients with stroke in developing an accurate perception of recurrence risk with appropriate recurrence risk communication approaches. This method can significantly improve patients' capacity to identify and access social support, ultimately enhancing their confidence in recovery and motivation to engage in healthy behaviors. Moreover, it is crucial to recognize the significance of social support from family and friends as a primary source of perceived social support. Healthcare professionals should encourage and educate patients' loved ones to offer emotional support, which can positively impact patients' recovery and well-being.

Acknowledgements

We would like to acknowledge the tertiary hospital of Henan province for their cooperation. We greatly appreciate the support of all patients for their voluntary participation in this study.

Contributors

ZZX and LBL designed the study. WXX, JH and WWN contributed to writing and revising. WXX, MYX and ZCH were in charge of data collection and analysis. ZQS and CSY were in charge of study design and essential help. All authors read and approved the final manuscript.

Funding

This work was supported by the National Natural Science Foundation of China Youth Science Foundation Project (72104221).

For peer review only - http://bmjopen.bmj.com/site/about/guidelines.xhtml

Competing interests

None declared.

Patient consent for publication

Each participant gave informed written consent and permission to publish before analysis of the study.

Ethics approval

The Research Ethics Committee of Zhengzhou University (ZZURIB2020-08) approved the study.

Provenance and peer review

Not commissioned; externally peer reviewed.

Data availability statement

Data are available upon reasonable request.

For peer review only

References

1 Chen Y, Wright N, Guo Y, *et al.* Mortality and recurrent vascular events after first incident stroke: a 9-year community-based study of 0·5 million Chinese adults. *Lancet Glob Health*. 2020;8:e580–90.

2 Gladstone DJ, Lindsay MP, Douketis J, *et al.* Canadian Stroke Best Practice Recommendations: Secondary Prevention of Stroke Update 2020. *Can J Neurol Sci*. 2022;49:315–37.

3 Kleindorfer DO, Towfighi A, Chaturvedi S, *et al.* 2021 Guideline for the Prevention of Stroke in Patients With Stroke and Transient Ischemic Attack: A Guideline From the American Heart Association/American Stroke Association. *Stroke*. 2021;52:e364–467.

4 Liu L, Chen W, Zhou H, *et al.* Chinese Stroke Association guidelines for clinical management of cerebrovascular disorders: executive summary and 2019 update of clinical management of ischaemic cerebrovascular diseases. *Stroke Vasc Neurol*. 2020;5:159–76.

5 O'Donnell MJ, Xavier D, Liu L, *et al.* Risk factors for ischaemic and intracerebral haemorrhagic stroke in 22 countries (the INTERSTROKE study): a case-control study. *Lancet*. 2010;376:112–23.

6 Abdullahi SU, Sunusi S, Abba MS, *et al.* Hydroxyurea for secondary stroke prevention in children with sickle cell anemia in Nigeria: a randomized controlled trial. *Blood*. 2023;141:825–34.

7 Liu Y, Wei M, Guo L, *et al.* Association between illness perception and health behaviour among stroke patients: The mediation effect of coping style. *Journal of Advanced Nursing*. 2021;77:2307–18.

8 Mugwano I, Kaddumukasa M, Mugenyi L, *et al.* Poor drug adherence and lack of awareness of hypertension among hypertensive stroke patients in Kampala, Uganda: a cross sectional study. *BMC Res Notes*. 2016;9:3.

9 Wu X, Min L, Cong L, *et al.* Sex differences in health-related quality of life among adult stroke patients in Northeastern China. *J Clin Neurosci*. 2014;21:957–61.

10 Kim G-G, Chae D-H, Park M-S, *et al.* Factors Influencing 1-Year Medication Adherence of Korean Ischemic Stroke Survivors. *Int J Behav Med*. 2020;27:225–34.

11 Parappilly BP, Field TS, Mortenson WB, *et al.* Determinants Influencing the Prestroke Health Behaviors and Cardiovascular Disease Risk of Stroke Patients: A Cross-Sectional Study. *J Stroke Cerebrovasc Dis*. 2019;28:1509–18.

12 Santos M, Kövari E, Gold G, *et al.* The neuroanatomical model of post-stroke depression: towards a change of focus? *J Neurol Sci*. 2009;283:158–62.

For peer review only - http://bmjopen.bmj.com/site/about/guidelines.xhtml

13 Brouwer-Goossensen D, Genugten L van, Lingsma H, *et al.* Determinants of intention to change health-related behavior and actual change in patients with TIA or minor ischemic stroke. *Patient Educ Couns*. 2016;99:644–50.

14 Ren H, Guo Y-F, Zhang Z-X, *et al.* Perception of recurrent risk versus objective measured risk of ischemic stroke in first-ever stroke patients from a rural area in China: A cross-sectional study. *Patient Educ Couns*. 2023;107:107586.

15 Holder C, Krishnamurthi R, Theadom A. Exploring facilitators and barriers to long-term behavior change following health-wellness coaching for stroke prevention: A qualitative study conducted in Auckland, New Zealand. *Brain Behav*. 2023;13:e2671.

16 Lin B, Zhang Z, Guo Y. Development and psychometric test of Recurrence Risk Perception Scale for Patients with Stroke. *Chinese Journal of Nursing*. 2022;56:1666–71.

17 Thakkar J, Heeley EL, Chalmers J, *et al.* Inaccurate risk perceptions contribute to treatment gaps in secondary prevention of cardiovascular disease. *Intern Med J*. 2016;46:339–46.

18 Champion VL, Skinner CS. The health belief model. *Health behavior and health education: Theory, research, and practice, 4th ed*. San Francisco, CA, US: Jossey-Bass 2008:45–65.

19 Iversen AB, Blauenfeldt RA, Johnsen SP, *et al.* Understanding the seriousness of a stroke is essential for appropriate help-seeking and early arrival at a stroke centre: A cross-sectional study of stroke patients and their bystanders. *Eur Stroke J*. 2020;5:351–61.

20 Freeman-Gibb LA, Janz NK, Katapodi MC, *et al.* The relationship between illness representations, risk perception and fear of cancer recurrence in breast cancer survivors. *Psychooncology*. 2017;26:1270–7. doi: 10.1002/pon.4143

21 Lee-Jones C, Humphris G, Dixon R, *et al.* Fear of cancer recurrence--a literature review and proposed cognitive formulation to explain exacerbation of recurrence fears. *Psychooncology*. 1997;6:95–105. doi: 10.1002/(SICI)1099-1611(199706)6:2<95::AID-PON250>3.0.CO;2-B

22 Blanton S, Scheibe DC, Rutledge AH, *et al.* Family-Centered Care During Constraint-Induced Therapy After Chronic Stroke: A Feasibility Study. *Rehabil Nurs*. 2019;44:349–57.

23 Northcott S, Moss B, Harrison K, *et al.* A systematic review of the impact of stroke on social support and social networks: associated factors and patterns of change. *Clin Rehabil*. 2016;30:811–31.

24 Kruithof WJ, van Mierlo ML, Visser-Meily JMA, *et al.* Associations between social support and stroke survivors' health-related quality of life--a systematic review. *Patient Educ Couns*. 2013;93:169–76.

25 Dahlan R, Ghazal E, Saltaji H, *et al.* Impact of social support on oral health among immigrants and ethnic minorities: A systematic review. *PLoS One*. 2019;14:e0218678.

26 Leventhal H, Phillips LA, Burns E. The Common-Sense Model of Self-Regulation (CSM): a dynamic framework for understanding illness self-management. *J Behav Med*. 2016;39:935–46. doi: 10.1007/s10865-016-9782-2

27 Leventhal H, Diefenbach M, Leventhal E. Illness cognition: Using common sense to understand treatment adherence and affect cognition interactions. *Cognitive Therapy and Research*. 1992;16:143–63.

28 Hagger MS, Orbell S. The common sense model of illness self-regulation: a conceptual review and proposed extended model. *Health Psychol Rev*. 2022;16:347–77.

29 Fall E, Chakroun-Baggioni N, Böhme P, *et al.* Common sense model of self-regulation for understanding adherence and quality of life in type 2 diabetes with structural equation modeling. *Patient Educ Couns*. 2021;104:171–8. doi: 10.1016/j.pec.2020.06.023

30 Johnston KN, Burgess R, Kochovska S, *et al.* Exploring the Experience of Breathlessness with the Common-Sense Model of Self-Regulation (CSM). *Healthcare (Basel)*. 2023;11:1686.

31 Adamson M, Cotoc C, Choi K, *et al.* Cancer Communication Outside of the Physician-Patient Relationship: The Experience of Communicating and Understanding the Meaning of Prognosis. *Am J Hosp Palliat Care*. 2019;36:711–7.

32 Singh T, Newby DE. Is the fear of disease worse than the disease itself? *Heart*. 2021;107:91–2.

33 Neurology CS of, Society CS. Diagnostic criteria of cerebrovascular diseases in China (version 2019). *Chinese Journal of Neurology*. 2019;52:710–5. doi: 10.3760/cma.j.issn.1006-7876.2019.09.003

34 Gao C, Wu C, Zhao J, *et al.* Guidelines for the Diagnosis and Treatment of Cerebral Infarction in China (2017). *Chinese Journal of Integrated Traditional and Western Medicine*. 2018;38:136–44.

For peer review only

For peer review only - http://bmjopen.bmj.com/site/about/guidelines.xhtml

35 Ni P, Chen J, Liu N. Sample size estimation in quantitative nursing research. *Chinese Journal of Nursing*. 2010;45:378–80.

36 Tang B, Lv Y, Wang X, *et al.* Analysis of status and influencing factors of recurrence risk perception in first-episode ischemic stroke patients. *Chinese Journal of Nursing*. 2023;58:289–95.

37 Gao F, An Q, Liu D, *et al.* Recurrence risk perception and its correlates in stroke patients. *Journal of Nursing(China)*. 2023;38:30–3.

38 Wang X, Wang X, Ma H. *Manual of the Mental Health Rating Scale*. Beijing: Chinese Journal of Mental Health 1999.

39 Dong X, Yang S, Guo Y, *et al.* Exploring Psychoneurological Symptom Clusters in Acute Stroke Patients: A Latent Class Analysis. *J Pain Res*. 2022;15:789–99.

40 Wang X, Shang S, Yang H, *et al.* Associations of psychological distress with positive psychological variables and activities of daily living among stroke patients: a cross-sectional study. *BMC Psychiatry*. 2019;19:381.

41 Wan L, Xiong X, Pan J, *et al.* Development and reliability as well as validity test of Health Behavior Scale for stroke patients. *Journal of Nursing Science*. 2017;32:25–9.

42 Wen Z. Analyses of Mediating Effects: The Development of Methods and Models. *Advances in psychological Science*. 2014;22:731–45.

43 Henderson AR. The bootstrap: a technique for data-driven statistics. Using computer-intensive analyses to explore experimental data. *Clin Chim Acta*. 2005;359:1–26.

44 Li S, He H, Li H, *et al.* Chain Mediating Effects of Executive Intention of Rehabilitation Exercise Behaviors and Mental Health Literacy between Perceived Social Support and Health Behaviors among Stroke Inpatients. *Military Nursing*. 2023;40:34–8.

45 Liu M, Liang F, Yin Y, *et al.* Latent Profile Analysis of Health Literacy and Comparison of Health Behavior Differences in Patients with Stroke. *Military Nursing*. 2023;40:52-55+70.

46 Denche-Zamorano A, Pisà-Canyelles J, Barrios-Fernandez S, *et al.* Is Psychological Distress Associated with Self-Perceived Health, Perceived Social Support and Physical Activity Level in Spanish Adults with Diabetes? *J Pers Med*. 2023;13:739.

47 Guo A, Jin H, Mao J, *et al.* Impact of health literacy and social support on medication adherence in patients with hypertension: a cross-sectional community-based study. *BMC Cardiovasc Disord*. 2023;23:93. doi: 10.1186/s12872-023-03117-x

48 Wen K, Shen M-J, Deng C-S, *et al.* Association between chronic illness resources and health behaviors in hypertensive stroke patients at 6 months after discharge: a cross-sectional study. *Ann Transl Med*. 2022;10:460.

49 Tan M, Li H, Wang X. Analysis of the current status of rehabilitation motivation and its influencing factors in older adults with stroke: a cross-sectional study. *Front Aging Neurosci*. 2023;15:1186681.

50 Bandura A. Social cognitive theory: an agentic perspective. *Annu Rev Psychol*. 2001;52:1–26.

51 Brouwer-Goossensen D, den Hertog HM, Mastenbroek-de Jong MA, *et al.* Patient perspectives on health-related behavior change after transient ischemic attack or ischemic stroke. *Brain Behav*. 2021;11:e01993. doi: 10.1002/brb3.1993

52 Al Shafaee MA, Ganguly SS, Al Asmi AR. Perception of stroke and knowledge of potential risk factors among Omani patients at increased risk for stroke. *BMC Neurol*. 2006;6:38.

53 Calderon C, Jiménez-Fonseca P, Ferrando PJ, *et al.* Psychometric properties of the Shared Decision-Making Questionnaire (SDM-Q-9) in oncology practice. *Int J Clin Health Psychol*. 2018;18:143–51.

54 Hiraga A. Perception of recurrent stroke risk among stroke survivors. *Neuroepidemiology*. 2011;37:88–9.

55 Slark J, Bentley P, Majeed A, *et al.* Awareness of stroke symptomatology and cardiovascular risk factors amongst stroke survivors. *J Stroke Cerebrovasc Dis*. 2012;21:358–62.

56 Chen DW, Reyes-Gastelum D, Wallner LP, *et al.* Disparities in risk perception of thyroid cancer recurrence and death. *Cancer*. 2020;126:1512–21. doi: 10.1002/cncr.32670

57 Goodyear-Smith F, Arroll B, Chan L, *et al.* Patients Prefer Pictures to Numbers to Express Cardiovascular Benefit From Treatment. *The Annals of Family Medicine*. 2008;6:213–7.

58 Fair AKI, Murray PG, Thomas A, *et al.* Using hypothetical data to assess the effect of numerical format and context on

For peer review only - http://bmjopen.bmj.com/site/about/guidelines.xhtml

the perception of coronary heart disease risk. *American Journal of Health Promotion*. 2008;22:291–6.

Figure 1 The hypothesis model of recurrence risk perception, health behavior and perceived social support in patients with stroke

Figure 2 Mediating effects of perceived social support on the relationship between recurrence risk perception and health behavior (*P < 0.001)

For peer review only

For peer review only - http://bmjopen.bmj.com/site/about/guidelines.xhtml

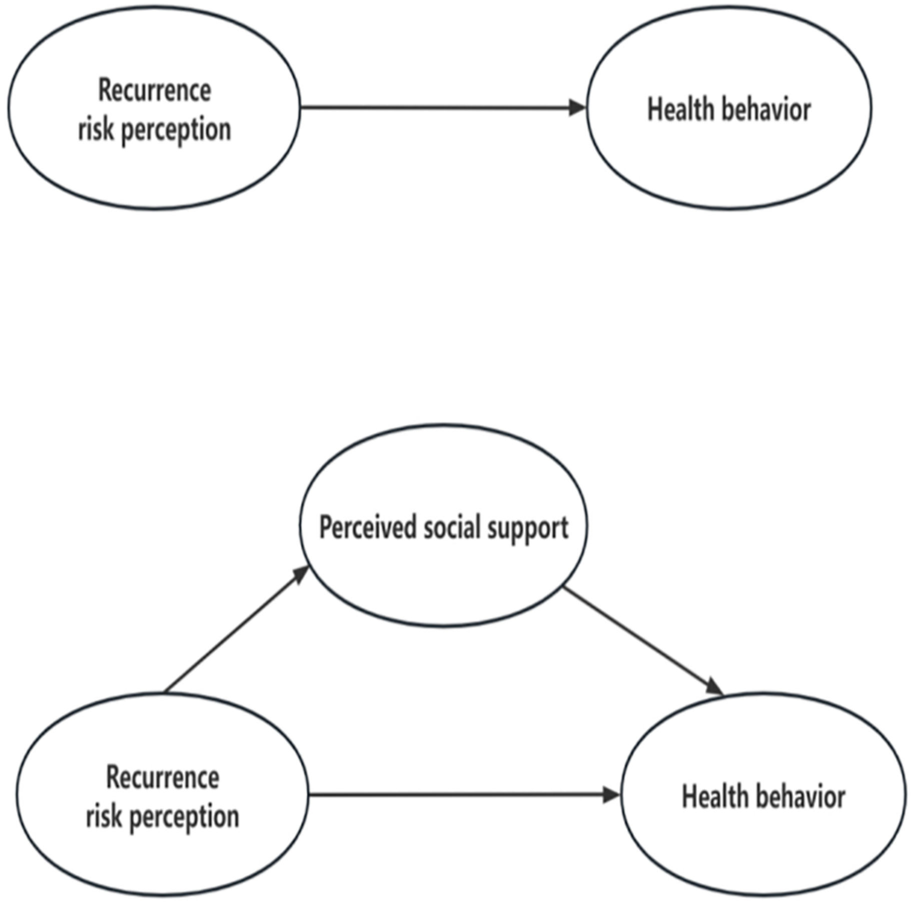

90x90mm (300 x 300 DPI)

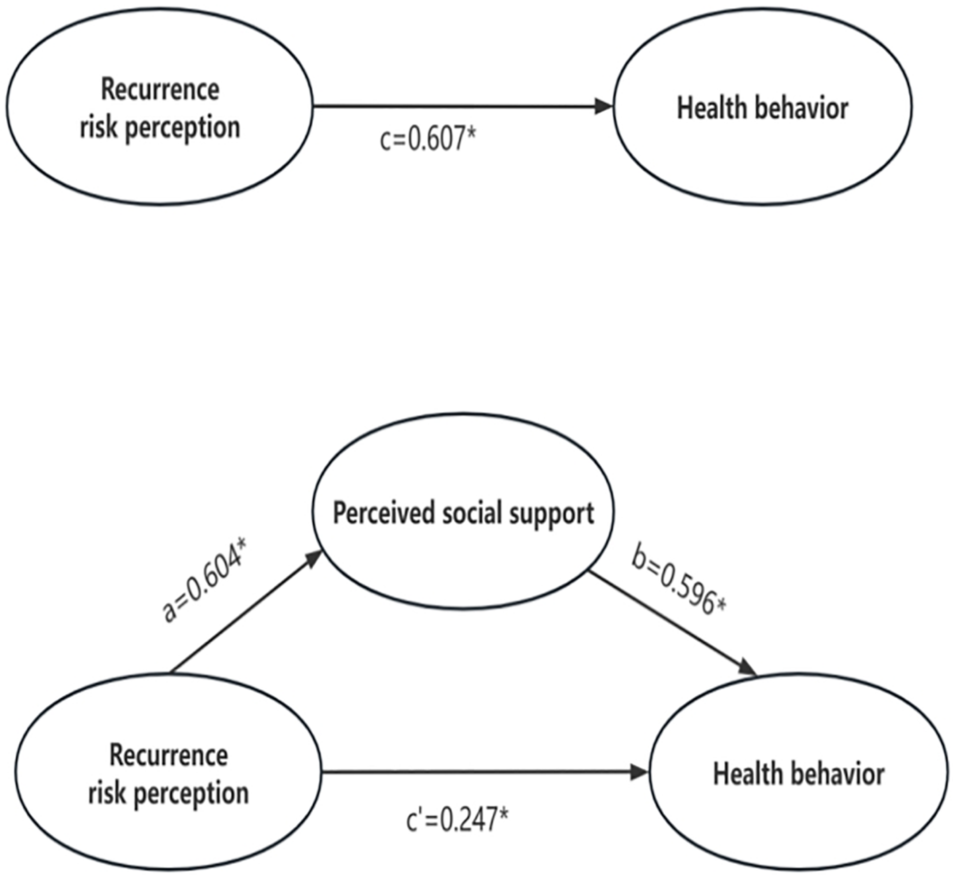

90x90mm (500 x 500 DPI)

Supplemental Table 1 Comparison of recurrence risk perception scores of stroke patients with different characteristics

Factors	Group	*N*	%	SRRPS	Perceived illness risk factors	Perceived behavioral risk factors	Perceived severity
Gender	Male	136	54.4	40.88±9.73	14.68±4.03	8.71±2.38	17.43±4.45
	Female	114	45.6	41.23±7.15	14.66±3.60	8.24±2.30	18.33±3.48
Marriage	Married	220	88	41.46±8.23	14.92±3.71	8.60±2.29	17.95±3.85
	Divorced	2	0.8	47.00±0.00	17.00±1.41	9.50±2.12	20.50±0.71
	Unmarried	28	11.2	37.29±10.91	12.50±4.24	7.71±2.79	16.82±5.40
Age	<60	73	29.2	44.55±6.44	15.93±2.83	9.59±2.06	19.03±2.93
	≥60	177	70.8	39.59±9.02	14.15±4.07	8.04±2.32	17.84±4.05
Residential area	Rural	126	50.4	39.92±7.91	14.08±3.91	8.10±2.17	17.68±3.79
	Urban	124	49.6	42.17±9.21	15.27±3.68	8.89±2.46	18.01±4.32
Working state	Unemployed	54	21.6	40.11±7.29	13.48±3.30	7.85±2.28	18.78±3.66
	Pensioner	109	43.6	39.37±9.98	14.16±4.22	8.18±2.47	17.03±4.66
	Working	87	34.8	43.70±6.85	16.06±3.22	9.28±2.04	18.29±3.23
Educational level	Primary and below	115	46	38.72±7.99	13.56±3.97	7.82±2.06	17.29±4.06
	Junior high school	65	27.6	40.49±8.61	14.75±3.35	8.22±2.28	17.52±4.28
	High school	44	17.6	42.81±9.35	15.50±3.99	9.11±2.46	18.20±4.18
	College	14	5.6	48.64±2.21	18.00±0.00	10.57±1.60	20.07±2.06
	University and above	12	4.8	50.75±0.45	18.00±0.00	11.75±0.45	21.00±0.00
Stroke type	Hemorrhagic stroke	6	2.4	47.50±3.74	17.25±1.04	10.00±2.14	20.25±1.04
	Ischemic stroke	244	97.6	40.82±8.67	14.59±3.86	8.44±2.34	17.76±4.09

Family history	Have	25	10	38.36±10.60	13.32±4.96	8.08±2.33	16.96±4.33
	No	255	90	41.33±8.37	14.82±3.67	8.54±2.35	17.94±4.02
Monthly income (RMB)	≤2999	89	35.6	37.62±7.98	13.08±3.81	7.56±2.00	16.90±4.22
	3000-4999	126	50.4	42.59±8.39	15.52±3.64	8.75±2.27	18.32±3.90
	≥5000	35	14	44.14±8.56	15.69±3.31	9.91±2.56	18.54±3.85
Numbers of stroke	1 time	130	52	41.54±8.19	15.38±3.42	8.78±2.05	17.38±3.99
	2 times	74	29.6	43.15±6.12	15.04±3.18	8.88±2.16	19.23±2.70
	3 times	22	8.8	42.27±8.14	14.27±4.08	8.27±3.01	19.41±3.70
	More than 3 times	24	9.6	30.67±11.07	10.08±4.57	5.92±2.30	14.67±5.73
Primary caregiver	Spouse or children	132	52.8	17.30±4.46	14.30±4.16	8.17±2.42	39.82±9.54
	Babysitter or nursing workers	11	4.4	38.00±11.12	14.73±4.47	14.73±4.47	15.73±5.10
	Own	107	42.8	42.85±6.71	15.12±3.31	8.99±2.22	18.74±3.15
Number of chronic diseases	0	75	30	40.15±8.96	14.07±3.80	8.15±2.15	17.93±3.94
	1~2	150	60	41.29±9.17	14.75±4.04	8.67±2.43	17.83±4.17
	3 and above	25	10	42.20±6.87	16.04±1.86	8.48±2.40	17.68±3.87
Sleep conditions	Very good	92	36.8	40.07±9.91	14.12±4.20	8.35±2.55	17.52±4.60
	Good	69	27.6	42.51±8.25	15.25±3.78	8.81±2.37	18.45±3.47
	Poor	69	27.6	39.65±7.86	14.23±3.61	8.28±2.28	17.14±4.21
	Very poor	20	8	45.20±2.19	16.75±1.55	8.80±1.32	19.65±0.88

Supplemental Table 2 Comparison of perceived social support scores of stroke patients with different characteristics

Factors	Group	*N*	%	PSSS	Family support	Friend support	Other support
Gender	Male	136	54.4	65.50±9.07	22.65±3.89	20.43±4.32	22.42±3.48
	Female	114	45.6	66.29±8.43	23.60±3.11	19.41±5.22	23.28±3.15
Marriage	Married	220	88	66.27±8.55	23.17±3.49	20.15±4.84	22.95±3.15
	Divorced	2	0.8	69.00±12.73	22.50±3.54	23.00±4.24	23.50±4.95
	Unmarried	28	11.2	62.43±9.88	22.39±4.26	18.36±3.96	21.68±4.60
Gender	＜60	73	29.2	68.64±8.56	23.90±2.92	21.11±5.21	23.63±2.74
	≥60	177	70.8	64.71±8.63	22.75±3.78	19.50±4.51	22.47±3.52
Residential area	Rural	126	50.4	65.54±8.14	23.23±3.08	19.37±4.90	22.94±3.31
	Urban	124	49.6	66.19±9.40	22.94±4.04	20.58±4.57	22.67±3.40
Working state	Unemployed	54	21.6	64.33±8.70	22.67±3.39	19.57±4.96	22.09±3.54
	Pensioner	109	43.6	65.01±8.81	22.76±3.82	19.71±4.78	22.54±3.34
	Working	87	34.8	67.87±8.51	23.75±3.32	20.54±4.64	23.59±3.12
Educational level	Primary and below	115	46	64.03±7.79	22.57±3.31	18.92±4.90	22.53±3.06
	Junior high school	65	27.6	64.07±8.83	22.32±3.79	19.77±4.17	21.97±3.82
	High school	44	17.6	68.37±8.74	23.80±3.57	20.95±4.73	23.61±3.19
	College	14	5.6	71.36±7.96	25.36±3.03	21.29±3.95	24.71±3.20
	University and above	12	4.8	77.58±4.54	26.83±1.57	25.92±.151	25.83±2.04
Stroke type	Hemorrhagic stroke	6	2.4	73.88±7.38	26.50±1.60	21.63±5.58	25.75±1.16
	Ischemic stroke	244	97.6	65.60±8.71	22.97±3.57	19.91±4.74	22.71±3.36
Family history	Have	25	10	66.00±8.56	23.28±2.57	19.24±5.33	23.49±3.38
	No	255	90	65.84±8.82	23.06±3.68	20.05±4.71	22.73±3.35
Monthly income (RMB)	≤2999	89	35.6	64.73±8.07	22.79±3.48	19.10±4.46	33.84±3.44

	3000-4999	126	50.4	65.67±8.59	23.09±3.46	19.94±5.01	22.65±3.08
	≥5000	35	14	69.40±10.38	23.83±4.22	22.23±3.92	23.29±4.06
Numbers of stroke	1 time	130	52	65.88±9.05	22.90±3.62	20.18±4.84	22.80±3.35
	2 times	74	29.6	67.03±7.36	23.88±2.91	20.18±4.63	22.97±2.93
	3 times	22	8.8	67.27±9.19	23.59±3.65	20.32±4.47	23.36±3.47
	More than 3 times	24	9.6	60.83±9.63	21.17±4.45	17.83±4.82	21.83±4.34
Primary caregiver	Spouse or children	132	52.8	64.95±8.77	23.23±3.77	18.86±5.03	22.86±3.54
	Babysitter or nursing workers	11	4.4	58.73±8.00	19.64±3.80	19.55±1.51	19.55±4.25
	Own	107	42.8	67.71±8.37	23.25±3.15	21.37±4.29	23.08±2.83
Number of chronic diseases	0	75	30	66.16±7.92	23.17±3.38	19.52±5.07	23.47±3.04
	1~2	150	60	65.12±9.16	22.78±3.72	20.05±4.63	22.29±3.51
	3 and above	25	10	69.40±8.24	24.64±2.97	20.84±4.67	23.92±2.69
Sleep conditions	Very good	92	36.8	65.48±9.27	22.83±4.07	19.86±5.14	22.79±3.34
	Good	69	27.6	68.55±8.27	24.11±3.00	20.75±4.73	23.68±2.96
	Poor	69	27.6	63.55±8.66	22.29±3.53	19.22±4.54	22.04±3.81
	Very poor	20	8	66.30±6.16	23.45±2.26	20.35±3.66	22.50±2.40

Supplemental Table 3 Comparison of health behavior scores of stroke patients with different characteristics

Factors	Group	*N*	%	HBS-SP	Exercise	Medication taking	Instructions	Nutrition	Responsibility	Smoking and alcohol
Gender	Male	136	54.4	56.72±9.07	11.71±4.6	12.00±2.73	8.65±2.48	14.37±4.03	3.30±0.83	6.68±1.59
	Female	114	45.6	56.84±9.01	11.97±5.01	11.42±2.55	9.00±2.70	14.61±3.77	3.19±0.58	6.63±1.82
Marriage	Married	220	88	57.12±8.84	12.04±4.79	11.72±2.55	8.94±2.66	14.60±3.91	3.25±0.74	6.57±1.73
	Divorced	2	0.8	59.50±3.54	12.50±0.71	11.50±0.71	10.00±2.83	15.00±1.41	3.50±0.71	7.00±1.41
	Unmarried	28	11.2	53.86±10.33	10.10±4.86	11.82±3.53	7.93±1.68	13.29±3.80	3.32±0.67	7.39±1.13
Age	＜60	73	29.2	60.47±9.38	13.88±5.14	12.23±2.58	8.60±2.72	15.88±3.83	3.45±1.09	6.42±1.79
	≥60	177	70.8	55.25±8.44	10.99±4.42	11.54±2.67	8.90±2.52	13.90±3.80	3.17±0.49	6.76±1.65
Residential area	Rural	126	50.4	55.83±7.44	11.84±4.69	11.55±2.63	8.84±2.27	13.93±3.40	3.17±0.72	6.49±1.77
	Urban	124	49.6	57.74±10.34	11.82±4.95	11.94±2.68	8.78±2.86	15.04±4.31	3.33±0.74	6.83±1.59
Working state	Unemployed	54	21.6	55.30±8.14	12.43±4.90	11.37±2.53	8.54±2.34	13.67±3.19	3.00±0.00	6.30±1.89
	Pensioner	109	43.6	55.55±8.75	10.81±4.49	11.55±2.56	8.95±2.74	14.23±4.16	3.18±0.49	6.83±1.52
	Working	87	34.8	59.23±9.46	12.75±4.96	12.21±2.82	8.82±2.54	15.30±3.87	3.49±1.06	6.67±1.75
Educational level	Primary and below	115	46	54.56±7.09	11.47±4.47	11.13±2.53	8.69±2.23	13.47±3.27	3.08±0.35	6.72±1.76
	Junior high school	65	27.6	55.14±8.66	11.46±4.06	11.65±2.78	8.83±2.45	13.75±3.96	3.11±0.31	6.34±1.71
	High school	44	17.6	58.80±9.69	10.61±5.11	12.57±2.73	8.39±3.33	16.64±4.37	3.27±0.62	7.32±1.07
	College	14	5.6	63.64±8.72	15.43±3.82	13.07±2.06	9.86±3.08	16.07±3.00	3.57±1.01	5.64±2.27

	University and above	12	4.8	71.50±7.01	17.58±6.42	13.50±1.62	10.25±2.22	18.33±2.53	5.25±1.60	6.58±1.16
Stroke type	Hemorrhagic stroke	6	2.4	64.75±7.94	13.13±6.49	13.13±2.17	11.75±2.05	16.38±2.92	3.00±0.00	7.38±0.91
	Ischemic stroke	244	97.6	56.51±8.95	11.79±4.76	11.69±2.67	8.71±2.54	14.42±3.93	3.26±0.74	6.63±1.71
Family history	Have	25	10	57.68±9.39	13.00±3.81	11.00±2.02	10.16±2.73	13.76±4.15	3.12±0.33	6.64±1.63
	No	255	90	56.68±9.00	11.70±4.90	11.82±2.71	8.66±2.53	14.56±3.88	3.27±0.76	6.66±1.70
Monthly income (RMB)	≤2999	89	35.6	53.47±6.64	10.85±4.20	11.28±2.69	8.58±2.48	13.12±3.46	3.03±0.18	6.60±1.87
	3000-4999	126	50.4	57.63±8.78	12.09±4.89	11.87±2.47	8.94±2.76	14.85±3.84	3.26±0.67	6.63±1.65
	≥5000	35	14	62.11±11.78	13.40±5.54	12.46±3.07	8.94±2.14	16.60±4.08	3.77±1.33	6.94±1.37
Numbers of stroke	1 time	130	52	57.08±9.51	11.77±4.59	11.77±2.73	8.40±2.71	15.00±4.12	3.34±0.87	6.80±1.65
	2 times	74	29.6	57.69±7.11	13.11±4.78	11.58±2.32	9.23±2.41	14.23±3.02	3.18±0.53	6.36±1.72
	3 times	22	8.8	59.32±9.24	12.64±5.48	12.95±2.50	9.82±2.26	14.64±3.17	3.27±0.77	6.00±2.00
	More than 3 times	24	9.6	50.00±8.90	7.50±2.72	10.96±3.13	8.83±2.32	12.29±5.01	3.00±0.00	7.42±1.10
Primary caregiver	Spouse or children	132	52.8	55.58±8.50	10.40±4.48	11.95±2.78	8.63±2.70	14.67±4.31	3.24±0.80	6.69±1.69
	Babysitter or nursing workers	11	4.4	52.36±10.27	9.64±2.87	9.45±2.34	9.18±2.27	12.91±3.36	3.73±0.79	7.45±1.04
	Own	107	42.8	58.71±9.19	13.82±4.66	11.72±2.44	9.00±2.46	14.41±3.40	3.22±0.61	6.54±1.74
Number of chronic	0	75	30	57.47±7.61	12.75±4.69	12.16±2.38	8.92±2.15	14.12±3.11	3.16±0.55	6.36±1.61

diseases										
	1~2	150	60	56.07±9.44	11.35±4.75	11.51±2.84	8.93±2.50	14.34±4.19	3.21±0.61	6.73±1.75
	3 and above	25	10	58.96±10.18	12.00±5.30	11.88±2.20	7.76±3.82	16.40±3.88	3.76±1.42	7.16±1.46
Sleep conditions	Very good	92	36.8	56.11±9.22	11.86±4.79	11.83±2.94	8.68±2.64	13.79±3.91	3.26±0.66	6.68±1.52
	Good	69	27.6	59.35±9.22	12.68±4.70	11.97±2.39	9.23±2.60	15.38±3.88	3.30±0.67	6.78±1.61
	Poor	69	27.6	55.14±9.16	11.12±4.82	11.38±2.63	8.42±2.45	14.45±4.07	3.26±0.94	6.52±2.01
	Very poor	20	8	56.60±4.51	11.25±5.15	11.80±2.33	9.30±2.60	14.65±2.91	3.00±0.00	6.60±1.64

For peer review only

*STROBE 2007 (v4) checklist of items to be included in reports of observational studies in epidemiology**

Checklist for cohort, case-control, and cross-sectional studies (combined)

	Item No	Recommendation	Page
**Title and abstract**	1	(*a*) Indicate the study's design with a commonly used term in the title or the abstract	Page 1
		(*b*) Provide in the abstract an informative and balanced summary of what was done and what was found	Page 1
**Introduction**			
Background/rationale	2	Explain the scientific background and rationale for the investigation being reported	Page 1-3
Objectives	3	State specific objectives, including any prespecified hypotheses	Page 3
**Methods**			
Study design	4	Present key elements of study design early in the paper	Page 3
Setting	5	Describe the setting, locations, and relevant dates, including periods of recruitment, exposure, follow-up, and data collection	Page 3
Participants	6	(*a*) Give the eligibility criteria, and the sources and methods of selection of participants	Page 3
Variables	7	Clearly define all outcomes, exposures, predictors, potential confounders, and effect modifiers. Give diagnostic criteria, if applicable	Page 6
Data sources/ measurement	8*	For each variable of interest, give sources of data and details of methods of assessment (measurement). Describe comparability of assessment methods if there is more than one group	Page 4
Bias	9	Describe any efforts to address potential sources of bias	Page 8-9
Study size	10	Explain how the study size was arrived at	Page 3
Quantitative variables	11	Explain how quantitative variables were handled in the analyses. If applicable, describe which groupings were chosen and why	Page 4
Statistical methods	12	(*a*) Describe all statistical methods, including those used to control for confounding	Page 4
		(*b*) Describe any methods used to examine subgroups and interactions	Not applicable
		(*c*) Explain how missing data were addressed	Page 3
		(*d*) If applicable, describe analytical methods taking account of sampling strategy	Not applicable
		(*e*) Describe any sensitivity analyses	Not applicable
**Results**			

For peer review only

For peer review only - http://bmjopen.bmj.com/site/about/guidelines.xhtml

Participants	13*	(a) Report numbers of individuals at each stage of study—eg numbers potentially eligible, examined for eligibility, confirmed eligible, included in the study, completing follow-up, and analysed	Not applicable
		(b) Give reasons for non-participation at each stage	Not applicable
		(c) Consider use of a flow diagram	Not applicable
Descriptive data	14*	(a) Give characteristics of study participants (eg demographic, clinical, social) and information on exposures and potential confounders	Page 5
		(b) Indicate number of participants with missing data for each variable of interest	Page 5
Outcome data	15*	Report numbers of outcome events or summary measures	Page 5
Main results	16	(*a*) Give unadjusted estimates and, if applicable, confounder-adjusted estimates and their precision (eg, 95% confidence interval). Make clear which confounders were adjusted for and why they were included	Page 6
		(*b*) Report category boundaries when continuous variables were categorized	Page 5
		(*c*) If relevant, consider translating estimates of relative risk into absolute risk for a meaningful time period	Not applicable
Other analyses	17	Report other analyses done—eg analyses of subgroups and interactions, and sensitivity analyses	Not applicable

For peer review only

**Discussion**			
Key results	18	Summarise key results with reference to study objectives	Page 7
Limitations	19	Discuss limitations of the study, taking into account sources of potential bias or imprecision. Discuss both direction and magnitude of any potential bias	Page 8
Interpretation	20	Give a cautious overall interpretation of results considering objectives, limitations, multiplicity of analyses, results from similar studies, and other relevant evidence	Page 8
Generalisability	21	Discuss the generalisability (external validity) of the study results	Page 8
**Other information**			
Funding	22	Give the source of funding and the role of the funders for the present study and, if applicable, for the original study on which the present article is based	Page 9

*Give information separately for exposed and unexposed groups.

For peer review only

BMJ Open

Mediation role of perceived social support between recurrence risk perception and health behavior among patients with stroke in China: a cross-sectional study

Journal:	*BMJ Open*
Manuscript ID	bmjopen-2023-079812.R3
Article Type:	Original research
Date Submitted by the Author:	20-Dec-2023
Complete List of Authors:	Wang, Xiaoxuan; Zhengzhou University,<br>Zhang, Zhen-xiang; Zhengzhou University,<br>LIN, Bei-lei; Zhengzhou University<br>Jiang, Hu; Zhengzhou University, ;<br>Wang, Wenna; Zhengzhou University,<br>MEI, YONG-XIA; Zhengzhou University, School of Nursing and health<br>Zhang, Chunhui; Nursing and Health school, Zhengzhou University<br>Zhang, Qiushi; Zhengzhou University, Department of Fundmental Nursing<br>CHEN, Su-yan; Zhengzhou University
**Primary Subject Heading**:	Nursing
Secondary Subject Heading:	Nursing
Keywords:	Stroke < NEUROLOGY, Behavior, Chronic Disease

SCHOLARONE™
Manuscripts

I, the Submitting Author has the right to grant and does grant on behalf of all authors of the Work (as defined in the below author licence), an exclusive licence and/or a non-exclusive licence for contributions from authors who are: i) UK Crown employees; ii) where BMJ has agreed a CC-BY licence shall apply, and/or iii) in accordance with the terms applicable for US Federal Government officers or employees acting as part of their official duties; on a worldwide, perpetual, irrevocable, royalty-free basis to BMJ Publishing Group Ltd ("BMJ") its licensees and where the relevant Journal is co-owned by BMJ to the co-owners of the Journal, to publish the Work in this journal and any other BMJ products and to exploit all rights, as set out in our licence.

The Submitting Author accepts and understands that any supply made under these terms is made by BMJ to the Submitting Author unless you are acting as an employee on behalf of your employer or a postgraduate student of an affiliated institution which is paying any applicable article publishing charge ("APC") for Open Access articles. Where the Submitting Author wishes to make the Work available on an Open Access basis (and intends to pay the relevant APC), the terms of reuse of such Open Access shall be governed by a Creative Commons licence – details of these licences and which Creative Commons licence will apply to this Work are set out in our licence referred to above.

Other than as permitted in any relevant BMJ Author's Self Archiving Policies, I confirm this Work has not been accepted for publication elsewhere, is not being considered for publication elsewhere and does not duplicate material already published. I confirm all authors consent to publication of this Work and authorise the granting of this licence.

For peer review only

Mediation role of perceived social support between recurrence risk perception and health behavior among patients with stroke in China: a cross-sectional study

Abstract

Objectives To examine whether stroke patients with high recurrence risk perception would have healthier behavior, and to explore whether perceived social support would function as a mediator.

Design A cross-sectional study.

Setting The study was conducted in a public tertiary hospital in China.

Participants A total of 254 patients with stroke were invited to participate, and 250 patients with stroke completed questionnaires validly.

Primary and secondary outcome measures Questionnaires were administered offline to collect data, consisting of 4 parts: general demographics, and scales related to recurrence risk perception, perceived social support, and health behavior. A path analysis and correlation analysis were used to analyse the data.

Results Out of 250 patients with stroke, 78.4% had moderately low health behavior. The majority (70.8%) of these patients were elderly. High recurrence risk perception and high perceived social support were significantly associated with better health behavior (all $P<0.001$). Perceived social support mediated the relationship between recurrence risk perception and health behavior after controlling for age, gender, education, and monthly income in the regression model (95% CI=[0.263,0.460]) and the effect value was 0.360. It was also confirmed that perceived social support had the highest mediation effect with a proportion of mediation up to 59.31%.

Conclusions Recurrence risk perception and perceived social support were influential factors in promoting health behavior. Moreover, the impact of recurrence risk perception on health behavior was partially mediated by perceived social support. Therefore, to enhance the sustainability of health behavior, it is crucial to inform patients with stroke about the risk of recurrence. Patients with more perception of recurrence risk can improve their recovery confidence and thus perceive more social support.

Strengths and limitations of this study

- A path analysis is used to analyse the associations among perceived social support, recurrence risk perception, and health behavior.
- This study is a cross-sectional survey and therefore cannot make causal inferences.
- Survey data from only one region may limit the generalizability of our findings.

Keywords recurrence risk perception, health behavior, perceived social support, mediation effect, nursing, stroke

Introduction

Globally, stroke recurrence is a significant but preventable risk. A study that contained 500,000 Chinese adults showed that the cumulative recurrence rate for stroke patients was 17% at 1 year, 41% at 5 years, and 53% at 9 years [1]. Despite the implementation of policies and guidelines in various countries aimed at preventing secondary strokes, there has been no significant decrease in the rates of stroke recurrence [2–4]. The occurrence of stroke is closely influenced by a variety of factors, including behavioral, psychological, and social-environmental factors. Among these factors, behavioral risk factors play a significant role in the increasing prevalence of chronic diseases [5].

Adopting healthy lifestyle habits can significantly reduce the risk of stroke recurrence. Specifically,

For peer review only - http://bmjopen.bmj.com/site/about/guidelines.xhtml

engaging in regular physical activity, following a balanced diet, quitting smoking, and consuming alcohol in moderation have been shown to collectively prevent approximately 80% of stroke recurrences [6]. Despite the proven benefits of healthy behavior adoption, the actual rate of implementation remains discouragingly low. Liu et al [7] investigated 515 patients with stroke and found that the health behavior level of patients was moderate. In another cross-sectional study[8] involving 112 stroke patients, it was found that only 17% of the participants were adhering to medication regimens. This highlighted the importance of identifying the factors that influence the health behavior of stroke patients and investigating strategies to improve their adherence. Previous studies have shown that various factors, including gender [9], age [10], level of knowledge [11,12], self-efficacy [12,13], recurrence risk perception [14], and social support [12,15] play a significant role in influencing the health behavior of stroke patients.

Recurrence risk perception represents a critical factor influencing health behavior [14]. Recurrence risk perception refers to the perception of early warning features of recurrence, severity, behavior-related risk factors, and disease-related risk factors [16]. The study concluded that stroke patients' perception of recurrence risk was an important predictor for patients to adopt healthy behaviors to control and reduce the recurrence of stroke [17]. However, the precise relationship between risk perception of recurrence and health behavior remains uncertain. According to the health belief model [18], individuals who hold the belief that engaging in risky behaviors carries significant health risks are more likely to adopt healthier behaviors. In other words, recurrence risk perception affects health behavior positively. On the contrary, a cross-sectional comparative study conducted in China showed that patients with stroke who perceived a higher risk of recurrence were more likely to adopt healthy behavior [14]. Iversen et al [19] investigated 435 patients with stroke and confirmed that a higher seriousness perception of stroke was associated with more help-seeking behavior and shorter prehospital delay. There is also another argument that recurrence risk perception is detrimental to health behavior. Freeman-Gibb et al [20] recruited 117 patients and found patients who perceive a high risk of recurrence always experience higher levels of anxiety and fear. Excessive anxiety and fear were actually detrimental to the patient's adoption of health behavior [21]. Consequently, in order to understand how stroke risk perception affects the likelihood of people adopting healthy behaviors, more research is needed.

Social support is another significant impact factor on health behavior [22]. Social support includes both subjective and objective aspects, and the subjective aspect refers to the perception of social support. It is known that social support is an interactive process that involves providing and receiving social support, which means social support must be perceived by patients so that it can be effective [23]. Besides, a systematic review [24] discovered that the associations between perceived social support and health-related quality of life appear to be more frequently significant than the relationships between specific categories or sources of social support and health-related quality of life. Meanwhile, studies [12,15] have indicated that individuals who perceive encouragement, understanding, and assistance from their social circles are more likely to adopt and sustain healthy behaviors such as exercise, proper diet, and adherence to medical treatments.

Perceived social support (PSS) refers to an individual's belief in the availability of various social resources, including emotional support, tangible aid, and informational assistance [25]. The Common Sense Model of Self-regulation (CSM) is a theoretical framework that is widely used in the study of illness perception [26]. It was designed to describe dynamic interactions among the variables controlling health behavior in response to current or future health threats [27]. The CSM comprises six main components [28]: (1) situational stimuli, (2) cognitive illness representations, (3) emotional illness representations, (4) coping strategies, (5) illness and emotional outcomes, and (6) coping appraisal. Previous studies have already applied the CSM model to predict health outcomes in diabetes [29], explore the experience of breathlessness [30], and predict healthy eating among individuals at risk of metabolic syndrome [29]. For patients with stroke, the surrounding situational stimuli (including endogenous or social factors) cause patients with stroke to develop cognitive and emotional illness representation (recurrence risk perception). Cognitive and emotional illness representation are also continually updated [28] in response to memories of new information (e.g., perceived

For peer review only - http://bmjopen.bmj.com/site/about/guidelines.xhtml

social support), which affects coping strategies (the choice of adopting health behavior). Therefore, it is reasonable to choose the CSM model as the theoretical framework for our study.

According to the CSM, we speculated the effect of recurrence risk perception on health behavior was partly mediated by perceived social support. Nevertheless, there is a significant gap in the knowledge regarding the impact of an individual's perception of stroke recurrence risk on their sense of social support and subsequent emotional consequences [31]. In fact, in traditional Chinese philosophy, the concepts of 'recurrence' and 'risk' are often met with fear and apprehension. Stroke patients may experience avoidance or isolation behavior as a result of their fear, subsequently resulting in a decline in their perceived social support networks [32], ultimately reducing the perceived level of social support for stroke patients. Therefore, we hypothesized that recurrence risk perception will be negatively and significantly related to perceived social support. Moreover, patients with higher recurrence risk perception may be prevented from adopting healthy behavior due to negative emotions. It proposes the following hypothesis (Figure 1). This study aimed to examine the relationship between the recurrence risk perception and health behavior among patients with stroke and explore the role of perceived social support in this relationship.

Hypothesis 1 – Recurrence risk perception is negatively correlated with health behavior.

Hypothesis 2 – Recurrence risk perception is negatively associated with perceived social support.

Hypothesis 3 – Perceived social support positively and significantly relates to health behavior.

Hypothesis 4– Perceived social support partially mediates the relationships between recurrence risk perception and health behavior.

Insert Figure 1 here.

Methods

Participants and procedure

Convenience sampling was used to conduct this cross-sectional survey from June to August, 2021. Inpatient patients with stroke were selected from the department of neurology at a tertiary hospital in Luoyang City, Henan Province, China. The following were the inclusion criteria: (a) patients were diagnosed with stroke by MRI or CT [33]; (b) during the recovery stage (2 weeks to 6 months after onset [34]); (c) having the ability to communicate with researchers verbally; (d) over 18-year-old. Exclusion criteria: (a) combined with severe cardiac, liver, and renal dysfunction or other malignant tumors; (b) a history of serious mental illness or a family history of serious mental illness. Kendall [35] proposed that the sample size should be at least 5-10 times the independent variables. There were 16 variables in this study (13 general information questions, the recurrence risk perception of stroke scale, the perceived social support scale, and the health behavior scale for patients with stroke). Considering 20% of invalid questionnaires, the minimum sample size of the study was 200 patients with stroke; hence, a sample size of 250 met the requirement for testing the hypothesized models.

Data collection

To ensure the validity and reliability of our study, we first conducted a pre-test with a small sample of stroke patients. We selected five patients with primary school education and five elderly individuals to participate in this pre-test phase. Our research assistants carefully reviewed the questionnaires completed by these participants and identified any questions that were unclear or required further explanation. We then made necessary revisions to ensure that all participants would understand the questions accurately during the formal investigation.

Measures

For peer review only - http://bmjopen.bmj.com/site/about/guidelines.xhtml

The demographic questionnaire

The demographic and disease-specific data questionnaire was designed and used to collect information of age, gender, marriage, residential area, educational level, working state, stroke type, family history, number of strokes, monthly income, primary caregiver, sleep conditions, and number of chronic diseases.

The stroke recurrence risk perception scale（SRRPS）

The stroke recurrence risk perception scale is a tool used to assess stroke patients' recurrence risk perception of their disease. Our research group [16] developed the scale in 2022, which includes two sections with 20 items. The first section contains three items that assess the recurrence risk perception of patients with stroke. The second section contains 17 items in three domains: perceived illness risk factors, perceived behavioral risk factors, and perceived severity. There are four items in the “perceived illness risk factors” dimension which includes patients' perception of illness risk factors such as hyperlipidemia, hyperglycemia, hypertension, and atrial fibrillation of stroke recurrence. There are six items in the “perceived behavioral risk factors” dimension, which refers to patients' perception of behavioral risk factors such as vegetables, fruits, and salt intake, smoking, and alcohol consumption, and exercise to prevent stroke recurrence. There are seven items in the “perceived severity” dimension, which means patients' perceptions of harm from recurrence on daily activities, cognition, mood, level of impairment, and number of recurrences. Higher scores indicate greater risk awareness, and the score for this section is calculated independently of the overall score. The second section of items ranges from 1-3 (disagree, not clear, agree). The Cronbach's alpha of the second section is 0.850 and the Cronbach's alpha for each dimension are 0.875, 0.815, and 0.804. This scale was applied by Tang in 236 patients with first-episode ischemic stroke patients in China and the Cronbach's alpha for the second part was 0.890 [36]. The validity of this scale has also been verified by validation factor analysis in another study [37]. In this study, we used the second section of the scale to examine stroke patients' perception of recurrence risk.

The perceived social support scale (PSSS)

The perceived social support scale is an instrument to measure the individual's self-perception of multi-dimensional social support. The scale was developed by Zimet in 1987 and revised into a Chinese version by Jiang [38]in 1999. It includes three dimensions of family support, friend support, and other support (relatives, leaders, colleagues, etc.), with 12 items. Each dimension has 4 items. The elements in the various dimensions indicate whether the individual requested assistance from others (families, friends, relatives, etc.) and whether others were able to assist promptly. It is a 7-point Likert scale (from 1 = extremely uncompliant to 7 =extremely compliant). The total score of PSSS ranges from 12-84 points, scores ranging from 12 to 36 points indicate low perceived support, 37 to 60 points indicate intermediate perceived support, and 61 to 84 points indicate high perceived support. The Cronbach's alpha value of PSSS is 0.92. This scale has been applied to stroke patients in other studies [39,40].

The health behavior scale for stroke patients (HBS-SP)

The health behavior scale for stroke patients is a scale to assess the level of health behavior in patients with stroke. The scale was developed by Wan et al [41] in 2017. It includes 6 dimensions and 25 items. The exercise dimension contains six items, it refers to duration, frequency, type, intensity, plans, and motivation of exercise. There are 4 items in the medication-taking dimension, which means knowledge of taking medication and compliance. The instructions dimension (4 items) mainly includes adherence to physician's orders for moderate intake of salt, sugar, oil, etc. The responsibility dimension contains three items, which includes paying attention to ingredient lists on food package labels and monitoring heart rate during exercise. The nutrition dimension includes six items, which means eating appropriate amounts of meat, eggs, milk, grains,

For peer review only - http://bmjopen.bmj.com/site/about/guidelines.xhtml

fruits, soy products, etc. There are 2 items in the smoking and alcohol dimension, which implies appropriate levels of smoking and alcohol consumption. It is a 4-point Likert scale (from 1 = never to 4 = routinely performed). But the items of medication and tobacco, and alcohol were reversed. Higher scores indicate better health behavior. An average score of 2.5 points (between "sometimes" and "often") is considered an intermediate level of health behavior. The Cronbach's alpha value of the HBS-SP scale is 0.878.

Statistical analysis

The Statistical Package of Social Science software version 26 (IBM Corp. Released, 2017) was used to analyze the data. Participants' social demographics characteristics and other related variables were examined by descriptive statistics of frequencies and percentages. Pearson correlation analysis was used to analyze the interrelationships between the three variables. We applied the testing procedure recommended by Wen [42] and the mediation model was analyzed using Model 4 in the PROCESS Marco [43]. For the best test of the mediation effect, we carried out the bootstrapping procedure to measure the indirect effect, and a 95% confidence interval (*CI*) was estimated. $P<0.05$ was considered statistically significant. A 95% confidence interval excluding zero was regarded as statistically significant.

Patient and public involvement

Patients or the public were not involved in the design, conduct, reporting or dissemination of our research.

Results

General Demographics

A total of 254 questionnaires were obtained. However, 4 invalid questionnaires were removed because the questionnaires with a completion rate of less than 95%. Finally, the number of effective participants was 250, and the final effective rate of questionnaire collection was 98.4%. Over half (70.8%) of patients were elderly patients (≥60 years). Less than half of the patients in the sample had a primary school degree or less (46.1%). Moreover, 34.8% were working and a majority (97.6%) of patients with stroke were ischemic stroke. In addition, 50.4% of stroke patients were from low and middle-income groups. 52% of them were first-episode patients. A comparison of the scales' scores of stroke patients with different characteristics is shown in the Supplemental Table.

Descriptive statistics of the SRRPS, PSSS and HBS-SP

Table 1 displayed mean scores, standard deviations, and ranges of SRRPS, PSSS, and HBS-SP. The mean and SD of the stroke recurrence risk perception scale were 41.04±8.64 scores. The mean and SD of the perceived social support scale were 65.86±8.78 scores. The mean and SD of the perceived social support scale were 56.78±9.03 scores. Therefore, among the patients with stroke we recruited, recurrence risk perception and perceived social support were at high levels. Health behavior was at a moderately low level.

Table 1 Scores on the SRRPS, PSSS and HBS-SP

Variables	Items	Range of score	Mean	Stand deviation
SRRPS	17	17~51	41.04	8.64
perceived illness risk factors	4	6~18	19.96	4.76
perceived behavioral risk factors	6	4~12	22.80	3.35
perceived severity	7	7~21	17.84	4.05
PSSS	12	40~80	65.86	8.78

For peer review only - http://bmjopen.bmj.com/site/about/guidelines.xhtml

family support	4	13~28	23.08	3.58
friend support	4	7~28	19.97	4.77
other support	4	12~28	22.80	3.35
HBS-SP	25	28~81	56.78	9.03
exercise	6	5~24	11.83	4.81
medication taking	4	5~19	11.74	2.66
instructions	4	4~17	8.81	2.58
responsibility	3	3~8	3.25	3.91
nutrition	6	6~24	14.48	1.69
smoking and alcohol	2	2~8	6.66	0.73

Abbreviations: SRRPS, stroke recurrence risk perception scale; PSSS, Perceived social support scale; HBS-SP, Health behavior scale for stroke patients

Correlation Analysis between SRRPS, PSSS and HBS-SP

Pearson's correlation analysis was used to investigate the relationships among recurrence risk perception, health behavior, and perceived social support in patients with stroke. Briefly, recurrence risk perception was positively correlated with health behavior (r=0.679, $P < 0.001$), perceived social support was positively correlated with health behavior (r=0.778, $P < 0.001$), recurrence risk perception was positively correlated with perceived social support (r=0.639, $P < 0.001$), meaning that recurrence risk perception, health behavior, and perceived social support were positively correlated with each other in patients with stroke.

Mediation Analysis of SRRPS, PSSS and HBS-SP

Building on our initial findings from the correlation analysis, we next performed mediation analysis to explore the potential association underlying the relationship between an individual's perceived social support, their perception of stroke recurrence risk, and their adoption of healthy behaviors among stroke patients. We controlled for some general demographic factors (like age, gender, monthly income, and education) which are considered important predictors that affect health behavior in patients with stroke in China. In model 1, recurrence risk perception had a direct effect on health behavior (β= 0.607, $P < 0.001$). In model 2, recurrence risk perception had a direct effect on perceived social support (β= 0.604, $P < 0.001$). In model 3, recurrence risk perception and perceived social support positively related to health behavior (β= 0.247, β= 0.596, $P <$ 0.001), but the comparison of the effect coefficients showed that the effect of recurrence risk perception on health behavior was diminished from 0.607 to 0.247, which meant that perceived social support partially mediated the effect between recurrence risk perception and health behavior, as shown in Table 2.

Table 2 Mediation results for recurrence risk perception predicting health behavior mediated by perceived social support

Recurrence risk perception → Health behavior				
	SE	β	t	P
Age	0.968	-1.246	-1.287	0.200
Gender	0.863	0.958	1.110	0.268
Education	0.460	1.187	2.579	0.011
Monthly Income	0.742	1.158	1.561	0.120
Recurrence risk perception	0.053	0.607	11.529	<0.001
Recurrence risk perception →Perceived social support				

For peer review only - http://bmjopen.bmj.com/site/about/guidelines.xhtml

Gender	0.890	0.912	1.026	0.306
Education	0.474	1.669	3.522	0.005
Monthly Income	0.764	-1.514	-1.981	0.049
Recurrence risk perception	0.054	0.604	11.137	＜0.001
Recurrence risk perception, Perceived social support → Health behavior				
Age	0.766	-1.389	-1.812	0.071
Gender	0.685	0.415	0.607	0.545
Education	0.373	0.193	0.516	0.606
Monthly Income	0.592	2.059	3.480	0.006
Recurrence risk perception	0.051	0.247	4.833	＜0.001
Perceived social support	0.049	0.596	12.110	＜0.001

To ensure the accuracy of the test, this study further used the bootstrap method [43] to test the mediating effect of perceived social support between recurrence risk perception and health behavior with 5000 replicate samples. The results are shown in Table 3, and the 95% confidence interval of the mediating effect of perceived social support was [0.26,0.46], which did not contain zero, indicating that the mediating effect of perceived social support between recurrence risk perception and health behavior was established, and the effect accounted for 59.31% (Table 3). The mediating model of perceived social support is constructed in Figure 2.

Table 3 Mediating model examination by bootstrap

Recurrence risk perception → Health behavior				
	Effect	SE	LL 95%*CI*	UL 95%*CI*
Indirect effect	0.360	0.050	0.263	0.460
Direct effect	0.247	0.512	0.147	0.348

Abbreviations: SE, standard error; LL 95% CI, Lower Limit of 95% confidence interval; UL 95%CI, Upper Limit of 95% confidence interval

Insert Figure 2 here.

Discussion

We found that 78.4% of patients with stroke scored less than 2.5 on the health behavior scale, which meant they had moderately low health behavior. This finding was consistent with previous studies. Li et al [44] investigated 462 patients with stroke in three tertiary hospitals in Liaoning Province, China, and found that patients with stroke had moderate health behavior. The same results were reaffirmed in another study [45]. More than half of the 231 patients with stroke had moderately low health behavior. This highlighted the urgent need for further research and focus on the influencing factors of healthy behavior among stroke patients.

This study demonstrated that perceived social support was positively correlated with health behavior. This finding was consistent with our hypothesis 3 and previous studies [46,47]. A cross-sectional study [48] enrolled 133 hypertensive stroke patients 6 months after discharge in China has shown the perceptions of chronic illness resources received from healthcare teams, family and friends, and the community were positively correlated with health behavior. Tan [49] conducted a cross-sectional study among 350 stroke

For peer review only - http://bmjopen.bmj.com/site/about/guidelines.xhtml

participants in China and found that perceived social support had a direct positive effect on rehabilitation motivation. Moreover, Bandura's social theory [50] also proposed that factors such as social support were actual aspects of the incidence of behavior. Brouwer-Goossensen has found social support was a positive factor in health behavior change in interviews with 18 stroke patients [51]. Our study provided further evidence of the relationship between perceived social support and health behavior. Therefore, perceived social support is an important factor in improving health behavior in patients with stroke. In addition, there was a significant positive correlation between recurrence risk perception and perceived social support. As a result, our initial hypothesis 2 was contradicted by the findings of our study. In other words, perceived social support positively and significantly related to health behavior. We speculated the reason for this result was that the higher stroke patients' recurrence risk perception, the more control they had over their diseases. This sense of control over the disease could partly increase their self-efficacy [52] to stay in a good mind, promoting a positive mindset, and concurrently enhancing their perception of social support. Calderon et al [53] discovered that a majority of chronic patients expressed a desire for increased information and greater involvement in the decision-making process. To the best of our knowledge, our study was the first study to investigate the association between stroke patients' perceptions of recurrence and their perceived social support in China.

Most importantly, we found that recurrence risk perception appeared to correlate with health behavior via two pathways, a direct pathway and an indirect pathway mediated by perceived social support. (1) The first pathway, recurrence risk perception was correlated with health behavior significantly and positively. This was contrary to our research hypothesis 1. It may be because stroke patients did not view the risk of recurrence as a frightening event. They gradually increased their confidence in their recovery as they acquired more knowledge about the risk instead. In this way, they were better able to cope with the disease. For example, they began to adopt healthy behaviors to prevent recurrence. These findings were consistent with the health belief model (HBM) [18]. Besides, our previous research found patients with stroke who lacked a perception of the risk of recurrence and preventive strategies tended to limit their regular hospital visits or refrain from adopting healthy behaviors aimed at preventing recurrence [40]. In other words, recurrence risk perception was an important positive factor of health behavior. (2) The second pathway was that recurrence risk perception mediated the effect of perceived social support on health behavior. This was also contrary to our research hypothesis 4. To effectively implement secondary stroke prevention measures, patients with stroke must have a comprehensive understanding of the risk factors associated with recurrent stroke. Their understanding of the lifestyle changes needed to reduce the chance of future recurrences is equally crucial [54]. In patients with stroke, the perceived risk of recurrence involves not only just concern and fear but also the understanding of early warning signs and risk factors associated with stroke recurrence. So patients with stroke with a higher recurrence risk perception also demonstrated a greater depth of stroke knowledge [55], and these patients showed a higher level of confidence in recovery than patients with a lower perceived risk of recurrence. Therefore, they perceive information or emotional support from those around them better. The presence of these social supports played a significant role in improving their emotional well-being, consequently fostering a greater willingness to embrace healthier behaviors.

Overall, our study found that recurrence risk perception was not only directly and positively associated with the health behaviors of patients with stroke, but also further related to health behavior via perceived social support. This was consistent with the connotation of the CSM model [26]. In other words, our findings provided practical support for the CSM model. As the practical implication is that our finding may aid future research in identifying entry points for health behavior interventions by taking the methods that improve stroke patients' perception of recurrence risk and perceived social support. For example, healthcare providers could consider providing patients with recurrence risk communication. Specifically, healthcare professionals can help patients to have an accurate understanding of the risk of recurrence. First, a comparison between the subjective and objective risk perception scores for stroke recurrence is necessary for healthcare professionals. If the stroke recurrence risk perception and objective risk perception scores are inconsistent, then risk

For peer review only - http://bmjopen.bmj.com/site/about/guidelines.xhtml

communication should be initiated with stroke patients [56]. Common risk communication tools mainly include graphical [57] and numerical forms [58]. To enable patients to correctly grasp their rates of recurrence, appropriate risk communication strategies should be chosen according to their various levels of education and preferences. In addition, healthcare professionals can provide stroke patients with the necessary knowledge and skills to prevent recurrence, such as the aura of stroke recurrence, risk factors of recurrence, and prognosis of recurrence. By doing this, stroke patients' perceptions of social support can be enhanced, which will ultimately help them adopt healthy behaviors. It can also boost their confidence in their ability to recover.

The study still has some limitations. Firstly, it employed a cross-sectional design, thereby precluding the establishment of a causal relationship between recurrence risk perception and health behavior. Nonetheless, the study successfully confirmed a significant correlation between recurrence risk perception and health behavior. Secondly, despite our efforts to incorporate multi-center sampling, the impact of COVID-19 necessitated the restriction of our study to a single tertiary hospital in Henan Province. Therefore, the findings may not be generalizable to all patients with stroke. Besides, only six hemorrhagic patients with stroke were included in our study, so it was difficult for us to explore differences in stroke type in patients' perceived risk of recurrence, perceived social support, and health behavior. Lastly, our study included a relatively elderly population of patients with stroke, which may have some impact on the generalizability of the findings. Therefore, we recommend the design of a multi-center longitudinal study specifically focusing on young stroke patients in future research. Such a study design would help clarify the dynamic trends in recurrence risk perception, perceived social support, and health behavior over time.

Conclusion

In conclusion, this study found that perceived social support mediated the relationship between recurrence risk perception and health behavior. Our findings expand upon previous research on the interplay between recurrence risk perception, perceived social support, and health behavior in patients with stroke. The results of this study can serve as a useful guide for future clinical nursing practice and research in this area. Considering the importance of recurrence risk perception and perceived social support, it is recommended that these factors be incorporated into the assessment process for patients with stroke, both during hospitalization and follow-up care. We suggest that healthcare professionals should assist patients with stroke in developing an accurate perception of recurrence risk with appropriate recurrence risk communication approaches. This method can significantly improve patients' capacity to identify and access social support, ultimately enhancing their confidence in recovery and motivation to engage in healthy behaviors. Moreover, it is crucial to recognize the significance of social support from family and friends as a primary source of perceived social support. Healthcare professionals should encourage and educate patients' loved ones to offer emotional support, which can positively impact patients' recovery and well-being.

Acknowledgements

We would like to acknowledge the tertiary hospital of Henan province for their cooperation. We greatly appreciate the support of all patients for their voluntary participation in this study.

Contributors

ZZX and LBL designed the study. WXX, JH and WWN contributed to writing and revising. WXX, MYX and ZCH were in charge of data collection and analysis. ZQS and CSY were in charge of study design and essential help. All authors read and approved the final manuscript.

Funding

This work was supported by the National Natural Science Foundation of China Youth Science Foundation Project (72104221).

For peer review only - http://bmjopen.bmj.com/site/about/guidelines.xhtml

Competing interests

None declared.

Patient consent for publication

Each participant gave informed written consent and permission to publish before analysis of the study.

Ethics approval

The Research Ethics Committee of Zhengzhou University (ZZURIB2020-08) approved the study.

Provenance and peer review

Not commissioned; externally peer reviewed.

Data availability statement

Data are available upon reasonable request.

For peer review only

References

1 Chen Y, Wright N, Guo Y, *et al.* Mortality and recurrent vascular events after first incident stroke: a 9-year community-based study of 0·5 million Chinese adults. *Lancet Glob Health*. 2020;8:e580–90.

2 Gladstone DJ, Lindsay MP, Douketis J, *et al.* Canadian Stroke Best Practice Recommendations: Secondary Prevention of Stroke Update 2020. *Can J Neurol Sci*. 2022;49:315–37.

3 Kleindorfer DO, Towfighi A, Chaturvedi S, *et al.* 2021 Guideline for the Prevention of Stroke in Patients With Stroke and Transient Ischemic Attack: A Guideline From the American Heart Association/American Stroke Association. *Stroke*. 2021;52:e364–467.

4 Liu L, Chen W, Zhou H, *et al.* Chinese Stroke Association guidelines for clinical management of cerebrovascular disorders: executive summary and 2019 update of clinical management of ischaemic cerebrovascular diseases. *Stroke Vasc Neurol*. 2020;5:159–76.

5 O'Donnell MJ, Xavier D, Liu L, *et al.* Risk factors for ischaemic and intracerebral haemorrhagic stroke in 22 countries (the INTERSTROKE study): a case-control study. *Lancet*. 2010;376:112–23.

6 Abdullahi SU, Sunusi S, Abba MS, *et al.* Hydroxyurea for secondary stroke prevention in children with sickle cell anemia in Nigeria: a randomized controlled trial. *Blood*. 2023;141:825–34.

7 Liu Y, Wei M, Guo L, *et al.* Association between illness perception and health behaviour among stroke patients: The mediation effect of coping style. *Journal of Advanced Nursing*. 2021;77:2307–18.

8 Mugwano I, Kaddumukasa M, Mugenyi L, *et al.* Poor drug adherence and lack of awareness of hypertension among hypertensive stroke patients in Kampala, Uganda: a cross sectional study. *BMC Res Notes*. 2016;9:3.

9 Wu X, Min L, Cong L, *et al.* Sex differences in health-related quality of life among adult stroke patients in Northeastern China. *J Clin Neurosci*. 2014;21:957–61.

10 Kim G-G, Chae D-H, Park M-S, *et al.* Factors Influencing 1-Year Medication Adherence of Korean Ischemic Stroke Survivors. *Int J Behav Med*. 2020;27:225–34.

11 Parappilly BP, Field TS, Mortenson WB, *et al.* Determinants Influencing the Prestroke Health Behaviors and Cardiovascular Disease Risk of Stroke Patients: A Cross-Sectional Study. *J Stroke Cerebrovasc Dis*. 2019;28:1509–18.

For peer review only - http://bmjopen.bmj.com/site/about/guidelines.xhtml

12 Santos M, Kövari E, Gold G, *et al.* The neuroanatomical model of post-stroke depression: towards a change of focus? *J Neurol Sci*. 2009;283:158–62.

13 Brouwer-Goossensen D, Genugten L van, Lingsma H, *et al.* Determinants of intention to change health-related behavior and actual change in patients with TIA or minor ischemic stroke. *Patient Educ Couns*. 2016;99:644–50.

14 Ren H, Guo Y-F, Zhang Z-X, *et al.* Perception of recurrent risk versus objective measured risk of ischemic stroke in first-ever stroke patients from a rural area in China: A cross-sectional study. *Patient Educ Couns*. 2023;107:107586.

15 Holder C, Krishnamurthi R, Theadom A. Exploring facilitators and barriers to long-term behavior change following health-wellness coaching for stroke prevention: A qualitative study conducted in Auckland, New Zealand. *Brain Behav*. 2023;13:e2671.

16 Lin B, Zhang Z, Guo Y. Development and psychometric test of Recurrence Risk Perception Scale for Patients with Stroke. *Chinese Journal of Nursing*. 2022;56:1666–71.

17 Thakkar J, Heeley EL, Chalmers J, *et al.* Inaccurate risk perceptions contribute to treatment gaps in secondary prevention of cardiovascular disease. *Intern Med J*. 2016;46:339–46.

18 Champion VL, Skinner CS. The health belief model. *Health behavior and health education: Theory, research, and practice, 4th ed*. San Francisco, CA, US: Jossey-Bass 2008:45–65.

19 Iversen AB, Blauenfeldt RA, Johnsen SP, *et al.* Understanding the seriousness of a stroke is essential for appropriate help-seeking and early arrival at a stroke centre: A cross-sectional study of stroke patients and their bystanders. *Eur Stroke J*. 2020;5:351–61.

20 Freeman-Gibb LA, Janz NK, Katapodi MC, *et al.* The relationship between illness representations, risk perception and fear of cancer recurrence in breast cancer survivors. *Psychooncology*. 2017;26:1270–7. doi: 10.1002/pon.4143

21 Lee-Jones C, Humphris G, Dixon R, *et al.* Fear of cancer recurrence--a literature review and proposed cognitive formulation to explain exacerbation of recurrence fears. *Psychooncology*. 1997;6:95–105. doi: 10.1002/(SICI)1099-1611(199706)6:2<95::AID-PON250>3.0.CO;2-B

22 Blanton S, Scheibe DC, Rutledge AH, *et al.* Family-Centered Care During Constraint-Induced Therapy After Chronic Stroke: A Feasibility Study. *Rehabil Nurs*. 2019;44:349–57.

23 Northcott S, Moss B, Harrison K, *et al.* A systematic review of the impact of stroke on social support and social networks: associated factors and patterns of change. *Clin Rehabil*. 2016;30:811–31.

24 Kruithof WJ, van Mierlo ML, Visser-Meily JMA, *et al.* Associations between social support and stroke survivors' health-related quality of life--a systematic review. *Patient Educ Couns*. 2013;93:169–76.

25 Dahlan R, Ghazal E, Saltaji H, *et al.* Impact of social support on oral health among immigrants and ethnic minorities: A systematic review. *PLoS One*. 2019;14:e0218678.

26 Leventhal H, Phillips LA, Burns E. The Common-Sense Model of Self-Regulation (CSM): a dynamic framework for understanding illness self-management. *J Behav Med*. 2016;39:935–46. doi: 10.1007/s10865-016-9782-2

27 Leventhal H, Diefenbach M, Leventhal E. Illness cognition: Using common sense to understand treatment adherence and affect cognition interactions. *Cognitive Therapy and Research*. 1992;16:143–63.

28 Hagger MS, Orbell S. The common sense model of illness self-regulation: a conceptual review and proposed extended model. *Health Psychol Rev*. 2022;16:347–77.

29 Fall E, Chakroun-Baggioni N, Böhme P, *et al.* Common sense model of self-regulation for understanding adherence and quality of life in type 2 diabetes with structural equation modeling. *Patient Educ Couns*. 2021;104:171–8. doi: 10.1016/j.pec.2020.06.023

30 Johnston KN, Burgess R, Kochovska S, *et al.* Exploring the Experience of Breathlessness with the Common-Sense Model of Self-Regulation (CSM). *Healthcare (Basel)*. 2023;11:1686.

31 Adamson M, Cotoc C, Choi K, *et al.* Cancer Communication Outside of the Physician-Patient Relationship: The Experience of Communicating and Understanding the Meaning of Prognosis. *Am J Hosp Palliat Care*. 2019;36:711–7.

32 Singh T, Newby DE. Is the fear of disease worse than the disease itself? *Heart*. 2021;107:91–2.

33 Neurology CS of, Society CS. Diagnostic criteria of cerebrovascular diseases in China (version 2019). *Chinese Journal of Neurology*. 2019;52:710–5. doi: 10.3760/cma.j.issn.1006-7876.2019.09.003

For peer review only

34 Gao C, Wu C, Zhao J, *et al.* Guidelines for the Diagnosis and Treatment of Cerebral Infarction in China (2017). *Chinese Journal of Integrated Traditional and Western Medicine*. 2018;38:136–44.

35 Ni P, Chen J, Liu N. Sample size estimation in quantitative nursing research. *Chinese Journal of Nursing*. 2010;45:378–80.

36 Tang B, Lv Y, Wang X, *et al.* Analysis of status and influencing factors of recurrence risk perception in first-episode ischemic stroke patients. *Chinese Journal of Nursing*. 2023;58:289–95.

37 Gao F, An Q, Liu D, *et al.* Recurrence risk perception and its correlates in stroke patients. *Journal of Nursing(China)*. 2023;38:30–3.

38 Wang X, Wang X, Ma H. *Manual of the Mental Health Rating Scale*. Beijing: Chinese Journal of Mental Health 1999.

39 Dong X, Yang S, Guo Y, *et al.* Exploring Psychoneurological Symptom Clusters in Acute Stroke Patients: A Latent Class Analysis. *J Pain Res*. 2022;15:789–99.

40 Wang X, Shang S, Yang H, *et al.* Associations of psychological distress with positive psychological variables and activities of daily living among stroke patients: a cross-sectional study. *BMC Psychiatry*. 2019;19:381.

41 Wan L, Xiong X, Pan J, *et al.* Development and reliability as well as validity test of Health Behavior Scale for stroke patients. *Journal of Nursing Science*. 2017;32:25–9.

42 Wen Z. Analyses of Mediating Effects: The Development of Methods and Models. *Advances in psychological Science*. 2014;22:731–45.

43 Henderson AR. The bootstrap: a technique for data-driven statistics. Using computer-intensive analyses to explore experimental data. *Clin Chim Acta*. 2005;359:1–26.

44 Li S, He H, Li H, *et al.* Chain Mediating Effects of Executive Intention of Rehabilitation Exercise Behaviors and Mental Health Literacy between Perceived Social Support and Health Behaviors among Stroke Inpatients. *Military Nursing*. 2023;40:34–8.

45 Liu M, Liang F, Yin Y, *et al.* Latent Profile Analysis of Health Literacy and Comparison of Health Behavior Differences in Patients with Stroke. *Military Nursing*. 2023;40:52-55+70.

46 Denche-Zamorano A, Pisà-Canyelles J, Barrios-Fernandez S, *et al.* Is Psychological Distress Associated with Self-Perceived Health, Perceived Social Support and Physical Activity Level in Spanish Adults with Diabetes? *J Pers Med*. 2023;13:739.

47 Guo A, Jin H, Mao J, *et al.* Impact of health literacy and social support on medication adherence in patients with hypertension: a cross-sectional community-based study. *BMC Cardiovasc Disord*. 2023;23:93. doi: 10.1186/s12872-023-03117-x

48 Wen K, Shen M-J, Deng C-S, *et al.* Association between chronic illness resources and health behaviors in hypertensive stroke patients at 6 months after discharge: a cross-sectional study. *Ann Transl Med*. 2022;10:460.

49 Tan M, Li H, Wang X. Analysis of the current status of rehabilitation motivation and its influencing factors in older adults with stroke: a cross-sectional study. *Front Aging Neurosci*. 2023;15:1186681.

50 Bandura A. Social cognitive theory: an agentic perspective. *Annu Rev Psychol*. 2001;52:1–26.

51 Brouwer-Goossensen D, den Hertog HM, Mastenbroek-de Jong MA, *et al.* Patient perspectives on health-related behavior change after transient ischemic attack or ischemic stroke. *Brain Behav*. 2021;11:e01993. doi: 10.1002/brb3.1993

52 Al Shafaee MA, Ganguly SS, Al Asmi AR. Perception of stroke and knowledge of potential risk factors among Omani patients at increased risk for stroke. *BMC Neurol*. 2006;6:38.

53 Calderon C, Jiménez-Fonseca P, Ferrando PJ, *et al.* Psychometric properties of the Shared Decision-Making Questionnaire (SDM-Q-9) in oncology practice. *Int J Clin Health Psychol*. 2018;18:143–51.

54 Hiraga A. Perception of recurrent stroke risk among stroke survivors. *Neuroepidemiology*. 2011;37:88–9.

55 Slark J, Bentley P, Majeed A, *et al.* Awareness of stroke symptomatology and cardiovascular risk factors amongst stroke survivors. *J Stroke Cerebrovasc Dis*. 2012;21:358–62.

56 Chen DW, Reyes-Gastelum D, Wallner LP, *et al.* Disparities in risk perception of thyroid cancer recurrence and death. *Cancer*. 2020;126:1512–21. doi: 10.1002/cncr.32670

57 Goodyear-Smith F, Arroll B, Chan L, *et al.* Patients Prefer Pictures to Numbers to Express Cardiovascular Benefit From

For peer review only - http://bmjopen.bmj.com/site/about/guidelines.xhtml

Treatment. *The Annals of Family Medicine*. 2008;6:213–7.

58 Fair AKI, Murray PG, Thomas A, *et al.* Using hypothetical data to assess the effect of numerical format and context on the perception of coronary heart disease risk. *American Journal of Health Promotion*. 2008;22:291–6.

Figure 1 The hypothesis model of recurrence risk perception, health behavior and perceived social support in patients with stroke

Figure 2 Mediating effects of perceived social support on the relationship between recurrence risk perception and health behavior (*$P < 0.001$)

For peer review only

For peer review only - http://bmjopen.bmj.com/site/about/guidelines.xhtml

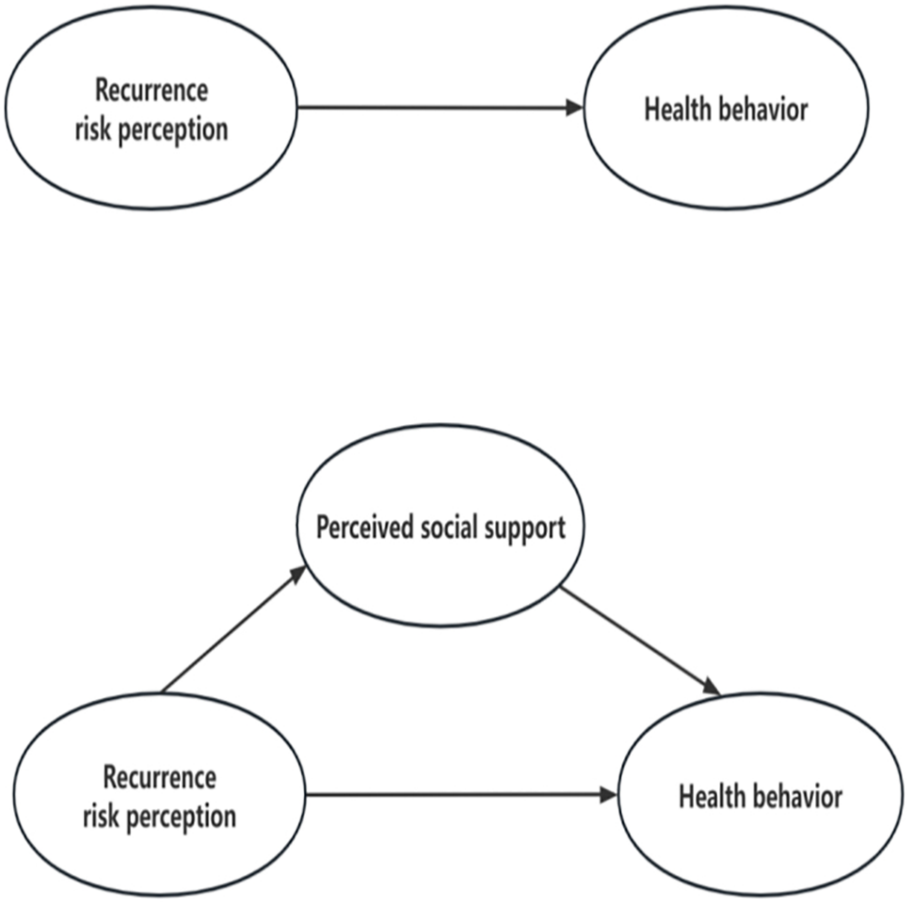

90x90mm (300 x 300 DPI)

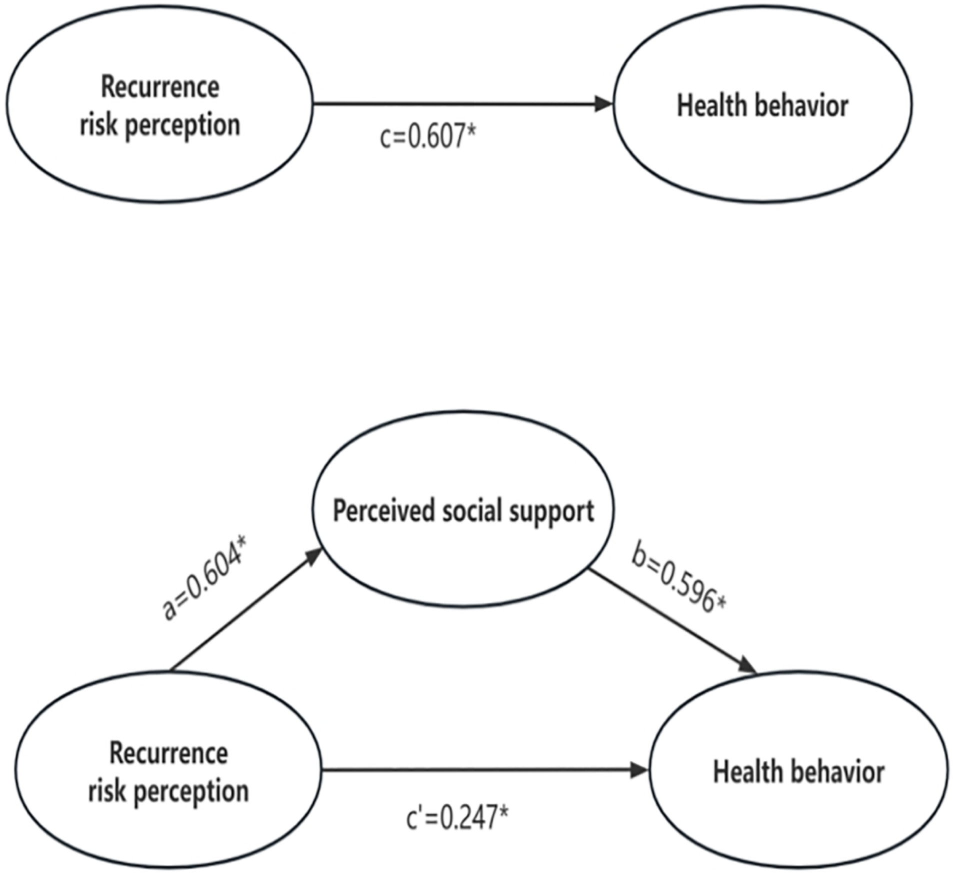

90x90mm (500 x 500 DPI)

Supplemental Table 1 Comparison of recurrence risk perception scores of stroke patients with different characteristics

Factors	Group	*N*	%	SRRPS	Perceived illness risk factors	Perceived behavioral risk factors	Perceived severity
Gender	Male	136	54.4	40.88±9.73	14.68±4.03	8.71±2.38	17.43±4.45
	Female	114	45.6	41.23±7.15	14.66±3.60	8.24±2.30	18.33±3.48
Marriage	Married	220	88	41.46±8.23	14.92±3.71	8.60±2.29	17.95±3.85
	Divorced	2	0.8	47.00±0.00	17.00±1.41	9.50±2.12	20.50±0.71
	Unmarried	28	11.2	37.29±10.91	12.50±4.24	7.71±2.79	16.82±5.40
Age	<60	73	29.2	44.55±6.44	15.93±2.83	9.59±2.06	19.03±2.93
	≥60	177	70.8	39.59±9.02	14.15±4.07	8.04±2.32	17.84±4.05
Residential area	Rural	126	50.4	39.92±7.91	14.08±3.91	8.10±2.17	17.68±3.79
	Urban	124	49.6	42.17±9.21	15.27±3.68	8.89±2.46	18.01±4.32
Working state	Unemployed	54	21.6	40.11±7.29	13.48±3.30	7.85±2.28	18.78±3.66
	Pensioner	109	43.6	39.37±9.98	14.16±4.22	8.18±2.47	17.03±4.66
	Working	87	34.8	43.70±6.85	16.06±3.22	9.28±2.04	18.29±3.23
Educational level	Primary and below	115	46	38.72±7.99	13.56±3.97	7.82±2.06	17.29±4.06
	Junior high school	65	27.6	40.49±8.61	14.75±3.35	8.22±2.28	17.52±4.28
	High school	44	17.6	42.81±9.35	15.50±3.99	9.11±2.46	18.20±4.18
	College	14	5.6	48.64±2.21	18.00±0.00	10.57±1.60	20.07±2.06
	University and above	12	4.8	50.75±0.45	18.00±0.00	11.75±0.45	21.00±0.00
Stroke type	Hemorrhagic stroke	6	2.4	47.50±3.74	17.25±1.04	10.00±2.14	20.25±1.04
	Ischemic stroke	244	97.6	40.82±8.67	14.59±3.86	8.44±2.34	17.76±4.09

Family history	Have	25	10	38.36±10.60	13.32±4.96	8.08±2.33	16.96±4.33
	No	255	90	41.33±8.37	14.82±3.67	8.54±2.35	17.94±4.02
Monthly income (RMB)	≤2999	89	35.6	37.62±7.98	13.08±3.81	7.56±2.00	16.90±4.22
	3000-4999	126	50.4	42.59±8.39	15.52±3.64	8.75±2.27	18.32±3.90
	≥5000	35	14	44.14±8.56	15.69±3.31	9.91±2.56	18.54±3.85
Numbers of stroke	1 time	130	52	41.54±8.19	15.38±3.42	8.78±2.05	17.38±3.99
	2 times	74	29.6	43.15±6.12	15.04±3.18	8.88±2.16	19.23±2.70
	3 times	22	8.8	42.27±8.14	14.27±4.08	8.27±3.01	19.41±3.70
	More than 3 times	24	9.6	30.67±11.07	10.08±4.57	5.92±2.30	14.67±5.73
Primary caregiver	Spouse or children	132	52.8	17.30±4.46	14.30±4.16	8.17±2.42	39.82±9.54
	Babysitter or nursing workers	11	4.4	38.00±11.12	14.73±4.47	14.73±4.47	15.73±5.10
	Own	107	42.8	42.85±6.71	15.12±3.31	8.99±2.22	18.74±3.15
Number of chronic diseases	0	75	30	40.15±8.96	14.07±3.80	8.15±2.15	17.93±3.94
	1~2	150	60	41.29±9.17	14.75±4.04	8.67±2.43	17.83±4.17
	3 and above	25	10	42.20±6.87	16.04±1.86	8.48±2.40	17.68±3.87
Sleep conditions	Very good	92	36.8	40.07±9.91	14.12±4.20	8.35±2.55	17.52±4.60
	Good	69	27.6	42.51±8.25	15.25±3.78	8.81±2.37	18.45±3.47
	Poor	69	27.6	39.65±7.86	14.23±3.61	8.28±2.28	17.14±4.21
	Very poor	20	8	45.20±2.19	16.75±1.55	8.80±1.32	19.65±0.88

Supplemental Table 2 Comparison of perceived social support scores of stroke patients with different characteristics

Factors	Group	*N*	%	PSSS	Family support	Friend support	Other support
Gender	Male	136	54.4	65.50±9.07	22.65±3.89	20.43±4.32	22.42±3.48
	Female	114	45.6	66.29±8.43	23.60±3.11	19.41±5.22	23.28±3.15
Marriage	Married	220	88	66.27±8.55	23.17±3.49	20.15±4.84	22.95±3.15
	Divorced	2	0.8	69.00±12.73	22.50±3.54	23.00±4.24	23.50±4.95
	Unmarried	28	11.2	62.43±9.88	22.39±4.26	18.36±3.96	21.68±4.60
Gender	＜60	73	29.2	68.64±8.56	23.90±2.92	21.11±5.21	23.63±2.74
	≥60	177	70.8	64.71±8.63	22.75±3.78	19.50±4.51	22.47±3.52
Residential area	Rural	126	50.4	65.54±8.14	23.23±3.08	19.37±4.90	22.94±3.31
	Urban	124	49.6	66.19±9.40	22.94±4.04	20.58±4.57	22.67±3.40
Working state	Unemployed	54	21.6	64.33±8.70	22.67±3.39	19.57±4.96	22.09±3.54
	Pensioner	109	43.6	65.01±8.81	22.76±3.82	19.71±4.78	22.54±3.34
	Working	87	34.8	67.87±8.51	23.75±3.32	20.54±4.64	23.59±3.12
Educational level	Primary and below	115	46	64.03±7.79	22.57±3.31	18.92±4.90	22.53±3.06
	Junior high school	65	27.6	64.07±8.83	22.32±3.79	19.77±4.17	21.97±3.82
	High school	44	17.6	68.37±8.74	23.80±3.57	20.95±4.73	23.61±3.19
	College	14	5.6	71.36±7.96	25.36±3.03	21.29±3.95	24.71±3.20
	University and above	12	4.8	77.58±4.54	26.83±1.57	25.92±.151	25.83±2.04
Stroke type	Hemorrhagic stroke	6	2.4	73.88±7.38	26.50±1.60	21.63±5.58	25.75±1.16
	Ischemic stroke	244	97.6	65.60±8.71	22.97±3.57	19.91±4.74	22.71±3.36
Family history	Have	25	10	66.00±8.56	23.28±2.57	19.24±5.33	23.49±3.38
	No	255	90	65.84±8.82	23.06±3.68	20.05±4.71	22.73±3.35
Monthly income (RMB)	≤2999	89	35.6	64.73±8.07	22.79±3.48	19.10±4.46	33.84±3.44

	3000-4999	126	50.4	65.67±8.59	23.09±3.46	19.94±5.01	22.65±3.08
	≥5000	35	14	69.40±10.38	23.83±4.22	22.23±3.92	23.29±4.06
Numbers of stroke	1 time	130	52	65.88±9.05	22.90±3.62	20.18±4.84	22.80±3.35
	2 times	74	29.6	67.03±7.36	23.88±2.91	20.18±4.63	22.97±2.93
	3 times	22	8.8	67.27±9.19	23.59±3.65	20.32±4.47	23.36±3.47
	More than 3 times	24	9.6	60.83±9.63	21.17±4.45	17.83±4.82	21.83±4.34
Primary caregiver	Spouse or children	132	52.8	64.95±8.77	23.23±3.77	18.86±5.03	22.86±3.54
	Babysitter or nursing workers	11	4.4	58.73±8.00	19.64±3.80	19.55±1.51	19.55±4.25
	Own	107	42.8	67.71±8.37	23.25±3.15	21.37±4.29	23.08±2.83
Number of chronic diseases	0	75	30	66.16±7.92	23.17±3.38	19.52±5.07	23.47±3.04
	1~2	150	60	65.12±9.16	22.78±3.72	20.05±4.63	22.29±3.51
	3 and above	25	10	69.40±8.24	24.64±2.97	20.84±4.67	23.92±2.69
Sleep conditions	Very good	92	36.8	65.48±9.27	22.83±4.07	19.86±5.14	22.79±3.34
	Good	69	27.6	68.55±8.27	24.11±3.00	20.75±4.73	23.68±2.96
	Poor	69	27.6	63.55±8.66	22.29±3.53	19.22±4.54	22.04±3.81
	Very poor	20	8	66.30±6.16	23.45±2.26	20.35±3.66	22.50±2.40

Supplemental Table 3 Comparison of health behavior scores of stroke patients with different characteristics

Factors	Group	*N*	%	HBS-SP	Exercise	Medication taking	Instructions	Nutrition	Responsibility	Smoking and alcohol
Gender	Male	136	54.4	56.72±9.07	11.71±4.6	12.00±2.73	8.65±2.48	14.37±4.03	3.30±0.83	6.68±1.59
	Female	114	45.6	56.84±9.01	11.97±5.01	11.42±2.55	9.00±2.70	14.61±3.77	3.19±0.58	6.63±1.82
Marriage	Married	220	88	57.12±8.84	12.04±4.79	11.72±2.55	8.94±2.66	14.60±3.91	3.25±0.74	6.57±1.73
	Divorced	2	0.8	59.50±3.54	12.50±0.71	11.50±0.71	10.00±2.83	15.00±1.41	3.50±0.71	7.00±1.41
	Unmarried	28	11.2	53.86±10.33	10.10±4.86	11.82±3.53	7.93±1.68	13.29±3.80	3.32±0.67	7.39±1.13
Age	＜60	73	29.2	60.47±9.38	13.88±5.14	12.23±2.58	8.60±2.72	15.88±3.83	3.45±1.09	6.42±1.79
	≥60	177	70.8	55.25±8.44	10.99±4.42	11.54±2.67	8.90±2.52	13.90±3.80	3.17±0.49	6.76±1.65
Residential area	Rural	126	50.4	55.83±7.44	11.84±4.69	11.55±2.63	8.84±2.27	13.93±3.40	3.17±0.72	6.49±1.77
	Urban	124	49.6	57.74±10.34	11.82±4.95	11.94±2.68	8.78±2.86	15.04±4.31	3.33±0.74	6.83±1.59
Working state	Unemployed	54	21.6	55.30±8.14	12.43±4.90	11.37±2.53	8.54±2.34	13.67±3.19	3.00±0.00	6.30±1.89
	Pensioner	109	43.6	55.55±8.75	10.81±4.49	11.55±2.56	8.95±2.74	14.23±4.16	3.18±0.49	6.83±1.52
	Working	87	34.8	59.23±9.46	12.75±4.96	12.21±2.82	8.82±2.54	15.30±3.87	3.49±1.06	6.67±1.75
Educational level	Primary and below	115	46	54.56±7.09	11.47±4.47	11.13±2.53	8.69±2.23	13.47±3.27	3.08±0.35	6.72±1.76
	Junior high school	65	27.6	55.14±8.66	11.46±4.06	11.65±2.78	8.83±2.45	13.75±3.96	3.11±0.31	6.34±1.71
	High school	44	17.6	58.80±9.69	10.61±5.11	12.57±2.73	8.39±3.33	16.64±4.37	3.27±0.62	7.32±1.07
	College	14	5.6	63.64±8.72	15.43±3.82	13.07±2.06	9.86±3.08	16.07±3.00	3.57±1.01	5.64±2.27

	University and above	12	4.8	71.50±7.01	17.58±6.42	13.50±1.62	10.25±2.22	18.33±2.53	5.25±1.60	6.58±1.16
Stroke type	Hemorrhagic stroke	6	2.4	64.75±7.94	13.13±6.49	13.13±2.17	11.75±2.05	16.38±2.92	3.00±0.00	7.38±0.91
	Ischemic stroke	244	97.6	56.51±8.95	11.79±4.76	11.69±2.67	8.71±2.54	14.42±3.93	3.26±0.74	6.63±1.71
Family history	Have	25	10	57.68±9.39	13.00±3.81	11.00±2.02	10.16±2.73	13.76±4.15	3.12±0.33	6.64±1.63
	No	255	90	56.68±9.00	11.70±4.90	11.82±2.71	8.66±2.53	14.56±3.88	3.27±0.76	6.66±1.70
Monthly income (RMB)	≤2999	89	35.6	53.47±6.64	10.85±4.20	11.28±2.69	8.58±2.48	13.12±3.46	3.03±0.18	6.60±1.87
	3000-4999	126	50.4	57.63±8.78	12.09±4.89	11.87±2.47	8.94±2.76	14.85±3.84	3.26±0.67	6.63±1.65
	≥5000	35	14	62.11±11.78	13.40±5.54	12.46±3.07	8.94±2.14	16.60±4.08	3.77±1.33	6.94±1.37
Numbers of stroke	1 time	130	52	57.08±9.51	11.77±4.59	11.77±2.73	8.40±2.71	15.00±4.12	3.34±0.87	6.80±1.65
	2 times	74	29.6	57.69±7.11	13.11±4.78	11.58±2.32	9.23±2.41	14.23±3.02	3.18±0.53	6.36±1.72
	3 times	22	8.8	59.32±9.24	12.64±5.48	12.95±2.50	9.82±2.26	14.64±3.17	3.27±0.77	6.00±2.00
	More than 3 times	24	9.6	50.00±8.90	7.50±2.72	10.96±3.13	8.83±2.32	12.29±5.01	3.00±0.00	7.42±1.10
Primary caregiver	Spouse or children	132	52.8	55.58±8.50	10.40±4.48	11.95±2.78	8.63±2.70	14.67±4.31	3.24±0.80	6.69±1.69
	Babysitter or nursing workers	11	4.4	52.36±10.27	9.64±2.87	9.45±2.34	9.18±2.27	12.91±3.36	3.73±0.79	7.45±1.04
	Own	107	42.8	58.71±9.19	13.82±4.66	11.72±2.44	9.00±2.46	14.41±3.40	3.22±0.61	6.54±1.74
Number of chronic	0	75	30	57.47±7.61	12.75±4.69	12.16±2.38	8.92±2.15	14.12±3.11	3.16±0.55	6.36±1.61

diseases										
	1~2	150	60	56.07±9.44	11.35±4.75	11.51±2.84	8.93±2.50	14.34±4.19	3.21±0.61	6.73±1.75
	3 and above	25	10	58.96±10.18	12.00±5.30	11.88±2.20	7.76±3.82	16.40±3.88	3.76±1.42	7.16±1.46
Sleep conditions	Very good	92	36.8	56.11±9.22	11.86±4.79	11.83±2.94	8.68±2.64	13.79±3.91	3.26±0.66	6.68±1.52
	Good	69	27.6	59.35±9.22	12.68±4.70	11.97±2.39	9.23±2.60	15.38±3.88	3.30±0.67	6.78±1.61
	Poor	69	27.6	55.14±9.16	11.12±4.82	11.38±2.63	8.42±2.45	14.45±4.07	3.26±0.94	6.52±2.01
	Very poor	20	8	56.60±4.51	11.25±5.15	11.80±2.33	9.30±2.60	14.65±2.91	3.00±0.00	6.60±1.64

For peer review only

STROBE 2007 (v4) checklist of items to be included in reports of observational studies in epidemiology*

Checklist for cohort, case-control, and cross-sectional studies (combined)

	Item No	Recommendation	Page
**Title and abstract**	1	(*a*) Indicate the study's design with a commonly used term in the title or the abstract	Page 1
		(*b*) Provide in the abstract an informative and balanced summary of what was done and what was found	Page 1
**Introduction**			
Background/rationale	2	Explain the scientific background and rationale for the investigation being reported	Page 1-3
Objectives	3	State specific objectives, including any prespecified hypotheses	Page 3
**Methods**			
Study design	4	Present key elements of study design early in the paper	Page 3
Setting	5	Describe the setting, locations, and relevant dates, including periods of recruitment, exposure, follow-up, and data collection	Page 3
Participants	6	(*a*) Give the eligibility criteria, and the sources and methods of selection of participants	Page 3
Variables	7	Clearly define all outcomes, exposures, predictors, potential confounders, and effect modifiers. Give diagnostic criteria, if applicable	Page 6
Data sources/ measurement	8*	For each variable of interest, give sources of data and details of methods of assessment (measurement). Describe comparability of assessment methods if there is more than one group	Page 4
Bias	9	Describe any efforts to address potential sources of bias	Page 8-9
Study size	10	Explain how the study size was arrived at	Page 3
Quantitative variables	11	Explain how quantitative variables were handled in the analyses. If applicable, describe which groupings were chosen and why	Page 4
Statistical methods	12	(*a*) Describe all statistical methods, including those used to control for confounding	Page 4
		(*b*) Describe any methods used to examine subgroups and interactions	Not applicable
		(*c*) Explain how missing data were addressed	Page 3
		(*d*) If applicable, describe analytical methods taking account of sampling strategy	Not applicable
		(*e*) Describe any sensitivity analyses	Not applicable
**Results**			

For peer review only

For peer review only - http://bmjopen.bmj.com/site/about/guidelines.xhtml

Participants	13*	(a) Report numbers of individuals at each stage of study—eg numbers potentially eligible, examined for eligibility, confirmed eligible, included in the study, completing follow-up, and analysed	Not applicable
		(b) Give reasons for non-participation at each stage	Not applicable
		(c) Consider use of a flow diagram	Not applicable
Descriptive data	14*	(a) Give characteristics of study participants (eg demographic, clinical, social) and information on exposures and potential confounders	Page 5
		(b) Indicate number of participants with missing data for each variable of interest	Page 5
Outcome data	15*	Report numbers of outcome events or summary measures	Page 5
Main results	16	(*a*) Give unadjusted estimates and, if applicable, confounder-adjusted estimates and their precision (eg, 95% confidence interval). Make clear which confounders were adjusted for and why they were included	Page 6
		(*b*) Report category boundaries when continuous variables were categorized	Page 5
		(*c*) If relevant, consider translating estimates of relative risk into absolute risk for a meaningful time period	Not applicable
Other analyses	17	Report other analyses done—eg analyses of subgroups and interactions, and sensitivity analyses	Not applicable

For peer review only

**Discussion**			
Key results	18	Summarise key results with reference to study objectives	Page 7
Limitations	19	Discuss limitations of the study, taking into account sources of potential bias or imprecision. Discuss both direction and magnitude of any potential bias	Page 8
Interpretation	20	Give a cautious overall interpretation of results considering objectives, limitations, multiplicity of analyses, results from similar studies, and other relevant evidence	Page 8
Generalisability	21	Discuss the generalisability (external validity) of the study results	Page 8
**Other information**			
Funding	22	Give the source of funding and the role of the funders for the present study and, if applicable, for the original study on which the present article is based	Page 9

*Give information separately for exposed and unexposed groups.

For peer review only
